# A Crystalline Mono‐Coordinate Indium(I)‐Phosphaalkenyl

**DOI:** 10.1002/anie.202523125

**Published:** 2026-01-15

**Authors:** Álvaro García‐Romero, Maren Pink, Israel Fernández, Jose M. Goicoechea

**Affiliations:** ^1^ Department of Chemistry Indiana University 800 East Kirkwood Ave. Bloomington Indiana 47405 USA; ^2^ Departamento de Química Orgánica and Centro de Innovación en Química Avanzada (ORFEO‐CINQA), Universidad Complutense de Madrid Facultad de Ciencias Químicas Madrid 28040 Spain

**Keywords:** Carbometallation, Gallium, Indium(I), Phosphaalkene, Phosphorus

## Abstract

The synthesis of the mono‐coordinate indium(I) compound In[C(Ad)═PTer] (Ad = 1‐adamantyl, Ter = 2,6‐Dipp_2_‐C_6_H_3_; Dipp = 2,6‐diisopropylphenyl) is reported. Key to the formation of this monomeric species is the steric protection offered by the supporting phosphaalkenyl ligand which hinders aggregation. The title compound can be accessed from the reaction of (InTer)_2_ with the phosphaalkyne AdC≡P in an unusual redox‐neutral transformation in which the In─C bond of the (InTer)_2_ precursor, known to dissociate in solution, adds across the C≡P triple bond of the phosphaalkyne. This insertion reaction is reversible, as shown by the reaction of In[C(Ad)═PTer] with B(C_6_F_5_)_3_, which affords [TerInB(C_6_F_5_)_3_] accompanied by extrusion of AdC≡P. In contrast, the lighter analogue (GaTer)_2_ promotes the dimerization of the AdC≡P fragment resulting in the formation of the cluster (GaTer)_2_(AdCP)_2_. The formal oxidation state of In[C(Ad) = PTer] was probed by reaction with methyl‐iodide which affords the indium(III) compound In(Me)I[C(Ad) = PTer] in a formal single‐site oxidative‐addition reaction at indium.

## Graphical abstract

The redox neutral carbometallation of a phospha‐alkyne using the dimeric indium(I) compound (InTer)_2_ is reported. This reaction affords a mono‐coordinate indium(I) compound that can reversibly extrude the phospha‐alkyne upon addition of a Lewis acid.

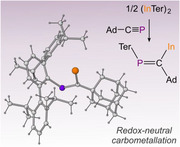

## Introduction

Compounds in which a metal is bonded to a single R‐type ligand are amongst the earliest organometallic species reported (e.g., MeLi).^[^ ^1^ ^]^ These compounds typically aggregate to form clusters, the nuclearity of which is governed by the organic substituents, solvent, and the metals themselves. Research in this area has primarily focused on metals with a propensity to adopt the +1 oxidation state: alkali‐metals, copper, silver and gold.^[^ ^2^ ^]^ That being said, the strong σ‐donor properties of carbanionic ligands can be used to stabilize (MR)_n_ compounds of elements for which the +1 oxidation is typically inaccessible. For example, cyclopentadienyl‐based ligands allow access to aluminium, gallium and indium in the +1 oxidation state such as (AlCp*)_4_ (Cp* = C_5_Me_5_).^[^ ^3^, ^4^, ^5^ ^]^ The isolation of monomeric MR species, however, necessitates extremely bulky organic substituents.

**Figure 1:**
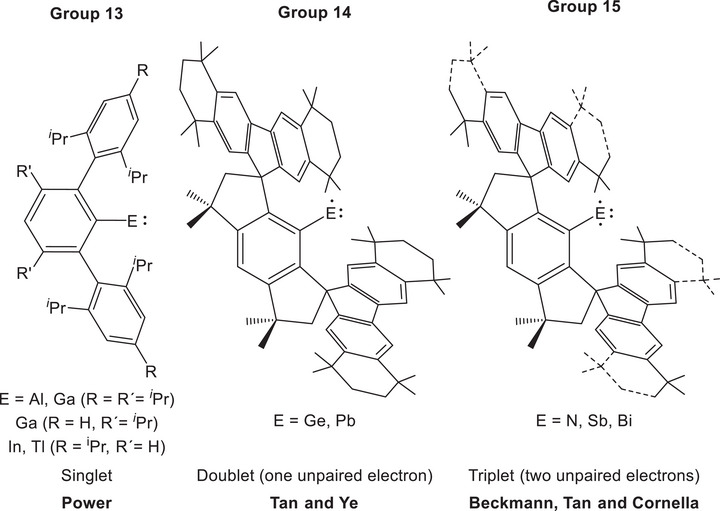
Selected examples of mono‐coordinate main group element compounds.

Power's pioneering use of terphenyl‐based substituents in organometallic chemistry allowed access to landmark examples of monomeric, mono‐coordinate compounds of the group 13 elements (E = Al–Tl; Figure 1, left).^[^ ^6^, ^7^, ^8^, ^9^ ^]^ It is worth noting that subtle changes to the ligand backbone can have a profound effect on the degree of aggregation of such compounds.^[^ ^7^, ^10^, ^11^, ^12^, ^13^ ^]^ Many compounds that adopt dimeric structures in the solid state, such as (InTer)_2_ (Ter = 2,6‐Dipp_2_‐C_6_H_3_; Dipp = 2,6‐diisopropylphenyl), are known to undergo partial dissociation in solution. Jones and co‐workers have also reported on the isolation of monomeric mono‐coordinate indium(I) and thallium(I) compounds supported by bulky amine ligands.^[^ ^14^, ^15^ ^]^ Related to these monomeric compounds is a more recent example by the group of Kretschmer of a singly bonded gallium(I) compound supported by a bulky hindrandacenyl ligand.^[^ ^16^ ^]^ Likewise, Krossing and co‐workers recently reported on the structural characterization of a cationic mono‐coordinate indium(I) species [In(CDP^Ph^)]^+^ (CDP^Ph^ = C(PPh_3_)_2_), however this compound readily decomposes even at −40 °C and, according to the authors, its synthesis could not be reliably reproduced.^[^ ^17^ ^]^ Cationic compounds of the group 14 elements supported by bulky carbazolyl ligands that are valence isoelectronic to these species have also been reported.^[^ ^18^, ^19^ ^]^

Isolation of monomeric MR compounds of the group 14 and 15 elements is inherently more challenging as the +1 oxidation state is highly disfavored. Power has shown that dimeric *trans*‐bent dimetallynes such as the distannyne TerSn≡SnTer^[^ ^20^ ^]^ can dissociate in solution into the corresponding Sn(I) radical SnTer.^[^ ^21^ ^]^ A major recent breakthrough in the isolation of monomeric MR compounds of the p‐block elements has been the use of bulky hindrandacenyl ligands. For example, Tan and Ye have used these ligands to access monomeric germanium(I) and lead(I) radicals (Figure 1, center).^[^ ^22^, ^23^ ^]^

In the context of the group 15 elements, the stabilization of mono‐coordinate species has proliferated during the last decade. A landmark was achieved in 2012, when Bertrand and co‐workers reported on an electronically‐stabilized singlet phosphinonitrene, featuring a terminal monocoordinated nitrogen atom.^[^ ^24^ ^]^ Very recently, Beckmann and Tan independently reported on the synthesis of triplet nitrenes supported by hydrindacenyl ligands,^[^ ^25^, ^26^ ^]^ further demonstrating that careful ligand design can lead to both control of spin state and increased stability of these elusive systems (Figure 1, right). Descending group 15, the first mono‐coordinate phosphorus compound, a singlet phosphinidine, was reported by Bertrand and co‐workers.^[^ ^27^ ^]^ Attempts to access a triplet phosphinidene have been thus far unsuccessful,^[^ ^28^ ^]^ however heavier group 15 congeners have been synthesized, including mono‐coordinate triplet stibidinines and bismuthidines, all bearing extremely bulky hydrindacenyl substituents (Figure 1, right).^[^ ^29^, ^30^, ^31^ ^]^

**Figure 2:**
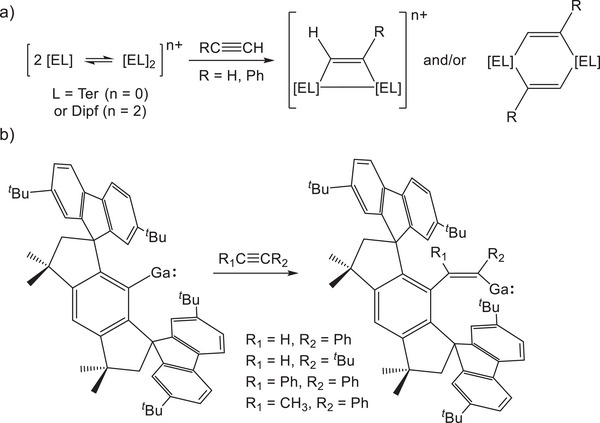
a) Reactivity of homodiatomic group 13 compounds toward alkynes; and b) reactivity of a monomeric gallium(I) compound toward alkynes.

Given the challenges in accessing such mono‐valent compounds, their reactivity remains largely unexplored. For the group 13 elements, contrasting examples of reactivity are observed depending on whether compounds exist as monomers or dimers. Power has shown that the dimeric diagallene compound (GaTer)_2_ undergoes [2 + 2] cycloadditions by reaction with alkynes (Figure 2a).^[^ ^32^ ^]^ Related cycloaddition reactions of cationic (di)gallenes have also been reported by Krossing and co‐workers.^[^ ^33^, ^34^ ^]^ Our own group reported similar reactivity for the digallene and diindene compounds (ETer)_2_ (E = Ga, In), which under controlled stoichiometric loading of acetylene produce both the neutral four and six‐membered rings 2π E_2_C_2_ and 4π E_2_C_2_.^[^ ^35^ ^]^ By contrast, Kretschmer's monomeric singly bonded gallylene reacts differently toward alkynes. Unlike the systems described above, this gallylene carbometallates the C≡C triple bond of terminal and internal alkynes (Figure 2b).^[^ ^16^ ^]^ This represents a unique example of redox invariant reactivity in a low‐valent gallium(I) compound.

These examples illustrate the ability of heavy mono‐valent group 13 species to undergo [2 + 2] cycloadditions or insertion reactions with C≡C triple bonds, however their reactivity toward compounds with C≡P remains unexplored. We were thus motivated to investigate whether phosphaalkynes, structurally related but electronically distinct analogues of alkynes, could engage in comparable reaction pathways. In this work, we report the formation of a mono‐coordinate indium(I) species through the redox‐neutral carbometallation of AdC≡P with (InTer)_2_. Also, we further explore the reversible nature of this reaction with B(C_6_F_5_)_3_ and the reactivity of the indium(I) center toward oxidants. We contrast these results with those of the lighter analogue (GaTer)_2_ which upon reaction with AdC≡P affords a dimeric P–C coupled product.

## Results and Discussion

**Scheme 1:**
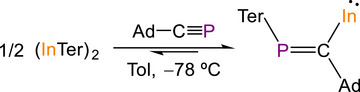
Synthesis of In[C(Ad)═PTer] (**1**).

**Figure 3:**
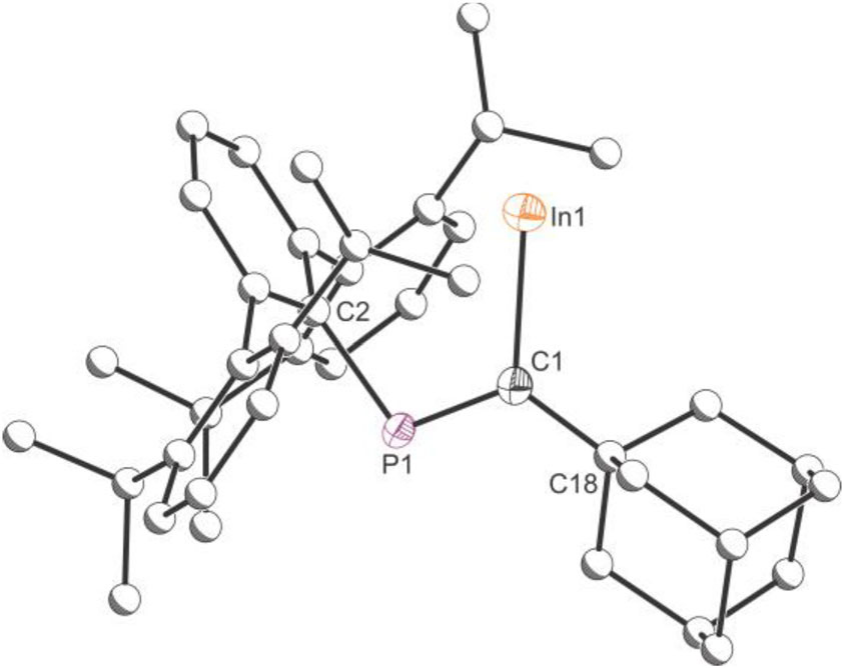
Single crystal X‐ray structure of **1**. Thermal ellipsoids set at 50% probability level; hydrogen atoms and solvent of crystallization omitted for clarity. Carbon atoms of Ad and Ter groups are depicted as spheres of arbitrary radius.

Following our studies on the reactivity of (ETer)_2_ (E = Ga, In) with acetylene, which yielded species containing four and six‐membered rings,^[^ ^35^ ^]^ we turned our attention to their reactivity with a simple phosphaalkyne. The reaction of (InTer)_2_ with two equivalents of AdC≡P at −78 °C results in an immediate color change from deep to bright red (Scheme 1). Subsequent crystallization from hexane at −35 °C results in the formation of a mixture of dark red crystals and bright red crystals, along with a black precipitate (metallic indium). The crystals were analyzed by single‐crystal X‐ray diffraction.^[^ ^36^ ^]^ The deep red crystals correspond to the starting material (InTer)_2_ whereas the bright red crystals were identified as compound In[C(Ad)═PTer] (**1**), which features a mono‐coordinate indium(I) center (Figure 3). Formation of **1** can be tentatively explained by the insertion of AdC≡P into the In─C bond of (InTer)_2_ (see below).

In situ synthesis of **1** at −78 °C in d_8_‐toluene allowed for its characterization by ^1^H and ^31^P{^1^H} NMR spectroscopy (recorded at −45 °C to avoid sample decomposition; see Supporting Information for further details, including NMR spectra recorded at room temperature). These NMR experiments reveal quantitative formation of **1** under these conditions, with no detectable signals of unreacted starting materials (Figures S3 and S4). The ^31^P{^1^H} NMR spectrum of **1** displays a singlet resonance at 304.2 ppm corresponding to the phosphaalkenyl fragment. The ^1^H NMR spectrum is consistent with a singly bonded indium(I) center; no evidence was observed for the presence of an indium(III) hydride (e.g., H_2_InC(Ad)═PTer). Further confirmation of the formation of **1** was provided by HR‐MS, which showed the expected [M + H]^+^ peak at *m*/*z* 691.2907 (calc 691.2918, error −1.56 ppm). Thermal decomposition of **1** at room temperature resulted in the formation of the starting material (InTer)_2_, an intractable mixture of phosphorous containing species (as evidenced by in situ ^1^H and ^31^P NMR spectroscopy, see Supporting Information), and a black precipitate. Attempts to synthesize compound **1** in coordinating solvents (e.g., THF, MeCN) were unsuccessful, resulting in quantitative decomposition accompanied by the formation of a black precipitate.

In the solid‐state structure of **1**, there is no evidence for aggregation of these monomeric units (d_In–In_ = 9.977 Å), in sharp contrast with (InTer)_2_ which forms a dimer in the solid state through an In─In interaction (d_In–In_ = 2.9785(5) Å).^[^ ^12^ ^]^ The In─C1 bond length in **1** is 2.279(5) Å, which is moderately elongated to that expected for an In─C single bond (Σ_cov_ (In─C) = 2.17 Å),^[^ ^37^ ^]^ and in good agreement with the monocoordinated indium species In‐C_6_H_3_‐2,6‐Trip_2_ (Trip = C_6_H_2_‐2,4,6‐*^i^* Pr_3_; In─C = 2.260(7) Å) reported by Power and coworkers in 1998.^[^ ^8^ ^]^ The P–C1 distance 1.673(5) suggests double bond character (Σ_cov_ (P═C) = 1.69 Å)^[^ ^38^ ^]^ and it is comparable to other previously reported P═C double bonds in phosphaalkene derivatives (e.g., (Ph)_2_C═P(2,6‐Mes_2_C_6_H_3_), 1.694(2) Å, Mes = 2,4,6‐trimethylphenyl).^[^ ^38^ ^]^ The carbo‐metalation of the C≡P fragment also results in the formation of a single bond between the phosphorous center and the sp^2^ carbon (C2) of the terphenyl ligand (P–C2 = 1.866(5) cf. Σ_cov_ (P–C) = 1.86 Å).^[^ ^37^ ^]^

**Figure 4:**
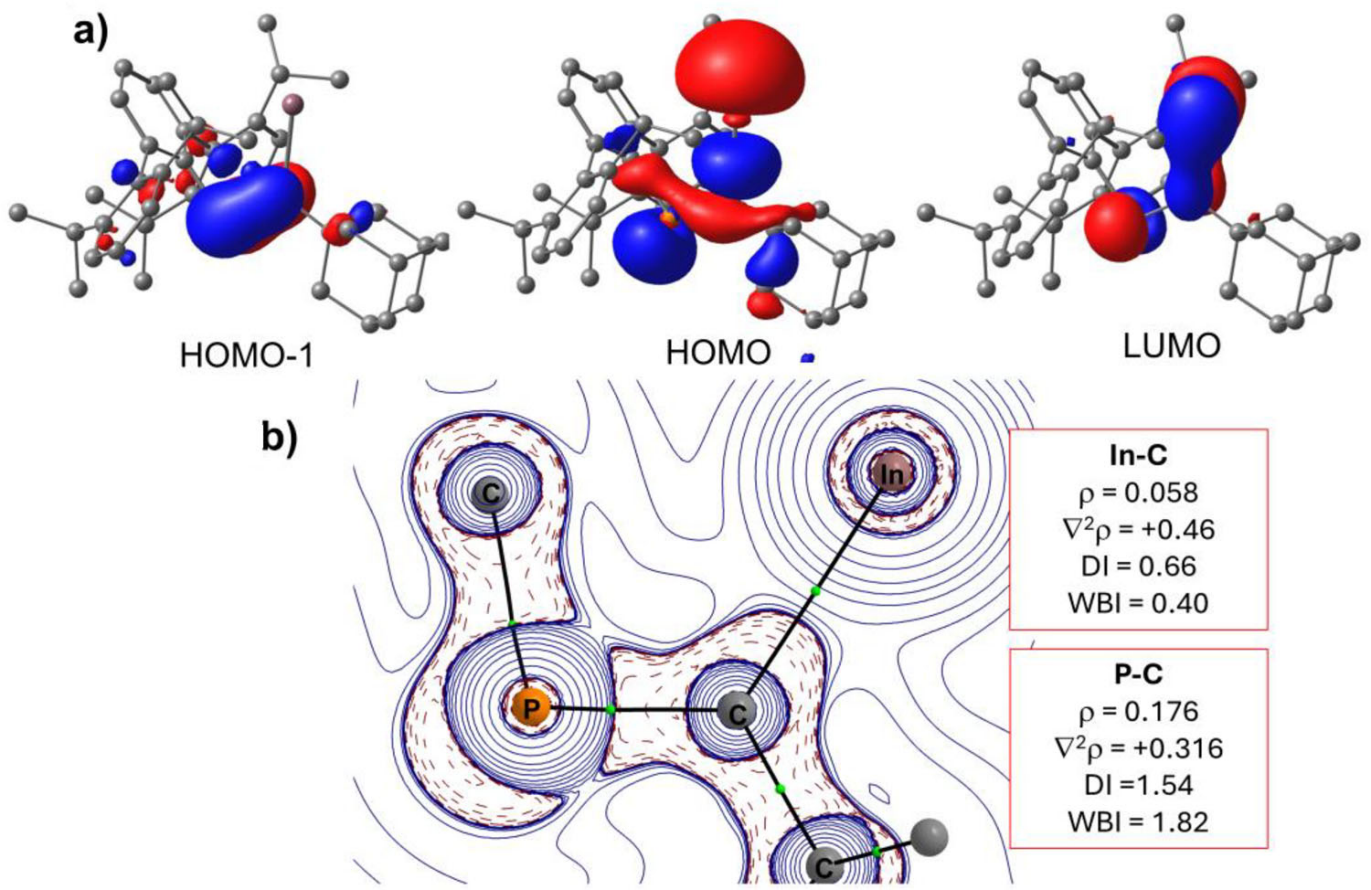
a) Frontiers orbitals of **1** (isovalue=0.03) computed at the PCM‐BP86‐D3/def2‐SVP level. Hydrogen atoms were omitted for clarity. b) Contour line diagrams of ∇^2^ρ(r) for **1** in the P−C−In plane. Solid lines connecting the atomic nuclei are bond paths, while the small green spheres indicate the corresponding bond critical points, respectively.

To better understand the electronic structure **1**, Density Functional Theory (DFT) calculations at the dispersion corrected PCM‐BP86‐D3/def2‐SVP level were performed. The highest occupied molecular orbital (HOMO) mainly consists of the indium lone pair (suggesting nucleophilic character; Figure 4a). The HOMO–1 reflects the P–C π‐bonding interaction, while the LUMO has both P–C π* and indium *p* orbital character. The computed NBO‐Wiberg Bond Indices (WBIs) for the In─C and P─C bonds are 0.40 and 1.82, respectively, which are consistent with the corresponding QTAIM‐Delocalization Indices (DIs) of 0.66 and 1.54 (Figure 4b) and Mayer bond orders of 0.74 and 1.74, respectively. These values strongly support localized single and double bonds with little electron delocalization between the C─P‐In atoms. This result is in sharp contrast with previous examples of reactions of main group carbenoids with phosphalkynes, which predominantly yield EC_2_ three‐membered rings (E = Al, Si, Ge) and/or oligomerization products.^[^ ^39^, ^40^, ^41^, ^42^, ^43^, ^44^, ^45^ ^]^ The UV–vis spectrum of in situ generated samples of **1** reveals an absorption band at λ_exp_ = 535 nm, which compares well with the value determined by Time Dependent (TD)‐DFT calculations (λ_calc_ = 492 nm, oscillator strength of 0.0159) and corresponds to the HOMO→LUMO vertical transition (see Supporting Information for further details).

**Figure 5:**
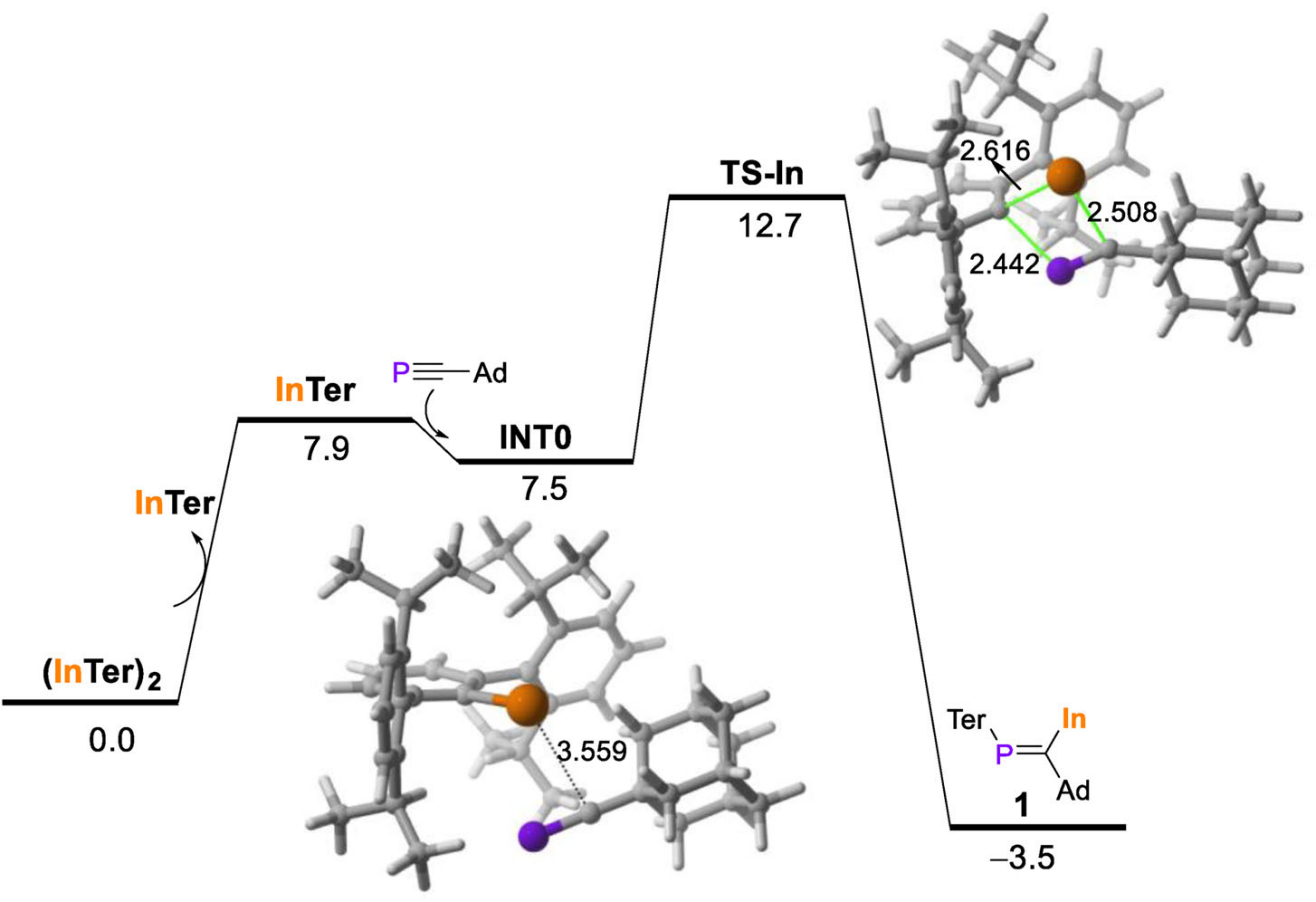
Computed reaction profile for the formation of compound **1**. Relative free energies (ΔG, at 195.15 K) and bond distances are given in kcal/mol and Å, respectively. All data have been computed at the PCM(toluene)‐BP86‐D3/def2‐TZVPP//PCM(toluene)‐BP86‐D3/def2‐SVP level.

The computed reaction profile for the formation of **1** (PCM‐BP86‐D3/def2‐TZVPP//PCM‐BP86‐D3/def2‐SVP level) is consistent with the observed experimental findings (Figure 5). The reaction involves the facile dissociation of (InTer)_2_ in solution, a process that has been experimentally determined by cryoscopy,^[^ ^12^ ^]^ and which according to our calculations is slightly endergonic (7.9 kcal/mol). This monomeric indium(I) compound interacts with AdC≡P to afford the weakly bonded van der Waals intermediate, **INT0**, that through a low barrier (ΔG^≠^ = 5.2 kcal/mol) carbometallation reaction affords compound **1**. It should be noted that the reverse process (i.e., the dissociation of **1** to afford InTer and AdC≡P) has a barrier of 16.2 kcal/mol, consistent with a reversible process, and with the observation that, on standing, solutions of **1** regenerate (InTer)_2_.

**Scheme 2:**
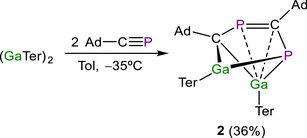
Synthesis of (GaTer)_2_(Ad‐CP)_2_ (**2**).

Intrigued by the formation of **1**, we wondered whether similar reactivity would be mirrored by the lighter group 13 analogue (GaTer)_2_. Reaction of (GaTer)_2_ with two equivalents of AdC≡P results in immediate discoloration of the reaction mixture, from deep brown to yellow (Scheme 2). In this case, the ^31^P NMR spectrum displays two doublet resonances at 201.5 and −51.6 ppm (^2^ *J*_P−P_ = 63 Hz). Compound **2** presents several broad resonances in the ^1^H and ^13^C{^1^H} NMR spectra at room temperature, indicative of restricted rotation of the Dipp groups in solution (see the Supporting Information for VT experiments).

**Figure 6:**
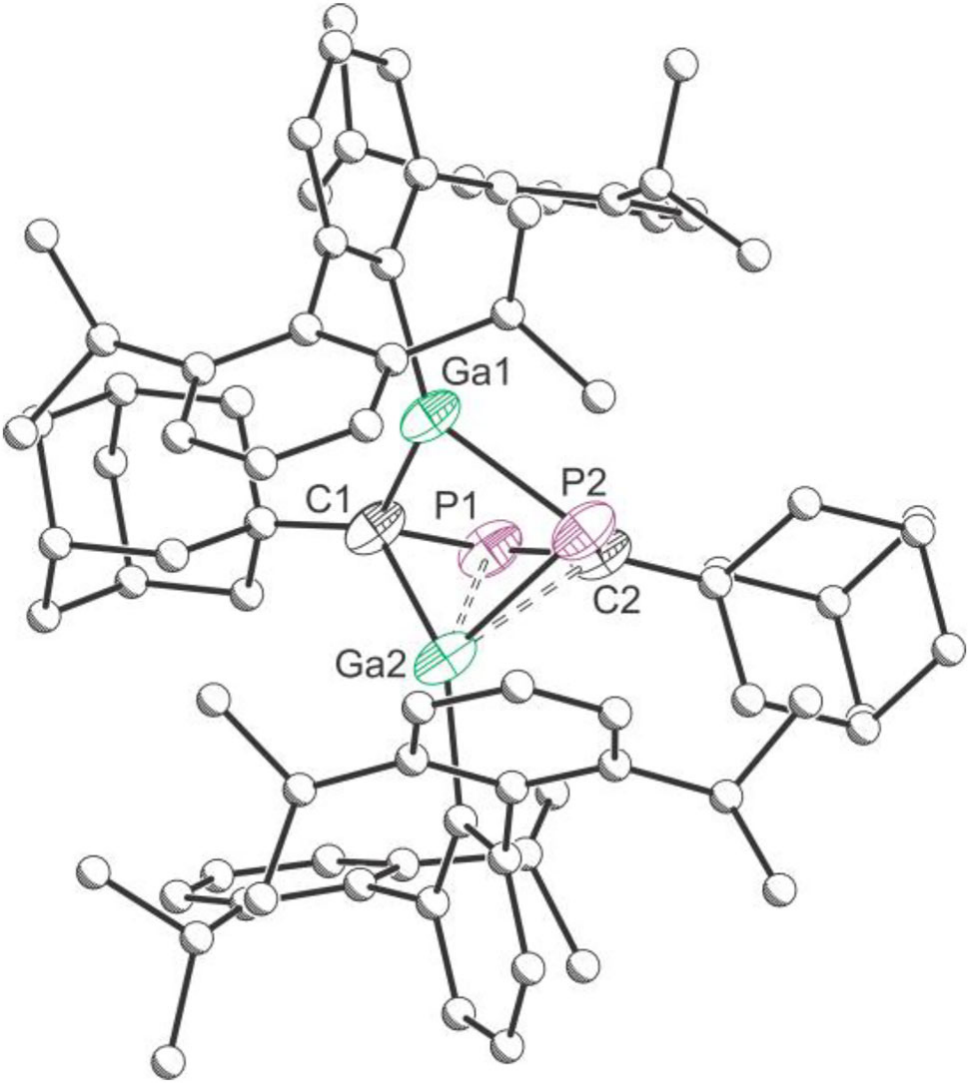
Single crystal X‐ray structure of **2**. Thermal ellipsoids set at 30% probability level; hydrogen atoms and solvent of crystallization omitted for clarity. Carbon atoms of Ad and Ter groups are depicted as spheres of arbitrary radius. Dashed bonds are used to indicate an interaction between the P1–C2 double bond and Ga2.

Compound **2** was isolated in 36% yield as a yellow powder. Single crystals suitable for X‐ray diffraction were obtained from a hexane solution at −35 °C. The solid‐state structure reveals the formation of a dimeric compound (GaTer)_2_(AdCP)_2_ (**2**) in which two adamantyl phosphaalkyne units have coupled through the formation of a C─P bond (Figure 6). The C1–P1 and C2–P2 distances are consistent with single bonds (1.840(5) and 1.859(4) cf. Σ_cov_ (P–C) = 1.86 Å; computed Mayer bond orders of 0.994 and 0.968, respectively)^[^ ^37^ ^]^ while the central P1–C2 distance lies between that a double and a single bond (1.741(5) cf. Σ_cov_ (P–C) = 1.86 Å and Σ_cov_ (P═C) = 1.67 Å; computed Mayer bond order of 1.303).^[^ ^37^, ^38^ ^]^ This elongation arises from coordination to one of the gallium(III) centers. The unusual connectivity observed in **2** resembles that of bicyclo[2.1.1]hex‐2‐ene.^[^ ^46^ ^]^ Compound **2** can also be rationalized in the context of Wade‐Mingos rules as a *nido*‐type cluster with 2*n *+ 4 cluster‐bonding electrons (and a geometry consistent with a distorted pentagonal bipyramid that is missing an apical vertex).^[^ ^47^ ^]^ This result is in stark contrast with the insertion reaction observed for the heavier counterpart (InTer)_2_.

**Figure 7:**
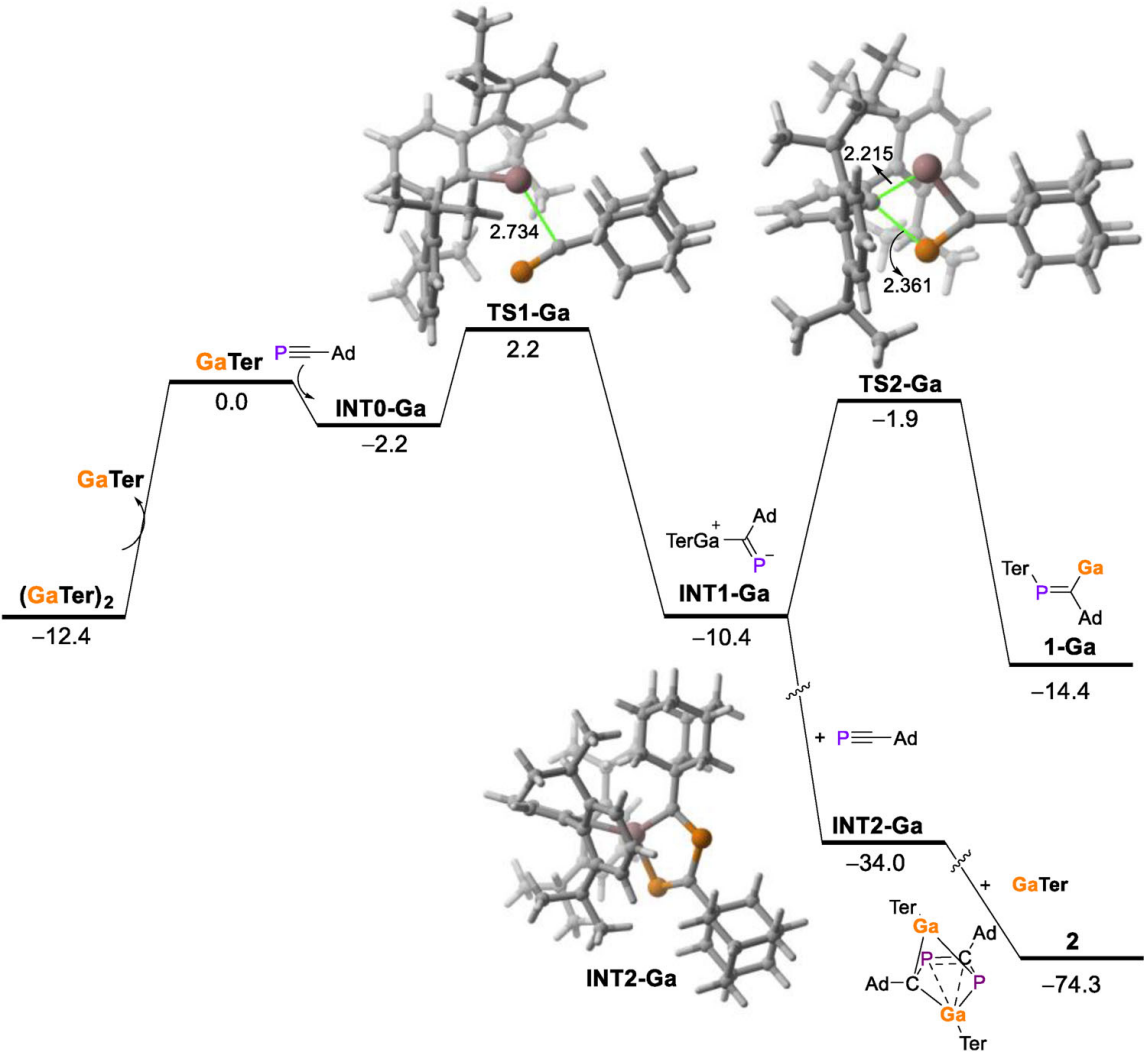
Computed reaction profile for the formation of compound **2**. Relative free energies (ΔG, at 238.15 K) and bond distances are given in kcal/mol and Å, respectively. All data have been computed at the PCM(toluene)‐BP86‐D3/def2‐TZVPP//PCM(toluene)‐BP86‐D3/def2‐SVP level.

DFT calculations were used to model the reaction profile for the formation of **2** (Figure 7). In contrast to its indium(I) counterpart, we were unable to locate a transition state on the potential energy surface similar to that of **TS‐In** which is associated with the carbometallation reaction (concerted cleavage of the In─Ter bond accompanied by the formation of the Ga–C and P–Ter bonds). Instead, the reaction between GaTer and the phosphaalkyne does not involve the Ga–Ter bond cleavage but rather the formation of a new Ga–C bond via **TS1‐Ga**, with a rather low barrier of 4.4 kcal/mol. This is very likely due to the higher Ga–C(Ter) bond strength as compared to the analogous In─C(Ter) bond, which is confirmed by the corresponding Wiberg bond indices (0.45 versus 0.38, respectively). This step leads to the exergonic formation (ΔG = −8.2 kcal/mol) of the zwitterionic species **INT1‐Ga**. This intermediate would evolve into the expected adduct **1‐Ga**, similar to the indium compound, through **TS2‐Ga** (barrier of 8.5 kcal/mol). However, addition of a new molecule of the phosphaalkyne and GaTer produces the observed species **2** in a strongly exergonic transformation (ΔG = −63.9 kcal/mol) very likely through intermediate **INT2‐Ga** (where the new C─P bond is formed). Therefore, the preferred formation of **2** over **1‐Ga** seems to result from the stronger Ga─C(Ter) bond (as compared to its indium counterpart) together with the high thermodynamic stability of the observed cluster species **2**.

**Scheme 3:**
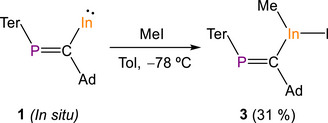
Synthesis of In(Me)I[C(Ad)═PTer] (**3**).

**Figure 8:**
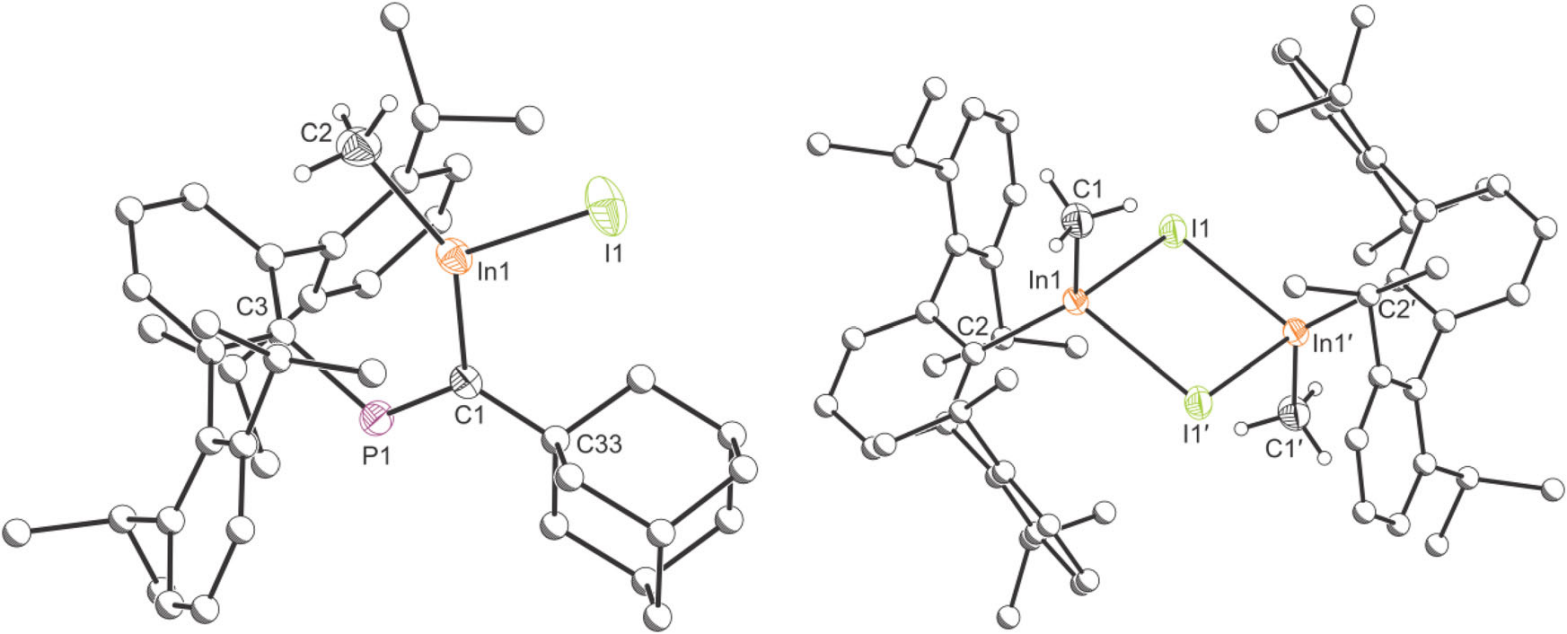
Single crystal X‐ray structure of the monomeric and dimeric structures of **3** (left) and **4** (right). Thermal ellipsoids set at 50% probability level; hydrogen atoms and solvent of crystallization, omitted for clarity. Carbon atoms of Ad and Ter groups are depicted as spheres of arbitrary radius.

To experimentally probe the oxidation state of the indium center in compound **1**, it was reacted with an equimolar amount of MeI affording In(Me)I[C(Ad)═PTer] (**3**) in 31% crystalline yield (Scheme 3). The single‐crystal X‐ray structure of **3** (Figure 8, left) exhibits a monomeric structure with a tricoordinate indium(III) center resulting from the formal oxidative addition of MeI at the indium(I) center of **1**. The ^31^P NMR spectrum of **3** reveals a singlet resonance at 328.8 ppm corresponding to the phosphaalkenyl moiety. The ^1^H and ^13^C{^1^H} NMR spectra show characteristic resonances at 0.41 and 15.25 ppm, respectively, corresponding to the indium‐bonded methyl group (see Supporting Information). It is noteworthy that compound **3** displays several broad resonances in the ^1^H and ^13^C{^1^H} NMR spectra at room temperature attributable to restricted rotation of the Dipp groups in solution (see the Supporting Information for VT experiments). Compound **3** co‐crystalizes with one methylcyclopentane molecule. Methylcyclopentane is present in hexanes solvents (≈10%), and its co‐crystalization with terphenyl‐containing molecules has previously been observed by Power and co‐workers.^[^ ^48^, ^49^, ^50^ ^]^ The In─C1 bond length in **3** is 2.156(2) Å corresponding to an In─C single bond (Σ_cov_ (In─C) = 2.17 Å)^[^ ^37^ ^]^ and the C═P bond distance is 1.679(2) Å (Σ_cov_ (P═C) = 1.69 Å).^[^ ^38^ ^]^ The In─I distance is consistent with that expected for a single bond (2.7750(2) cf. Σ_cov_ (In─I) = 2.75 Å).^[^ ^37^ ^]^ It is worth noting that the indium center in **3** presents a distorted trigonal planar geometry (C‐In‐Me = 142.05(9)°, Me‐In‐I = 99.91(7)°, I‐In‐C = 118.02(5)°, ∑° = 360°).

**Scheme 4:**
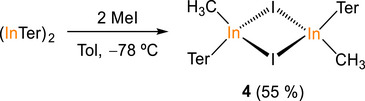
Synthesis of {TerInMe(μ‐I)}_2_ (**4**).

The surprising monomeric nature of **3** contrasts sharply with previously reported indium halides bearing terphenyl substituents, which are prone to form dimeric structures via halogen bridges (e.g., {2,6‐Trip_2_H_3_C_6_InCl(μ‐Cl)}_2_ (Trip = 2,4,6‐triisopropylphenyl) or {2,6‐Mes_2_C_6_H_3_InCl(μ‐Cl)}_2_ (Mes = 2,4,6‐trimethylphenyl).^[^ ^51^, ^52^, ^53^ ^]^ To assess how the steric protection offered by the phosphaalkenyl substituent present in **1** contrasts with that of (InTer)_2_, the latter compound was also reacted with MeI. This reaction affords the bimetallic compound {TerInMe(μ‐I)}_2_ (**4**) in 55% yield (Scheme 4). Compound **4** has a dimeric structure in the solid state as a result of the formation of iodine bridges between the indium centers which give rise to a In_2_(μ‐I)_2_ ring (Figure 8, right). The inter‐ring In─I distances (2.8633(5) and 2.8868(5) Å) are longer than the value expected for an In─I single bond (Σ_cov_ (In─I) = 2.75 Å) and slightly shorter than previous examples of In_2_(μ‐I)_2_ four‐membered rings (e.g., [Mes_2_In(μ‐I)]_2_, 2.9009(6) and 2.9885(5) Å; {[Mn(CO)_5_]_2_In(μ‐I)}_2_, 2.9501(6) and 2.952(1) Å).^[^ ^54^, ^55^ ^]^ The indium atoms exhibit a distorted tetrahedral geometry as the bond angles about the indium range from 89.54 to 124.83°. The difference between the aggregation states in **3** and **4** (monomer versus dimer) confirms the overall increase of steric protection offered to the indium center by the [C(Ad)═PTer] ligand, which prevents dimerization.

**Scheme 5:**
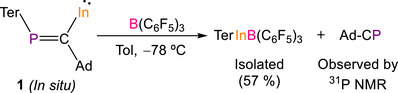
Reaction of **1** with B(C_6_F_5_)_3_.

In an effort to stabilize the highly reactive compound **1** by the formation of an adduct with a Lewis acid, we explored its reactivity toward B(C_6_F_5_)_3_ (Scheme 5). Treatment of **1** with B(C_6_F_5_)_3_ in toluene at −78 °C resulted in the unexpected formation of [TerInB(C_6_F_5_)_3_] in 57% crystalline yield. This compound was previously reported by Power as the adduct formed between (InTer)_2_ and B(C_6_F_5_)_3_.^[^ ^12^ ^]^ To obtain more insights into the mechanism by which [TerInB(C_6_F_5_)_3_] is formed, the reaction of **1** with B(C_6_F_5_)_3_ was monitored in situ by ^1^H, ^31^P and ^19^F{^1^H} NMR spectroscopy at room temperature. Upon addition of B(C_6_F_5_)_3_, compound **1** was quantitatively consumed, resulting in the formation of [TerInB(C_6_F_5_)_3_] and AdC≡P (see Supporting Information for heteronuclear NMR spectra and low temperature UV–vis experiments).

**Scheme 6:**
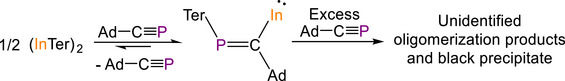
Proposed decomposition pathway for **1**.

The thermal decomposition of **1** at room temperature to (InTer)_2_, and the formation of [TerInB(C_6_F_5_)_3_] and AdC≡P upon treatment of **1** with B(C_6_F_5_)_3_, indicate that the reaction between (InTer)_2_, and AdC≡P may be reversible, in agreement with DFT calculations. Despite the complex mixture resulting from the thermal decomposition of **1**, these experiments suggest that one possible thermal decomposition pathway for **1** could potentially be the further reaction of **1** with AdC≡P resulting in uncontrolled oligomerization (Scheme 6). To explore this hypothesis, (InTer)_2_ was treated with four equivalents of AdC≡P resulting in the complete consumption of AdC≡P, along with the formation of **1** and several unidentified phosphorous‐containing species as evidenced by ^31^P NMR spectroscopy (see Supporting Information). Reaction of (InTer)_2_ with a large excess of AdC≡P (10 equivalents) results in the complete consumption of **1**. In this case an excess of AdC≡P is still present in the reaction mixture along with unidentified phosphorous‐containing species. Due to the complex mixtures obtained in these reactions, none of these species could be identified. Overall, these experiments point to a reversible reactivity pattern in which **1** undergoes further reaction with AdC≡P, ultimately leading to uncontrolled oligomerization products.

## Conclusion

The synthesis of compounds containing monocoordinated heavy main group elements remains challenging not only for their intrinsic instability but also for the absence of a general synthetic strategy. Here we have shown that the reaction of (InTer)_2_ with AdC≡P affords a monocoordinate indium(I) phosphaalkenyl, In[C(Ad)═PTer]. The gallium analogue (GaTer)_2_, by contrast, reacts differently resulting in the dimerization of the AdC≡P fragments to form (GaTer)_2_(AdCP)_2_. Furthermore, reaction of In[C(Ad)═PTer] with B(C_6_F_5_)_3_ results in the formation of [TerInB(C_6_F_5_)_3_] and AdC≡P, providing evidence of the reversible nature of the insertion process. These findings highlight the distinct reactivity pattern of these gallium and indium low valent species toward AdC≡P resulting in highly unusual bonding motifs.

## Supporting Information

The authors have cited additional references within the Supporting Information.^[^ ^56^, ^57^, ^58^, ^59^, ^60^, ^61^, ^62^, ^63^, ^64^, ^65^, ^66^, ^67^, ^68^, ^69^, ^70^, ^71^, ^72^, ^73^, ^74^, ^75^, ^76^, ^77^ ^]^

## Conflict of Interests

The authors declare no conflict of interest.

## Supporting information



Supporting Information

Supporting Information

## Data Availability

The data that support the findings of this study are available in the Supporting Information of this article.
