# Supplementary material for: A Crystalline Mono‐Coordinate Indium(I)‐Phosphaalkenyl
Source: Angew Chem Int Ed Engl. 2026 Jan 15;65(8):e23125. doi: 10.1002/anie.202523125 (PMC12910146; doi:10.1002/anie.202523125)
Supplement: Supplementary file 1 — Supporting Information [file ANIE-65-e23125-s001.docx]

Contents

[1. Experimental Details 2](#_Toc218778410)

[2. NMR spectra 7](#_Toc218778411)

[2.1 NMR spectra of compound **1** 7](#_Toc218778412)

[2.2 NMR spectra of compound **2** 11](#_Toc218778413)

[2.3 NMR spectra of compound **3** 14](#_Toc218778414)

[2.4 NMR spectra of compound **4** 17](#_Toc218778415)

[3. VT-NMR experiments 19](#_Toc218778416)

[4. *In situ* NMR experiments 21](#_Toc218778417)

[5. High resolution mass data 26](#_Toc218778418)

[6. In situ UV-Vis experiments 28](#_Toc218778419)

[7. X-ray crystallographic studies 30](#_Toc218778420)

[8. Computational details 39](#_Toc218778421)

[9. References 68](#_Toc218778422)

# Experimental Details

*General Synthetic Methods.* All reactions and product manipulations were carried out using standard Schlenk-line techniques under an inert atmosphere of argon, or in a dinitrogen filled glovebox (MBraun UNIlab glovebox maintained at <0.1 ppm H_2_O and <0.1 ppm O_2_). (GaTer)_2_ and (InTer)_2_ were synthesized according to previously reported synthetic procedures.^[11,12]^ Benzene (anhydrous, Sigma Aldrich), toluene (Fisher Chemical HPLC grade), hexane (Fisher Chemical HPLC grade) and pentane (Fisher Chemical HPLC grade) were purified using a Pure Process Technology (PPT) solvent purification system (SPS). C_6_D_6_ (Aldrich, 99.5%) and d_8_-toluene (Aldrich, 99%) were distilled over sodium/benzophenone. All dry solvents were stored under argon in gas-tight ampoules over activated 3 Å molecular sieves.

*Characterization techniques.* NMR spectra were acquired on a Bruker 500 MHz Avance Neo, a Varian 500 MHz Inova, or a Varian 400 MHz Inova NMR spectrometer. Chemical shifts (δ) are reported in parts per million (ppm). ^1^H and ^13^C NMR spectra are referenced to TMS using the most downfield protio-solvent resonance (^1^H NMR C_6_D_6_: δ = 7.16 ppm, ^13^C NMR C_6_D_6_: δ = 128.06 ppm; ^1^H NMR d_8_-toluene: δ = 7.09 ppm). ^31^P NMR spectra are externally referenced to an 85% solution of H_3_PO_4_ in H_2_O. See Supporting Information for a full assignment of NMR resonances. High-resolution mass spectra were recorded on a Thermo Q-Exactive Plus (APCI-Orbitrap, positive ion mode) instrument at the Mass Spectrometry Facility of the Department of Chemistry of Indiana University. UV–visible spectra were recorded on an Agilent Cary 60 UV–visible spectrometer. X-ray diffraction data were collected with a Bruker D8 Venture diffractometer equipped with a PhotonII detector and IμS sources, using various experiment temperatures, collection strategies, and exposure times. Details are summarized in the CIF files and the Supporting Information.

*In situ synthesis of In[C(Ad)=PTer]* *(****1****)*. To a solution of (InTer)_2_ (40 mg, 0.039 mmol) in toluene (3 ml) at –78 °C, a solution of AdCP (13.9 mg, 0.078 mmol, 2 eq) in toluene (3 ml) was added dropwise. The solution immediately lightened in color from deep red to bright red. Compound **1** readily decomposes at room temperature (see Supporting Information). The *in situ* ^1^H and ^31^P NMR spectra of **1** in d_8_-toluene were recorded at –45 °C. ^1^H NMR (228K, 500 MHz, tol-d_8_): δ (ppm) 7.24 (t, ^3^*J*_H–H_ = 7.8 Hz, 2H; *para*-Dipp), 7.18–7.10 (m, 7H; *para*-Ter, *meta*-Ter, *meta*-Dipp), 3.33 (br, 4H; C*H*(CH_3_)_2_), 1.75 (br, 3H; C*H*-Ad), 1.50 (br, 18H; C*H*_2_-Ad and CH(C*H*_3_)_2_), 1.42 (d, *^2^J*_H–H_ = 12.9 Hz , 3H; C*H*_2_-Ad), 1.25 (d, ^2^*J*_H–H_ = 12.9 Hz , 3H; C*H*_2_-Ad), 1.15 (br, 12H; CH(C*H*_3_)_2_). ^31^P{^1^H} NMR (228K, 202 MHz, tol-d_8_): δ (ppm) 304.2 8 (s). ^1^H NMR (298 K, 500 MHz, C_6_D_6_): δ (ppm) 7.30-7.16 (m, 9H; *para*-Dipp, *para*-Ter, *meta*-Ter, *meta*-Dipp), 3.33 (sept, ^2^*J*_H–H_ = 6.9 Hz, 4H; C*H*(CH_3_)_2_), 1.77 (br, 3H; C*H*-Ad), 1.53 (br, 6H; C*H_2_*-Ad), 1.49 (d, ^3^*J*_H–H_ = 6.9 Hz, 12H; CH(C*H*_3_)_2_), 1.44 (d, ^2^*J*_H–H_ = 12.9 Hz , 3H; CH_2_-Ad), 1.32 (d, ^2^*J*_H–H_ = 12.9 Hz; 3H, CH_2_-Ad, 1.14 (d, ^3^*J*_H–H_ = 6.9 Hz, 12H; CH(C*H*_3_)_2_). Incipient decomposition prevented reliable integration of several resonances. ^31^P{^1^H} NMR (298 K, 202 MHz, C_6_D_6_): δ (ppm) 301.1 (s). ^31^P NMR (298 K, 202 MHz, C_6_D_6_): δ (ppm) 301.1 (s). HR-MS [APCI-Orbitrap, positive ion mode]: *m*/*z* for C_41_H_53_InP [**1**+H]^+^ Calcd: 691.2918, Found: 691.2907 (–1.56 ppm error).

*Synthesis of (GaTer)_2_(AdCP)_2_ (****2****)*. To a solution of (GaTer)_2_ (35 mg, 0.037 mmol) in toluene (3 ml) at –35 °C a solution of AdCP (13.3 mg, 0.074 mmol, 2 eq) in toluene (3 ml) was added dropwise. The resulting orange solution was allowed to reach room temperature before all volatiles were removed under a dynamic vacuum, yielding an orange oil. This oil was redissolved in hexane (0.5 ml) and stored at –35 °C overnight. The resulting yellow solid was washed with hexane (3 × 0.3 ml) at –35 °C affording **2** as a yellow powder. Yield 17.3 mg (0.013 mmol, 36%). Single crystals of **2** suitable for single crystal X-ray diffraction were obtained by slow evaporation from a concentrated hexane solution of the product at –35 °C. ^1^H NMR (500 MHz, C_6_D_6_): δ (ppm) 7.51–6.95 (br, overlapped with benzene resonance, 18H; *meta*-Ter, *para*-Ter, *meta*-Dipp, *para*-Dipp), 3.45–2.31 (br, 8H; C*H*(CH_3_)_2_), 2.10 (br, 3H; C*H*-Ad), 1.92 (br, 6H; C*H*_2_-Ad), 1.88 (d, ^2^*J*_H–H_ = 12.10 Hz, 3H; C*H*_2_-Ad), 1.81 (br, 3H; C*H*-Ad), 1.78 (d, ^2^*J*_H–H_ = 12.10 Hz, 3H; C*H*_2_-Ad), 1.61 and 1.56 (two d, ^2^*J*_H–H_ = 12.80 Hz , 6H; C*H*_2_-Ad), 1.52–1.25 (br, 24H; CH(C*H*_3_)_2_), 1.23 (br, 6H; C*H*_2_-Ad), 1.12–0.97 (br, 24H; CH(C*H*_3_)_2_). Several broad resonances could not be reliably integrated (see VT experiments). ^13^C{^1^H} NMR (125 MHz, C_6_D_6_): δ (ppm) 149.61 (br; *ipso*-Ter), 147.37 (br; *ortho*-Dipp), 146.82 (br; *ortho*-Ter) 142.29 (s; *ipso*-Dipp), 131.02 (br; *meta*-Ter), 129.34 (br; *para*-Dipp), 126.85 (s; *para*-Ter), 124.07 (br; *meta*-Dipp), 47.96 (d, ^3^*J*_C–P_ = 8.7 Hz; *C*H_2_-Ad), 46.18 (t, ^2^*J*_C–P_ = 13.5 Hz; *C*(CH_2_)_3_-Ad), 44.36 (br; *C*H_2_-Ad), 38.54 (d, ^2^*J*_C–P_ = 12.1 Hz; *C*(CH_2_)_3_-Ad), 37.35 (s; *C*H_2_-Ad), 36.24 (s; *C*H_2_-Ad), 30.84 (s; *C*H(CH_3_)_2_), 30.00 and 29.88 (two s; *C*H-Ad), 26.87, 26.62 and 24.36 (br; CH(*C*H_3_)_2_). Phosphorous-bonded carbon signals were not detected despite employing a broad chemical shift window and an extended acquisition time. ^31^P{^1^H} NMR (202 MHz, C_6_D_6_): δ (ppm) 202.1 (d, ^2^*J*_P–P_ = 63 Hz), 51.6 (d, ^2^*J*_P–P_ = 63 Hz). ^31^P NMR (202 MHz, C_6_D_6_): δ (ppm) 202.1 (d, ^2^*J*_P–P_ = 63 Hz), 51.6 (d, ^2^*J*_P–P_ = 63 Hz). HR-MS [APCI-Orbitrap, positive ion mode]: not observed.

*Synthesis of In(Me)I[(Ad)C=PTer] (****3****)*. To a solution of (InTer)_2_ (40 mg, 0.039 mmol) in toluene (3 ml) at –78 °C, a solution of AdCP (13.9 mg, 0.078 mmol, 2 eq) in toluene (3 ml) was added dropwise yielding a bright red solution. After 10 mins, a solution of MeI in toluene (0.078 mmol, 2.35 ml, 33 mM toluene stock solution) was added dropwise at –78 °C. After 10 mins stirring at –78 °C, the resulting pale orange solution was allowed to reach room temperature. All the volatiles were removed under vacuum. The resulting residue was extracted with 0.5 ml of hexane and storage at –35 °C to yield **3** as colorless crystals, which were dried under dynamic vacuum. Yield: 20.3 mg (0.024 mmol, 31 %). ^1^H NMR (500 MHz, C_6_D_6_): δ (ppm) 7.21 (t, ^3^*J*_H–H_ = 7.8 Hz, 2H; *para*-Dipp), 7.12–7.02 (br, 4H; *meta*-Dipp, overlapped with *meta*-Ter), 7.06 (d, 2H, ^3^*J*_H–H_ = 7.6 Hz, *meta*-Ter, overlapped with *meta*-Dipp), 6.69 (d, ^3^*J*_H–H_ = 7.6 Hz, 1H, *para*-Ter), 3.15–2.73 (br, 4H; C*H*(CH_3_)_2_), 1.96 (br, 6H; C*H*-Ad), 1.84 (br, 3H; C*H*_2_-Ad), 1.48 (d, ^2^*J*_H–H_ = 12.10 Hz , 3H; C*H*_2_-Ad), 1.43–1.31 (m, 15H; CH(C*H*_3_)_2_ and C*H*_2_-Ad), 1.14–0.86 (br, 12H; CH(C*H*_3_)_2_), 0.41 (s, 3H; InC*H*_3_). ^13^C{^1^H} NMR (125 MHz, C_6_D_6_): δ (ppm) 222.75 (d, ^1^*J*_C–P_ = 70.5; *C*=P), 146.72 (br; *ortho*-Dipp), 145.46 (s; *ortho*-Ter), 144,38 (d, ^2^J_C-P_ = 45.5 Hz; *ipso*-Ter) 138.06 (s; *ipso*-Dipp) 130.77 (s; *meta*-Ter), 129.61 (s; *para*-Dipp) 129.26 (s; *para*-Ter), 123.54 (br; *meta*-Dipp), 47.10 (d, ^2^*J*_C–P_ = 13.7 Hz; *C*(CH_2_)_3_-Ad), 44.93 (d, ^3^*J*_C–P_ = 20.1 Hz; *C*H_2_-Ad), 36.02 (s; *C*H_2_-Ad), 31.72 (s; *C*H(CH_3_)_2_), 29.36 (s; *C*H-Ad), 25.67 and 23.35 (s; CH(*C*H_3_)_2_), 15.25 (s; In*C*H_3_). ^31^P{^1^H} NMR (202 MHz, C_6_D_6_): δ (ppm) 328.8 (s). ^31^P NMR (202 MHz, C_6_D_6_): δ (ppm) 328.8 (s). HR-MS [APCI-Orbitrap, positive ion mode]: *m*/*z* for C_42_H_56_InPI [**3**+H]^+^ Calcd: 833.2197, Found: 83.2186 (–1.42 ppm error).

*Synthesis of {TerInMe(μ-I)}_2_ (****4****)*. To a solution of (InTer)_2_ (30 mg, 0.029 mmol) in toluene (3 ml) at –78 °C, a solution of MeI (0.058 mmol, 1.75 ml, 33 mM toluene stock solution, 2 eq) was added dropwise at –78 °C. The solution immediately lightened in color, from deep red to a colorless solution. After 10 mins, the resulting colorless solution was allowed to reach room temperature. All the volatiles were removed under vacuum. The resulting colorless solid was washed with hexane (3 × 0.2 ml) at −35 °C to yield **4** as a colorless powder. Yield 21.2 mg (0.016 mmol, 55%). Single crystals of **4** suitable for single crystal X-ray diffraction were obtained from a concentrated hexane solution of the product at −35 °C. ^1^H NMR (500 MHz, C_6_D_6_): δ (ppm) 7.30–7.19 (m, 10H; *meta*-Dipp, *para*-Dipp, *para*-Ter), 7.14 (d, ^3^*J*_H–H_ = 7.9 Hz, 8H; *meta*-Ter), 3.01 (sept, ^3^*J*_H–H_ = 6.9 Hz, 8H; C*H*(CH_3_)_2_), 1.30 (d, ^3^*J*_H–H_ = 6.9 Hz, 24H; CH(C*H*_3_)_2_), 1.02 (d, ^3^*J*_H–H_ = 6.9 Hz, 24H; CH(C*H*_3_)_2_), 0.11 (s, 6H; InC*H*_3_). ^13^C{^1^H} NMR (125 MHz, C_6_D_6_): δ (ppm) 159.83 (s; *ipso*-Ter), 146.98 (s; *ortho*-Dipp), 146.69 (s; *ortho*-Ter) 141.44 (s; *ipso*-Dipp), 129.60 (s; *para*-Dipp), 128.54 (s; *meta*-Ter), 128 (s; *para*-Ter, overlapped with benzene resonance), 124.14 (s; *meta*-Dipp), 31.71 (s; *C*H(CH_3_)_2_), 26.15 and 23.20 (s; CH(*C*H_3_)_2_), 8.68 (s; In*C*H_3_). HR-MS [APCI-Orbitrap, positive ion mode]: *m*/*z* for C_62_H_80_IIn_2_ [**4**-I]^+^ Calcd: 1181.3376. Found: 1181.3368 (–0.78 ppm error).

*Synthesis of [TerInB(C_6_F_5_)_3_] from* ***1***. To a solution of (InTer)_2_ (50 mg, 0.049 mmol) in toluene (3 ml) at –78 °C, a solution of AdCP (17.4 mg, 0.098 mmol, 2 eq) in toluene (3 ml) was added dropwise affording a bright red solution of 1. After 10 minutes, a solution of B(C_6_F_5_)_3_ (49.9 mg, 0.098 mmol) in toluene (3 ml) was added dropwise. The solution immediately lightened in color, from bright red to pale-yellow. After 10 minutes, the resulting solution was allowed to reach room temperature. All the volatiles were removed under vacuum. The resulting residue was extracted with 5 ml of hexane, concentrated to an approx. volume of 3 ml, and stored at –35 °C to yield [TerInB(C_6_F_5_)_3_] as colorless crystals suitable for X-ray diffraction. Yield: 57.3 mg (0.056 mmol, 57 %). Analytical data are consistent with literature reported values.^[12]^ ^1^H NMR (400 MHz, C_6_D_6_): δ (ppm) 7.18 (m, 3H; *para*-Dipp, *para*-Ter, overlapped with benzene resonance), 7.10 (m, 2H; *meta*-Ter), 6.97 (d, ^3^*J*_H–H_ = 8.0 Hz, 4H; *meta*-Dipp), 2.77 (sept, ^3^*J*_H–H_ = 6.9 Hz, 4H; C*H*(CH_3_)_2_), 1.06 (d, ^3^*J*_H–H_ = 6.9 Hz, 12H CH(C*H*_3_)_2_), 0.91 (d, ^3^*J*_H–H_ = 6.9 Hz, 12H; CH(C*H*_3_)_2_). ^19^F{^1^H} NMR (376 MHz, C_6_D_6_): δ (ppm) –128.15 (dd, ^3^*J*_F–F_ = 25.0 Hz and ^4^*J*_F–F_ = 6.6 Hz, 6F; *ortho*-C_6_F_5_), –154.50 (t, ^3^*J*_F–F_ = 25.0 Hz, 3F; *para*-C_6_F_5_), –159.56 (dt, ^3^*J*_F–F_ = 25.0 Hz and ^4^*J*_F–F_ = 6.6 Hz, 6F; *meta*-C_6_F_5_).

# NMR spectra

## 2.1 NMR spectra of compound **1**

**Figure S1.** Compound **1**, In[C(Ad)=PTer], with the atom labelling used for the assignment of NMR spectra (below).

Synthesis and characterization of **1** in tol-d_8_ at low temperature:

Compound **1** was synthetized at –78 ºC on an NMR scale in the glovebox by the addition of a solution of AdCP (0.011 mmol, 2 mg) in tol-d_8_ (≈ 0.3 ml) to a solution of (InTer)_2_ (0.0058 mmol, 6 mg) in tol-d_8_ (≈ 0.3 ml). The mixture was immediately transferred to a J. Young NMR tube and frozen by immersion in liquid nitrogen. ^1^H and ^31^P{^1^H} NMR spectra were immediately recorded in a precooled spectrometer at 228 K.

**^1^H NMR (228 K, 500 MHz, tol-d_8_):** δ (ppm) 7.24 (t, ^3^*J*_H–H_ = 7.8 Hz, 2H; H_8_), 7.18–7.10 (m, 7H; H_3_ + H_4_ + H_7_), 3.33 (br, 4H; H_9_), 1.75 (br, 3H; H_15_), 1.50 (br, 18H; H_11_ or H_12_ + H_14_), 1.42 (d, ^2^*J*_H–H_ = 12.9 Hz; 3H, H_17 or 18_), 1.25 (d, ^2^*J*_H–H_ = 12.9 Hz; 3H, H_17_ or H_18_), 1.15 (br, 12H; H_11_ or H_12_).

**^31^P{^1^H} NMR (228 K, 202 MHz, tol-d_8_):** δ (ppm) 304.2 (s).

Synthesis and characterization of **1** in C_6_D_6_ at room temperature:

Compound **1** was synthetized on an NMR scale by the addition of a solution of Ad-CP (0.0058 mmol, 1 mg) in C_6_D_6_ (≈ 0.3 ml) to a solution of (InTer)_2_ (0.0029 mmol, 3 mg) in C_6_D_6_ (≈ 0.3 ml). The ^1^H and ^31^P{1H} were immediately recorded at room temperature. Incipient decomposition prevented reliable integration of several resonances.

**^1^H NMR (298 K, 500 MHz, C_6_D_6_):** δ (ppm) 7.30–7.16 (m, 9H; H_3_ + H_4_ + H_7_ + H_8_), 3.33 (sept, ^2^*J*_H–H_ = 6.9 Hz, 4H; H_9_), 1.77 (br, 3H; H_15_), 1.53 (br, 6H; H_14_), 1.49 (d, ^3^*J*_H–H_ = 6.9 Hz, 12H; H_10_ or H_11_), 1.44 (d, ^2^*J*_H–H_ = 12.9 Hz; 3H, H_17 or 18_), 1.32 (d, ^2^*J*_H–H_ = 12.9 Hz; 3H, H_17 or 18_), 1.14 (d, ^3^*J*_H–H_ = 6.9 Hz, 12H; H_10_ or H_11_). Incipient decomposition prevented reliable integration of several resonances.

**^31^P{^1^H} NMR (298 K, 202 MHz, C_6_D_6_):** δ (ppm) 301.1 (s).

**^31^P NMR (298 K, 202 MHz, C_6_D_6_):** δ (ppm) 301.1 (s).


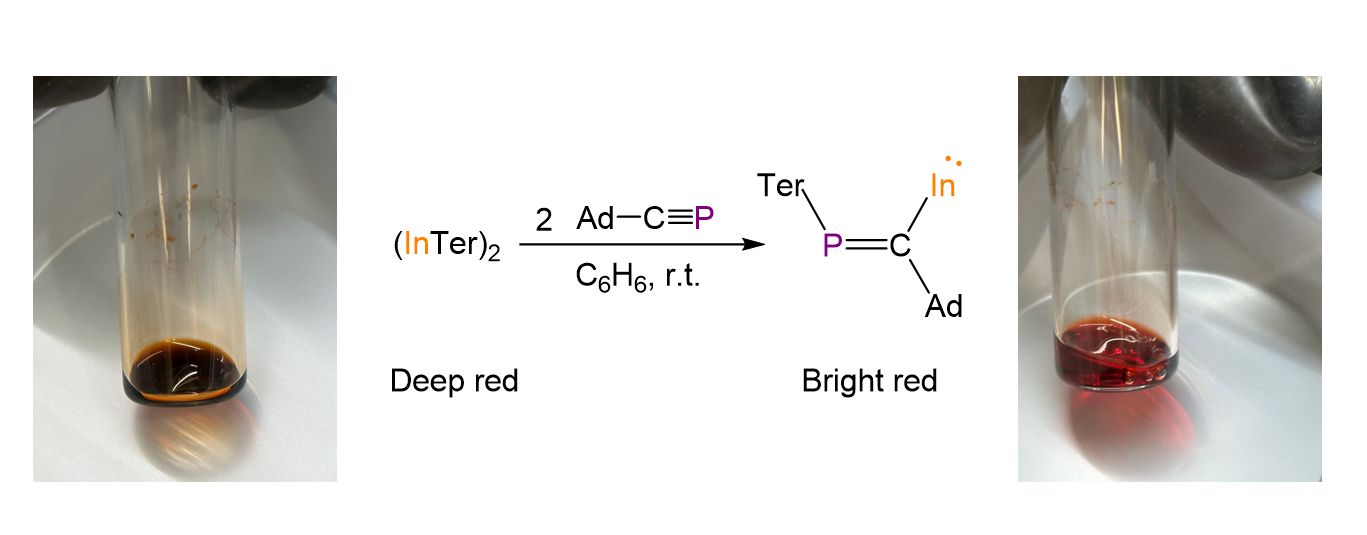


**Figure S2.** Images of the solution of (InTer)_2_ in C_6_H_6_ before (deep red) and after treatment with 2 equivalents of AdCP (bright red) at room temperature.


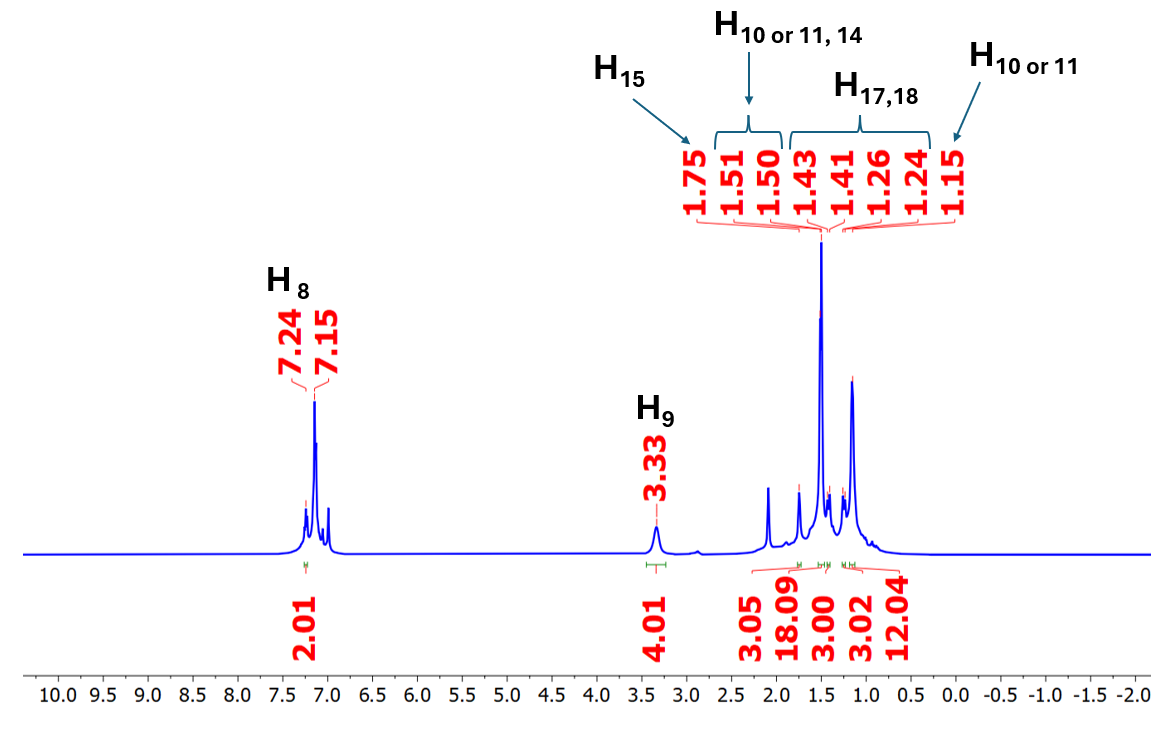


**Figure S3.** In situ ^1^H NMR spectrum (228 K, tol-d_8_, 500 MHz) of **1** synthetized at –78ºC.


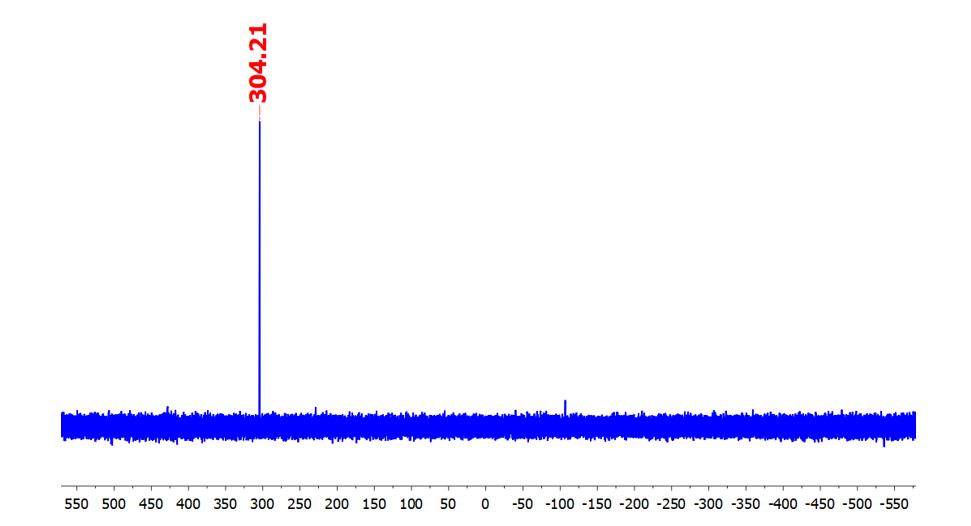


**Figure S4.** In situ ^31^P{^1^H} NMR spectrum (228 K, tol-d_8_, 202 MHz) of **1** synthetized at –78ºC.


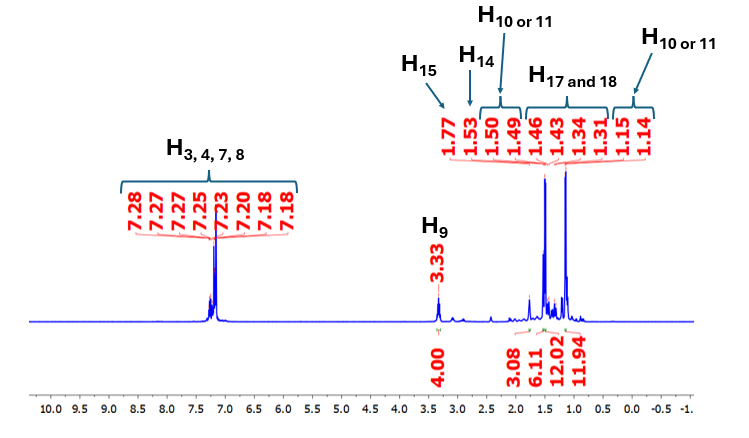


**Figure S5.** In situ ^1^H NMR spectrum (298 K, C_6_D_6_, 500 MHz) of freshly synthetized **1** at room temperature. Incipient decomposition is observed even after a short period of time.


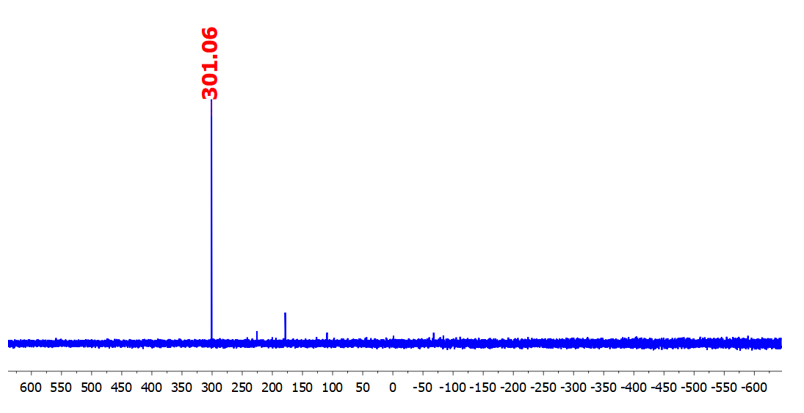


**Figure S6.** In situ ^31^P{^1^H} NMR spectrum (298 K, C_6_D_6_, 202 MHz) of freshly synthetized **1** at room temperature. Incipient decomposition is observed even after a short period of time.

## 2.2 NMR spectra of compound **2**

**Figure S7.** Compound **2**, (GaTer)_2_(AdCP)_2_, with the atom labelling used for the assignment of NMR spectra.

**^1^H NMR (500 MHz, C_6_D_6_):** δ (ppm) 7.51-6.95 (br, overlapped with benzene resonance, 18H; H_5_ + H_6_ + H_9_ + H_10_), 3.45-2.31 (br, 8H; H_11_), 2.10 (br, 3H; H_16_ or H_22_), 1.92 (br, 6H; H_15_), 1.88 (d, ^2^*J*_H–H_ = 12.10 Hz , 3H; H_18 or 19_), 1.81 (br, 3H; H_16_ or H_22_), 1.78 (d, ^2^*J*_H–H_ = 12.10 Hz , 3H; H_18 or 19_), 1.61 and 1.56 (two d, ^2^*J*_H–H_ = 12.80 Hz , 6H; H_24_ + H_25_), 1.52–1.25 (br, 24H; H_12_ or H_13_), 1.23 (br, 6H; H_21_), 1.12–0.97 (br, 24H; H_12_ or H_13_). Several broad resonances could not be reliably integrated (see VT experiments).

**^13^C{^1^H} NMR (125 MHz, C_6_D_6_):** δ (ppm) 149.61 (br; C_3_), 147.37 (br; C_8_), 146.82 (br; C_4_) 142.29 (s; C_7_), 131.02 (br; C_5_), 129.34 (br; C_10_), 126.85 (s; C_6_), 124.07 (br; C_9_), 47.96 (d, ^3^*J*_C–P_ = 8.7 Hz; C_15_), 46.18 (t, ^2^*J*_C–P_ = 13.5 Hz; C_20_), 44.36 (br; C_21_), 38.54 (d, ^2^*J*_C–P_ = 12.1 Hz; C_14_), 37.35 (s; C_17_), 36.24 (s; C_23_), 30.84 (s; C_11_), 30.00 and 29.88 (two s; C_16_ and C_22_), 26.87, 26.62 and 24.36 (br; C_12_ and C_13_). C_1_ and C_2_ signals were not detected despite employing a broad chemical shift window and an extended acquisition time.

**^31^P{^1^H} NMR (202 MHz, C_6_D_6_):** δ (ppm) 202.1 (d, ^2^*J*_P–P_ = 63 Hz), 51.6 (d, ^2^*J*_P–P_ = 63 Hz).

**^31^P NMR (202 MHz, C_6_D_6_):** δ (ppm) 202.1 (d, ^2^*J*_P–P_ = 63 Hz), 51.6 (d, ^2^*J*_P–P_ = 63 Hz).


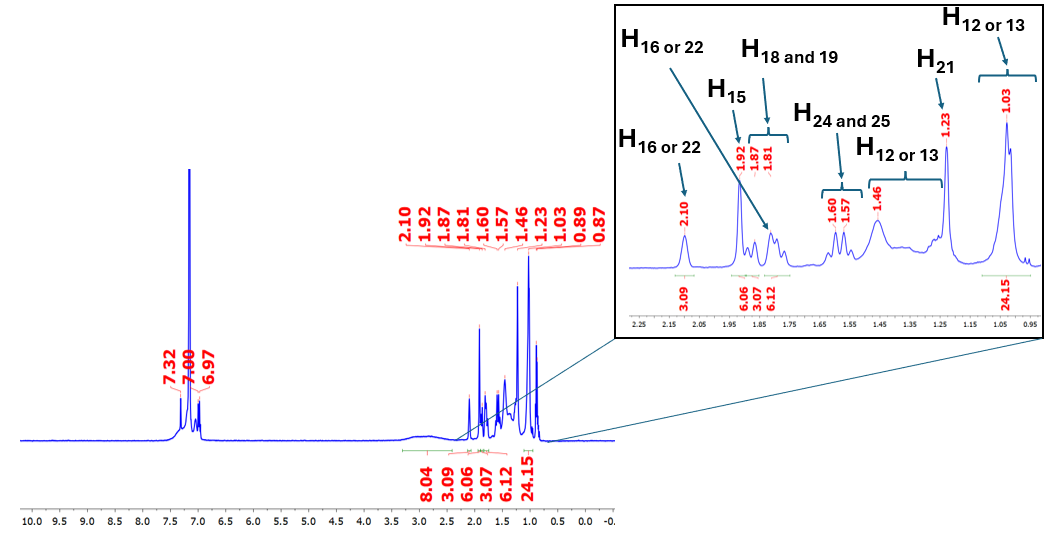


**Figure S8.** ^1^H NMR spectrum (298 K, C_6_D_6_, 500 MHz) of **2**.


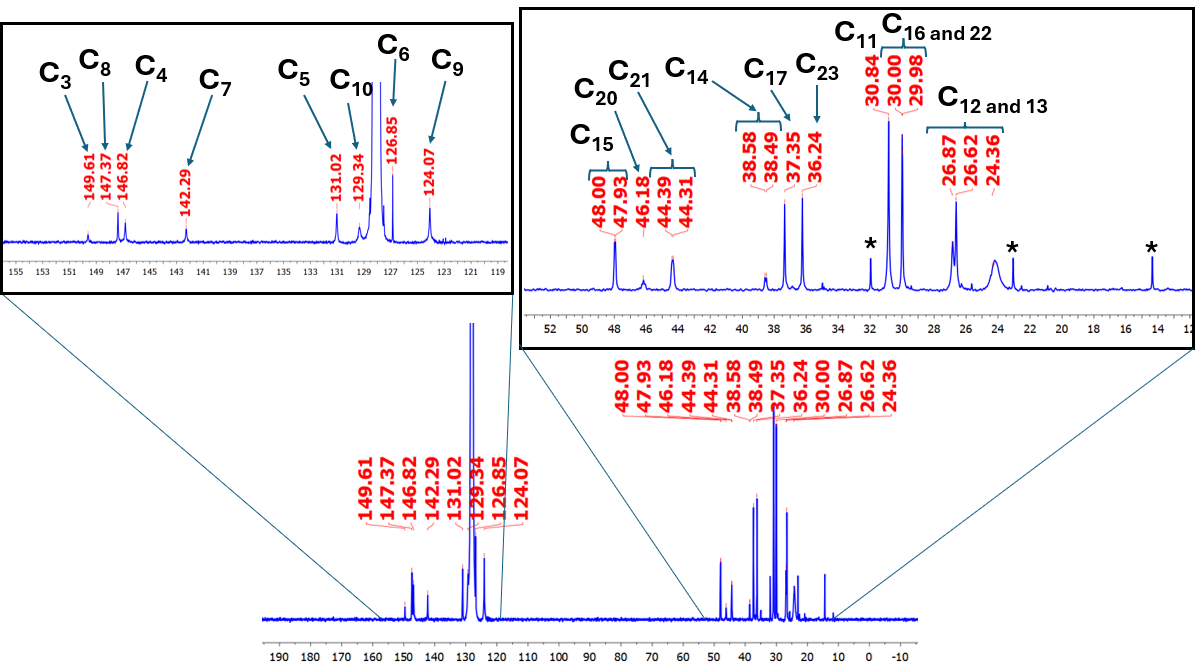


**Figure S9.** ^13^C{^1^H} NMR spectrum (298 K, C_6_D_6_, 500 MHz) of **2**. Note: *Residual hexane signals.


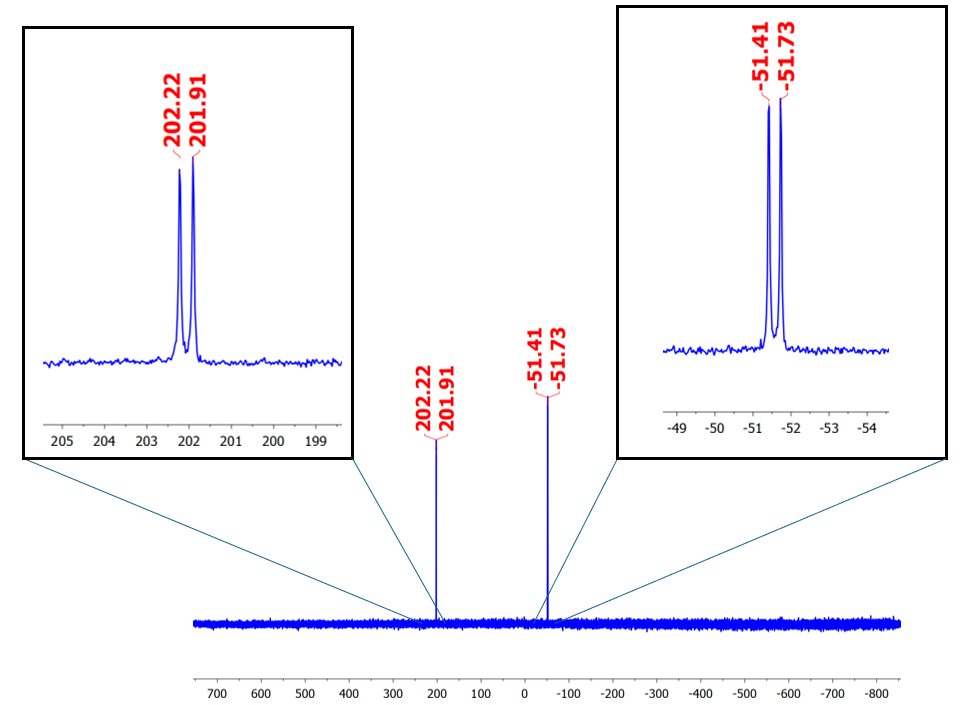


**Figure S10.** ^31^P{^1^H} NMR spectrum (298 K, C_6_D_6_, 202 MHz) of **2.**

## 2.3 NMR spectra of compound **3**

**Figure S11.** Compound **3**, In(Me)I[C(Ad)=PTer], with the atom labelling used for the assignment of NMR spectra.

**^1^H NMR (500 MHz, C_6_D_6_):** δ (ppm) 7.21 (t, ^3^*J*_H–H_ = 7.8 Hz, 2H; H_8_), 7.12–7.02 (br, 4H; H_7_, overlapped with H_3_), 7.06 (d, 2H, ^3^*J*_H–H_ = 7.6 Hz; H_3_ overlapped with H_7_), 6.69 (d, ^3^*J*_H–H_ = 7.6 Hz, 1H; H_4_), 3.15–2.73 (br, 4H; H_9_), 1.96 (br, 6H; H_14_), 1.84 (br, 3H; H_15_), 1.48 (d, ^2^*J*_H–H_ = 12.10 Hz, 3H; H_17_), 1.43–1.31 (m, 15H; H_10_ or H_11_ + H_18_), 1.14–0.86 (br, 12H; H_10_ or H_11_), 0.41 (s, 3H; H_19_).

**^13^C{^1^H} NMR (125 MHz, C_6_D_6_):** δ (ppm) 222.75 (d, ^1^*J*_C–P_ = 70.5; C_12_), 146.72 (br; C_6_), 145.46 (d, ^2^*J*_C–P_ = 5.3 Hz; C_2_), 144.38 (d, ^2^*J*_C–P_ = 45.5 Hz; C_1_), 138.06 (s; C_5_), 130.77 (s; C_3_), 129.61 (s; C_8_), 129.26 (s; C_4_), 123.54 (br; C_7_), 47.10 (d, ^2^*J*_C–P_ = 13.7 Hz; C_13_), 44.93 (d, ^3^*J*_C–P_ = 20.3 Hz; C_14_), 36.02 (s; C_16_), 31.72 (s; C_9_), 29.36 (s; C_15_), 25.67 and 23.35 (s; C_10_ and C_11_), 15.25 (s; C_19_).

**^31^P{^1^H} NMR (202 MHz, C_6_D_6_):** δ (ppm) 328.8 (s).

**^31^P NMR (202 MHz, C_6_D_6_):** δ (ppm) 328.8 (s).


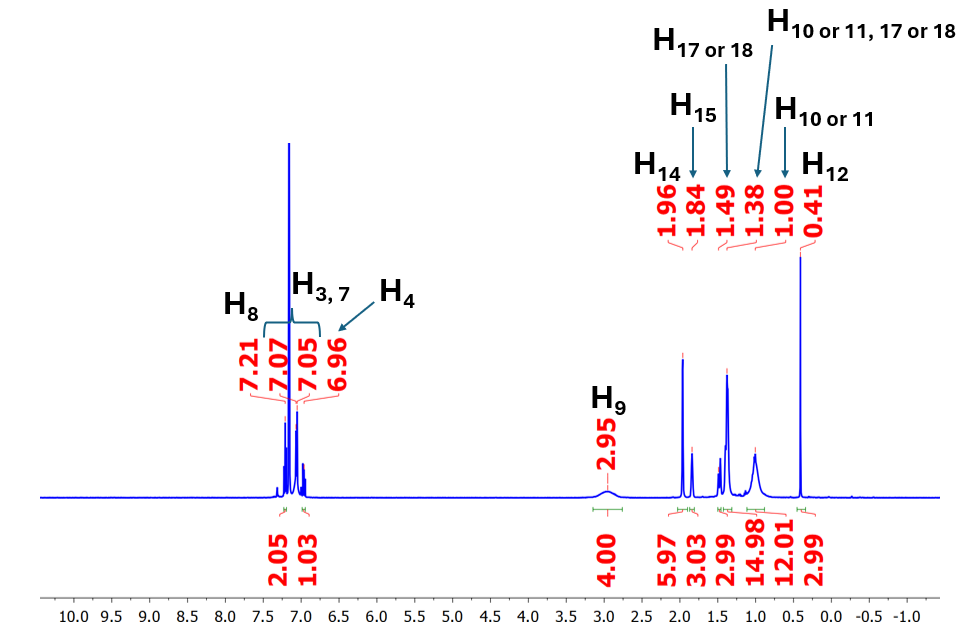


**Figure S12.** ^1^H NMR spectrum (298 K, C_6_D_6_, 500 MHz) of **3**.


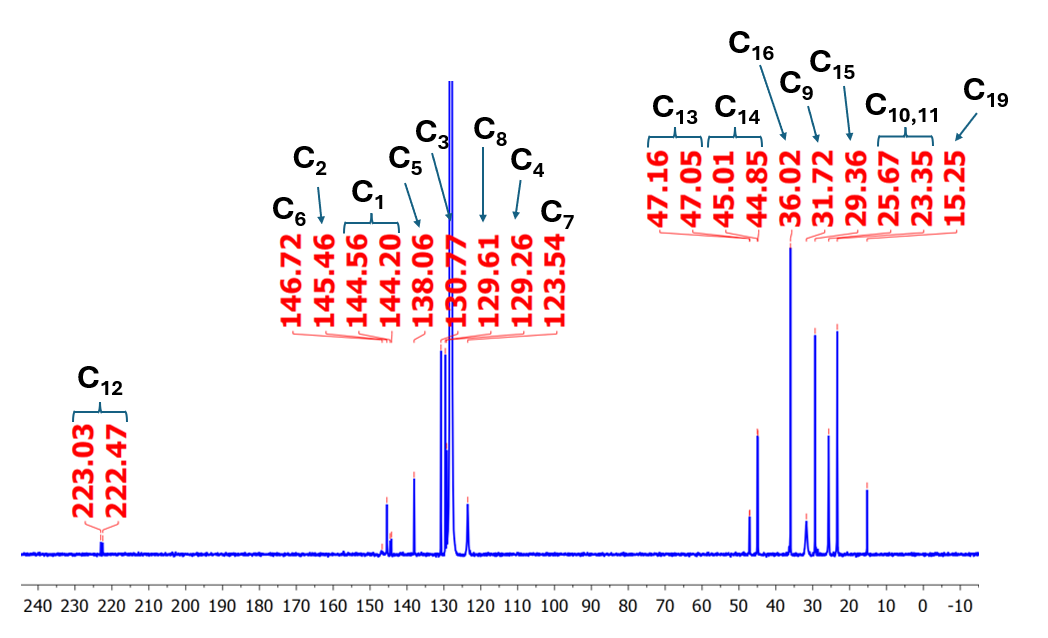


**Figure S13.** ^13^C{^1^H} NMR spectrum (298 K, C_6_D_6_, 500 MHz) of **3**.


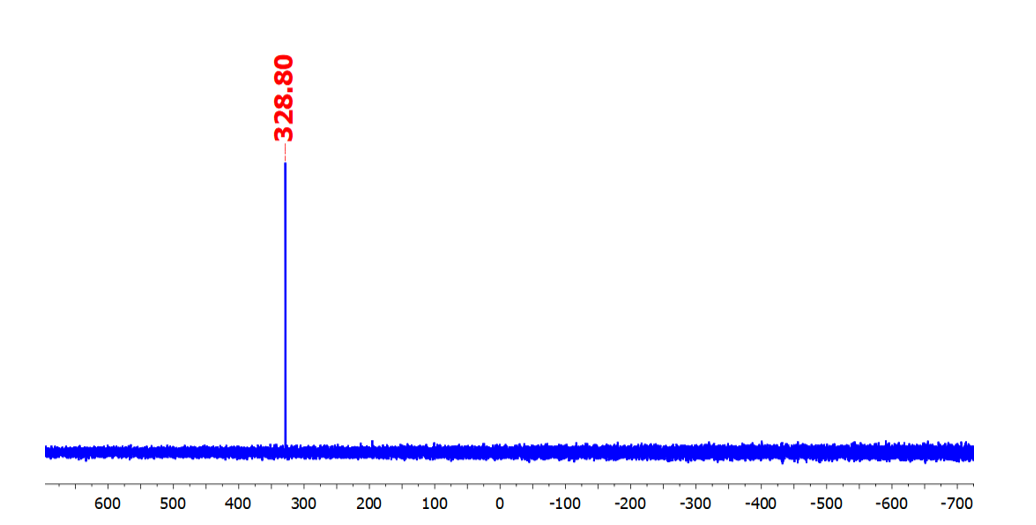


**Figure S14.** ^31^P{^1^H} NMR spectrum (298 K, C_6_D_6_, 202 MHz) of **3**.

## 2.4 NMR spectra of compound **4**

**Figure S15.** Compound **4**, {TerInMe(μ-I)}_2_, with the atom labelling used for the assignment of NMR spectra.

**^1^H NMR (500 MHz, C_6_D_6_):** δ (ppm) 7.30–7.19 (m; 10H, H_3_ + H_4_ + H_8_), 7.14 (d, ^3^*J*_H–H_ = 7.9 Hz, 8H; H_7_), 3.01 (sept, ^3^*J*_H–H_ = 6.9 Hz, 8H; H_9_), 1.30 (d, ^3^*J*_H–H_ = 6.9 Hz, 24H; H_10_ or H_11_), 1.02 (d, ^3^*J*_H–H_ = 6.9 Hz, 24H; H_10_ or H_11_), 0.11 (s, 6H; H_12_).

**^13^C{^1^H} NMR (125 MHz, C_6_D_6_):** δ (ppm) 159.83 (s; C_1_), 146.98 (s; C_6_), 146.69 (s; C_2_) 141.44 (s; C_5_), 129.60 (s; C_8_), 128.54 (s; C_3_), 124.14 (s; C_7_), 31.71 (s; C_9_), 26.15 and 23.20 (s; C_10_ and C_11_), 8.68 (s; C_12_).


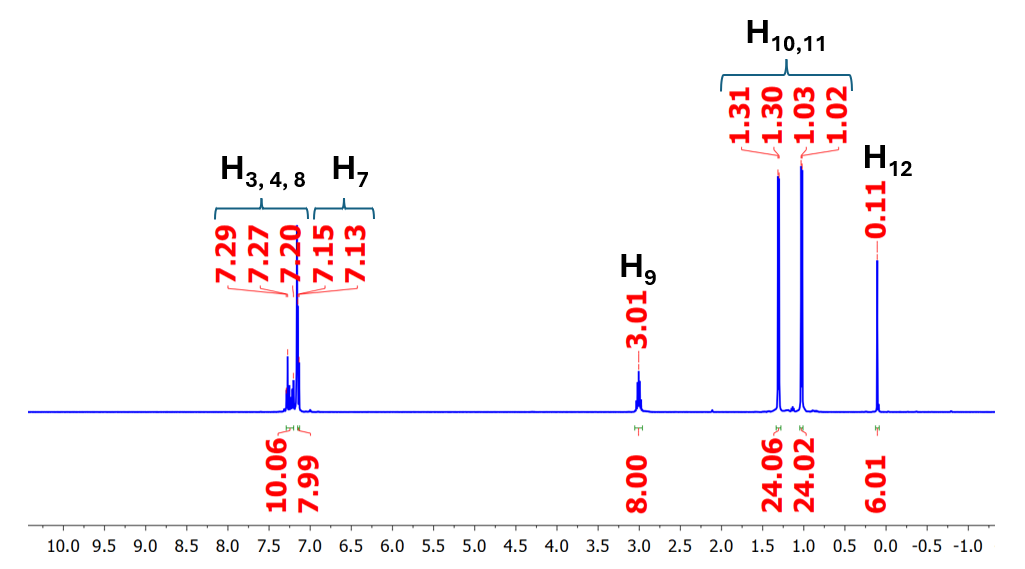


**Figure S16.** ^1^H NMR spectrum (298 K, C_6_D_6_, 500 MHz) of **4**.


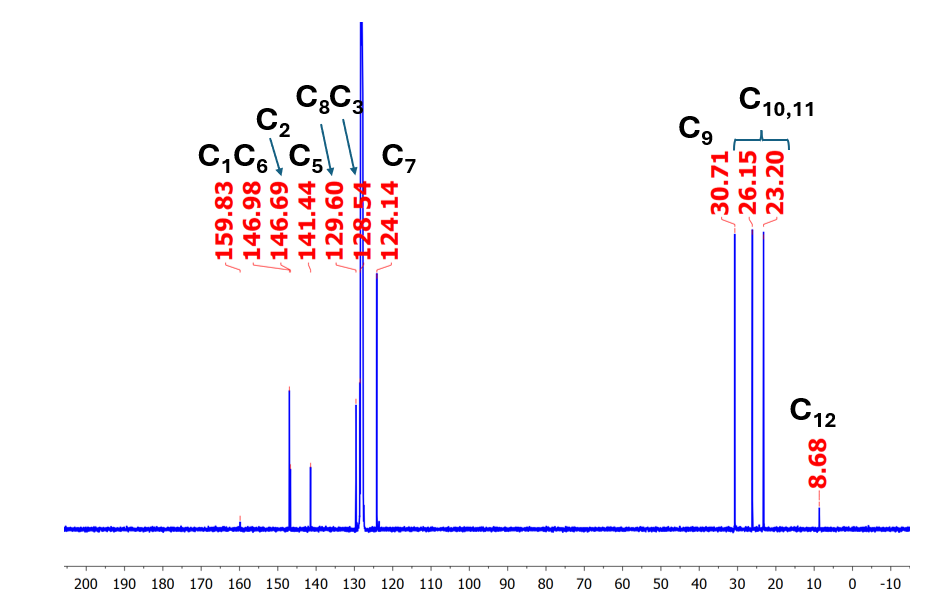


**Figure S17.** ^13^C{^1^H} NMR spectrum (298 K, C_6_D_6_, 500 MHz) of **4**.

# VT-NMR experiments


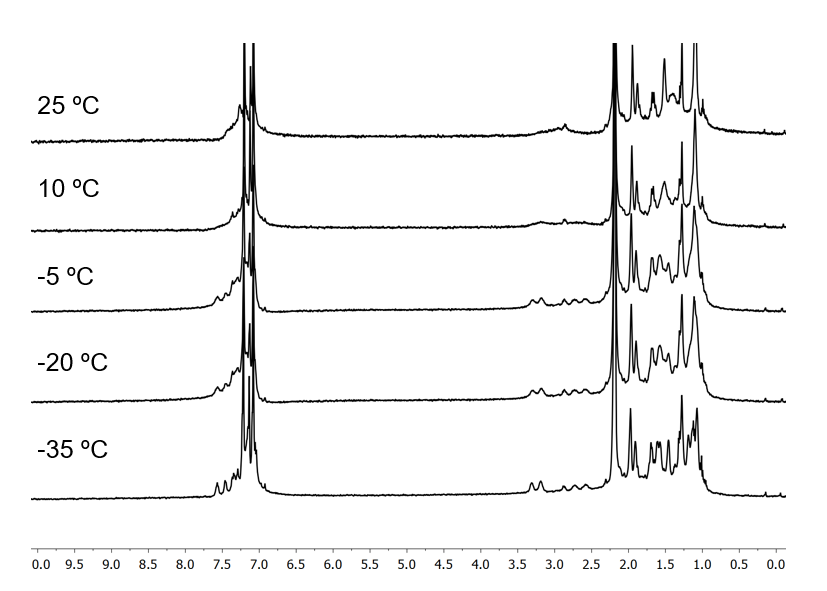


**Figure S18**. Variable-temperature ^1^H NMR spectra of a sample of (GaTer)_2_(AdCP)_2_ (**2**) in d^8^-toluene solution. The room temperature spectrum shows a significant broadening of the Ter groups resonances. Reducing the temperature below –5 ºC results in the splitting of the signals, indicating a rigid conformation where the rotation of the Ter groups is restricted.


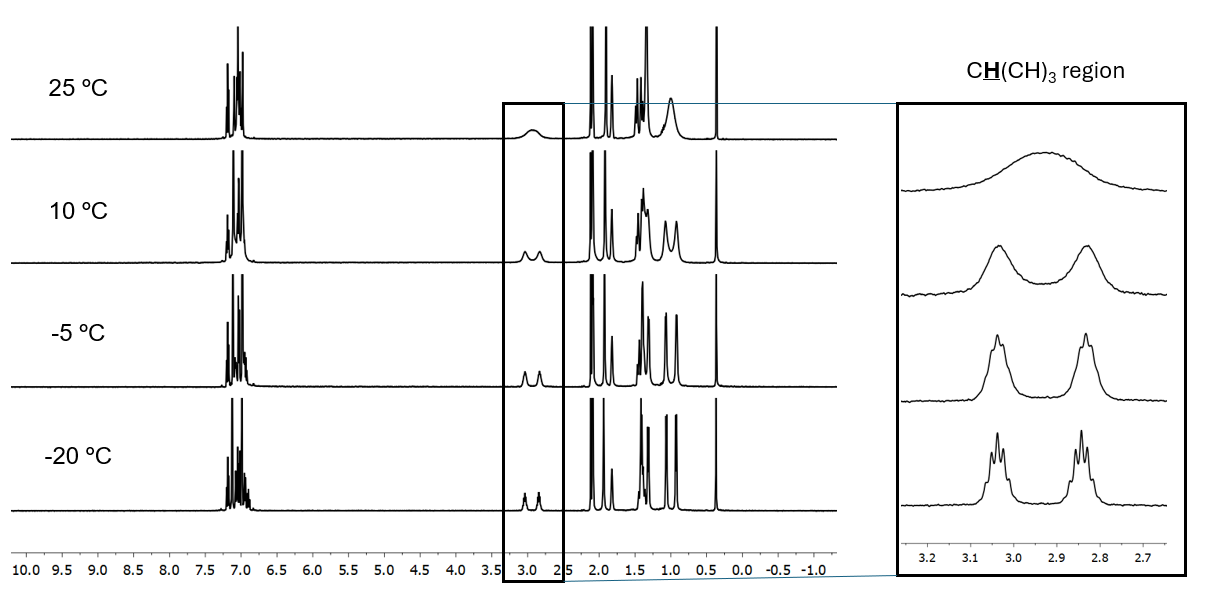


**Figure S19.** Variable-temperature ^1^H NMR spectra of a sample of In(Me)I[C(Ad)=PTer] (**3**) in d^8^-toluene solution. The room temperature spectrum shows a significant broadening of the CH(CH_3_)_2_ resonances. Reducing the temperature below 10 ºC results in the splitting in two inequivalent CH(CH_3_)_2_ signals, indicating a rigid conformation where the rotation of the Ter groups is restricted.


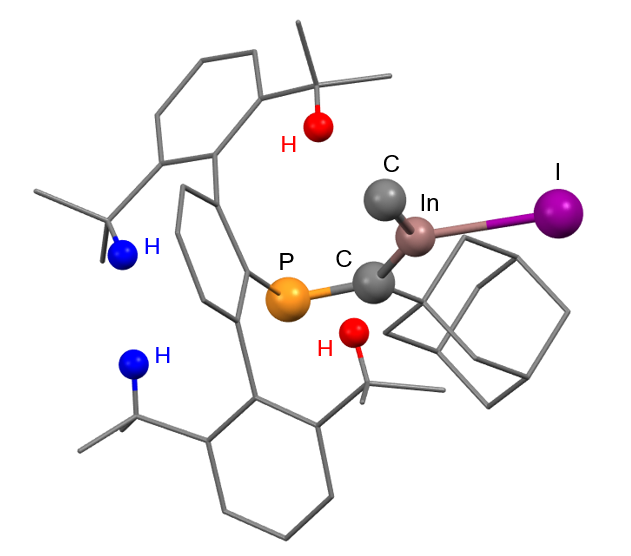


**Figure S20.** Proposed two pairs of inequivalent CH(CH_3_)_2_ protons (highlighted in red and blue) observed in the VT ^1^H-NMR experiments of In(Me)I[C(Ad)=PTer] (**3**) due to the restricted rotation of the Ter groups, based on the X-ray structure.

# *In situ* NMR experiments


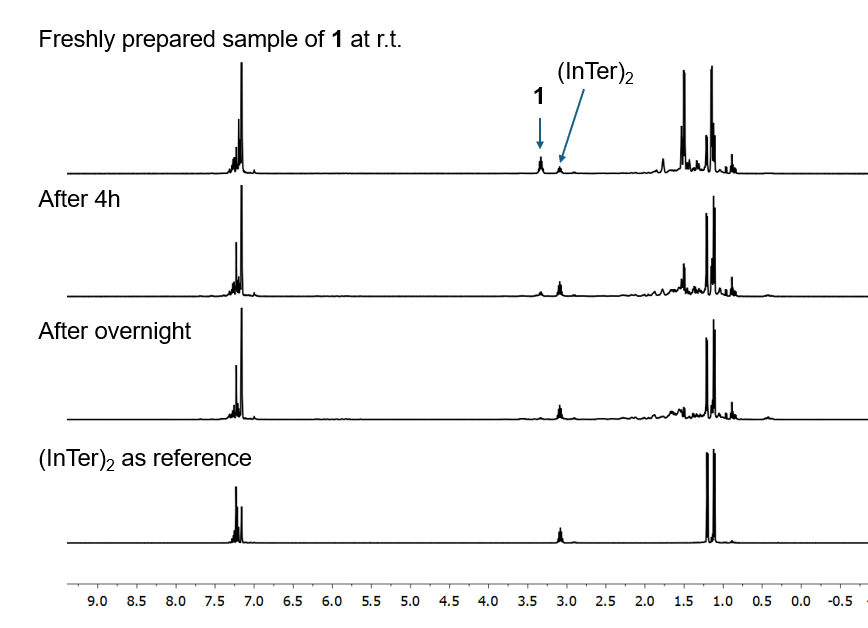


**Figure S21**. ^1^H NMR stacked spectra of the evolution over time of a sample of **1** in C_6_D_6_ solution at room temperature showing the gradually formation of (InTer)_2_ and other unknown decomposition products.


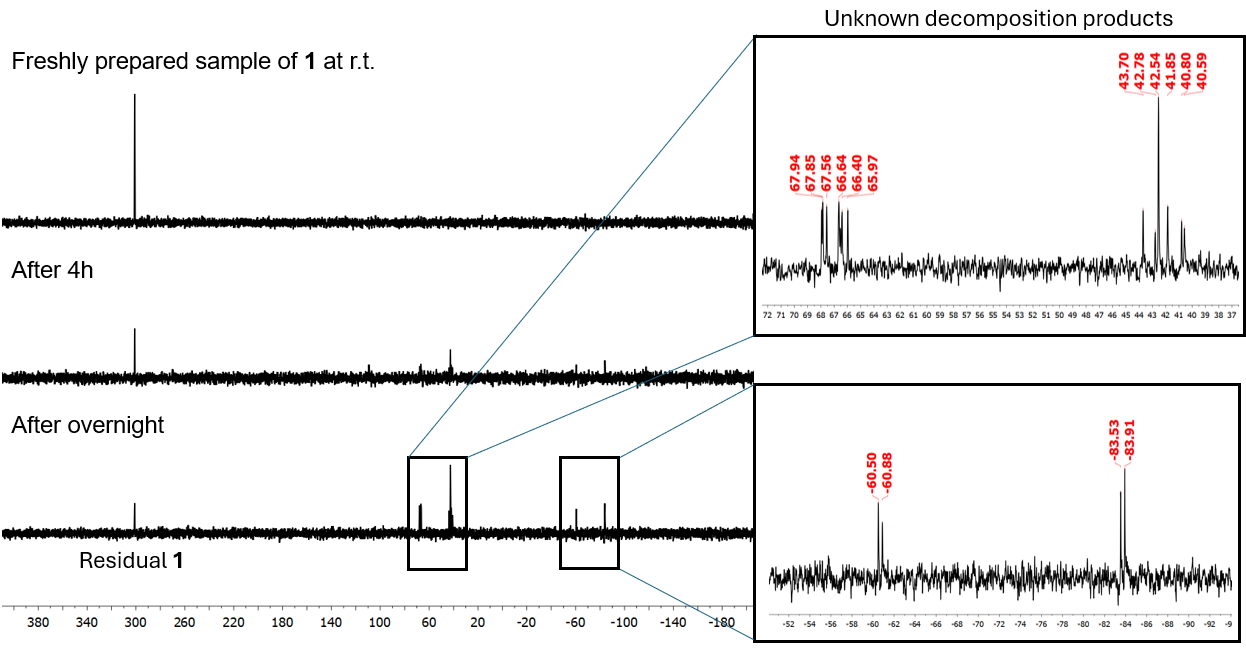


**Figure S22**. ^31^P{^1^H} NMR stacked spectra of the evolution over time of a freshly prepared sample of **1** in C_6_D_6_ solution at room temperature showing the formation of phosphorous-containing decomposition products.

*In situ* NMR scale reaction of **1** with B(C_6_F_5_)_3_ at room temperature:

Compound **1** was synthetized on an NMR scale by the addition of a solution of AdCP (0.0058 mmol, 1 mg) in C_6_D_6_ (≈ 0.3 ml) to a solution of (InTer)_2_ (0.0029 mmol, 3 mg) in C_6_D_6_ (≈ 0.3 ml). The ^1^H and ^31^P{^1^H} were immediately recorded at room temperature. After confirming the formation of **1** by NMR spectroscopy, in the same J. Young NMR tube, a solution of B(C_6_F_5_)_3_ (0.0058 mmol, 3 mg) in C_6_D_6_ (≈ 0.3 ml) were added, resulting in immediately colour change, from bright red to pale yellow. The ^1^H and ^31^P NMR spectra show the quantitative conversion of **1** to TerInB(C_6_F_5_)_3_ and AdCP after the B(C_6_F_5_)_3_ addition.


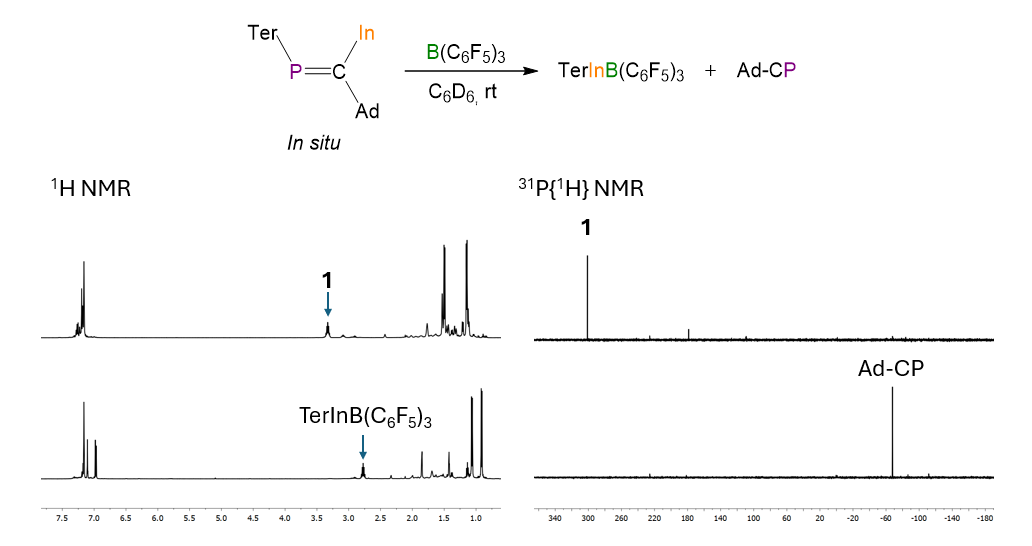


**Figure S23.** ^1^H and ^31^P{^1^H} NMR of the in situ reaction of **1** with ≈1 equivalent of B(C_6_F_5_)_3_ in C_6_D_6_ solution at room temperature showing the formation of [TerInB(C_5_F_6_)_3_] and Ad-CP as major products.

*In situ* reaction of **1** with B(C_6_F_5_)_3_ at –78 °C:

To a solution of (InTer)_2_ (50 mg, 0.049 mmol) in toluene (3 ml) at –78 °C, a solution of AdCP (17.4 mg, 0.098 mmol, 2 eq) in toluene (3 ml) was added dropwise to yield a bright red solution. After 10 mins, a solution of B(C_6_F_5_)_3_ (49.9 mg, 0.098 mmol) in toluene (3 ml) was added dropwise. The solution immediately lightened in color, from bright red to a pale-yellow solution. After 10 mins, the resulting solution was allowed to reach room temperature. All the volatiles were removed under vacuum. The resulting residue was extracted with 5 ml of hexane, concentrated to an approx. volume of 3 ml, and storage at –35 °C to yield [TerInB(C_6_F_5_)_3_] as colorless crystals suitable for X-ray diffraction. Yield: 57.3 mg (0.056 mmol, 57 %). All analytical data match literature reported values.

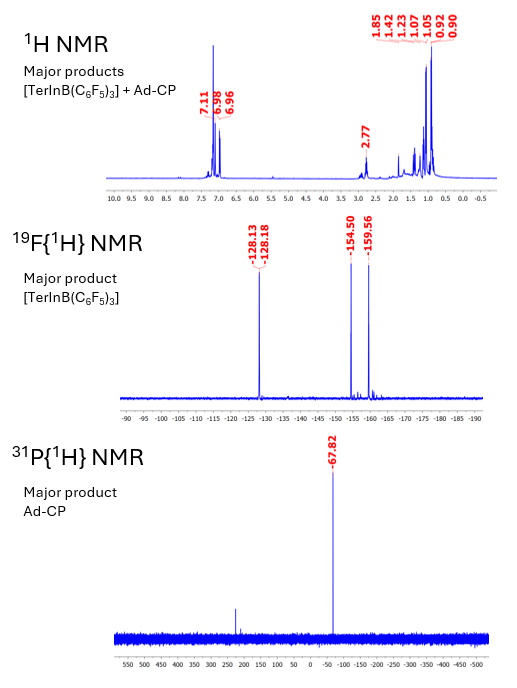


**Figure S24.** ^1^H, ^19^F[{^1^H} and ^31^P{^1^H} NMR spectra of the reaction crude of **1** with 1 equivalent of B(C_6_F_5_)_3_ (see experimental part for reaction conditions) in C_6_D_6_ solution showing the formation of [TerInB(C_5_F_6_)_3_] and Ad-CP as major products. The formation of minor impurities is observed in the ^1^H, ^19^F{^1^H} and ^31^P{^1^H} NMR spectra.


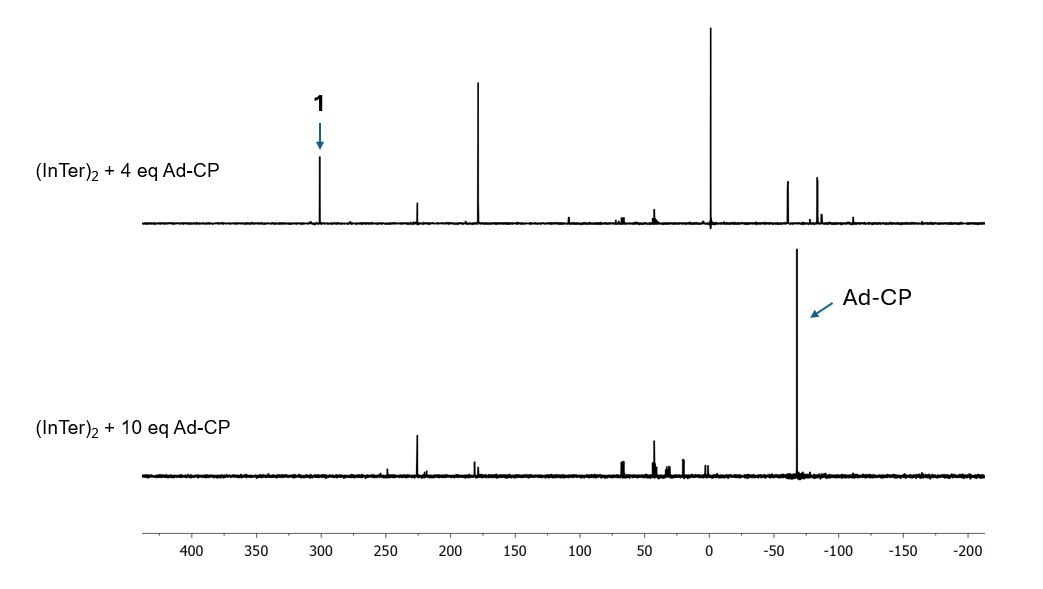


**Figure S25.** ^31^P{^1^H} NMR spectra of the reaction of (InTer)_2_ with 4 and 10 equivalents of AdCP (top and bottom, respectively) in C_6_D_6_ solution at room temperature.

# High resolution mass data


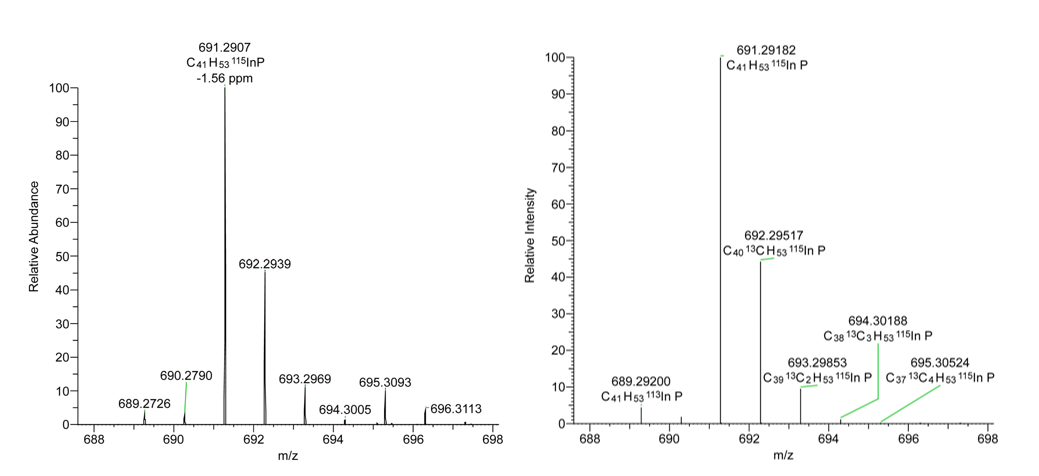


**Figure S26.** HR-MS (APCI-Orbitrap) (positive mode) of In[(Ad)C=PTer] (**1**) showing the [M+H]^+^ peak a m/z 691.2907 (calcd 691.2918; –1.56 ppm error) (left) and simulation (right).


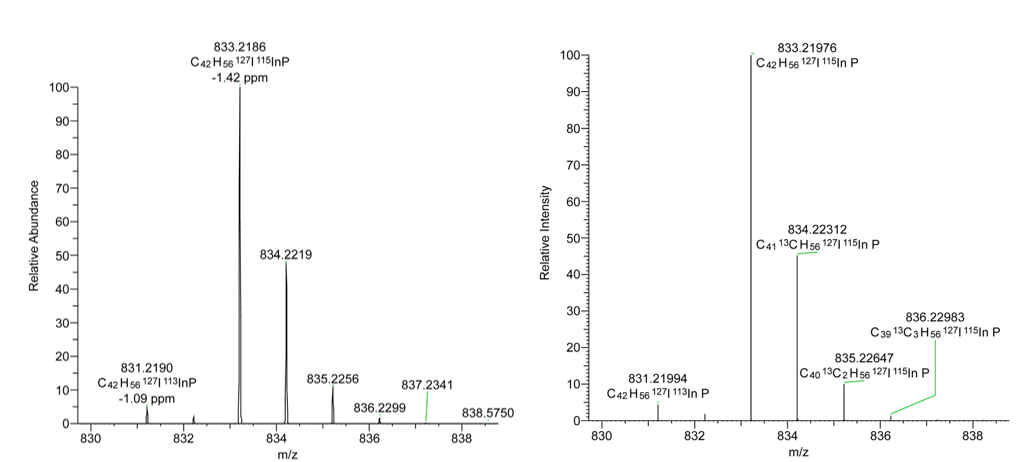


**Figure S27.** HR-MS (APCI) (positive mode) of In(Me)I[C(Ad)=PTer] (**3**) showing the [M+H]^+^ peak a m/z 833.2186 (calcd. 833.2197; –1.42 ppm error) (left) and simulation (right).


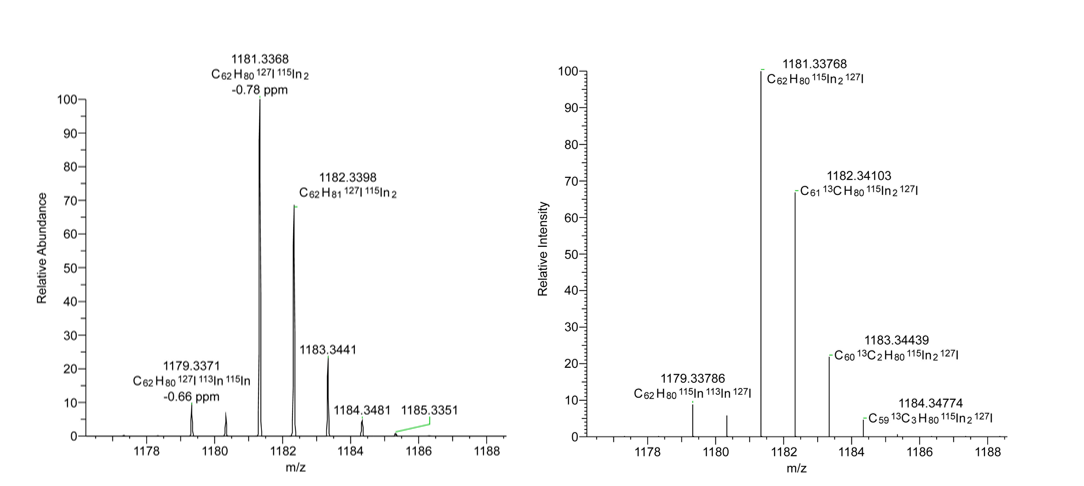


**Figure S28.** HR-MS (APCI) (positive mode) of {TerInMe(μ-I)}_2_ (**4**) showing the [M-I]^+^ peak a m/z 1181.3368 (calcd. 1181.3376; –0.78 ppm error) (left) and simulation (right).

# In situ UV-Vis experiments

UV-Vis spectra were recorded in toluene in a UV-Vis cuvette with equipped with a septum, filled in the glovebox and sealed with electrical tape. The spectrum of (InTer)_2_ was recorded in toluene at *–*78 ºC. The *in situ* addition of 2 equivalents of AdCP at *–*78 ºC resulted in the complete consumption of (InTer)_2_ and the formation of **1**, as evidenced by UV-Vis. Finally, the addition of 2 equivalents of B(C_6_F_5_)_3_ resulted in the quantitative consumption of **1**.


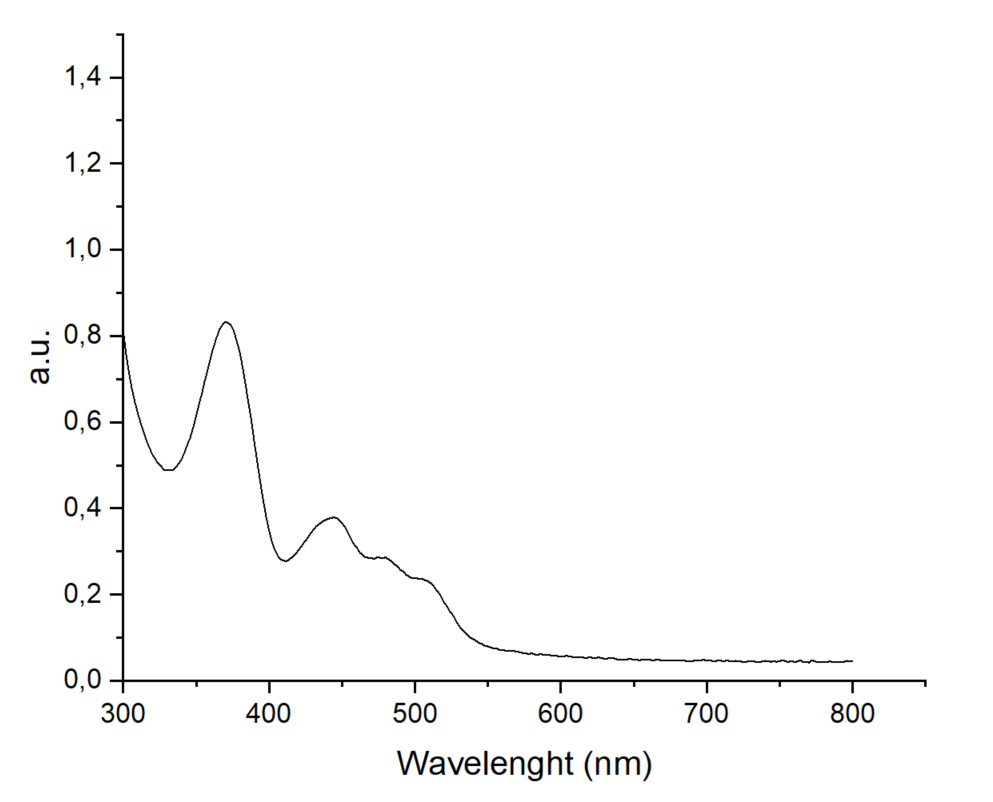


**Figure S29.** UV-Vis spectrum of (InTer)_2_ in toluene at –78 ºC.


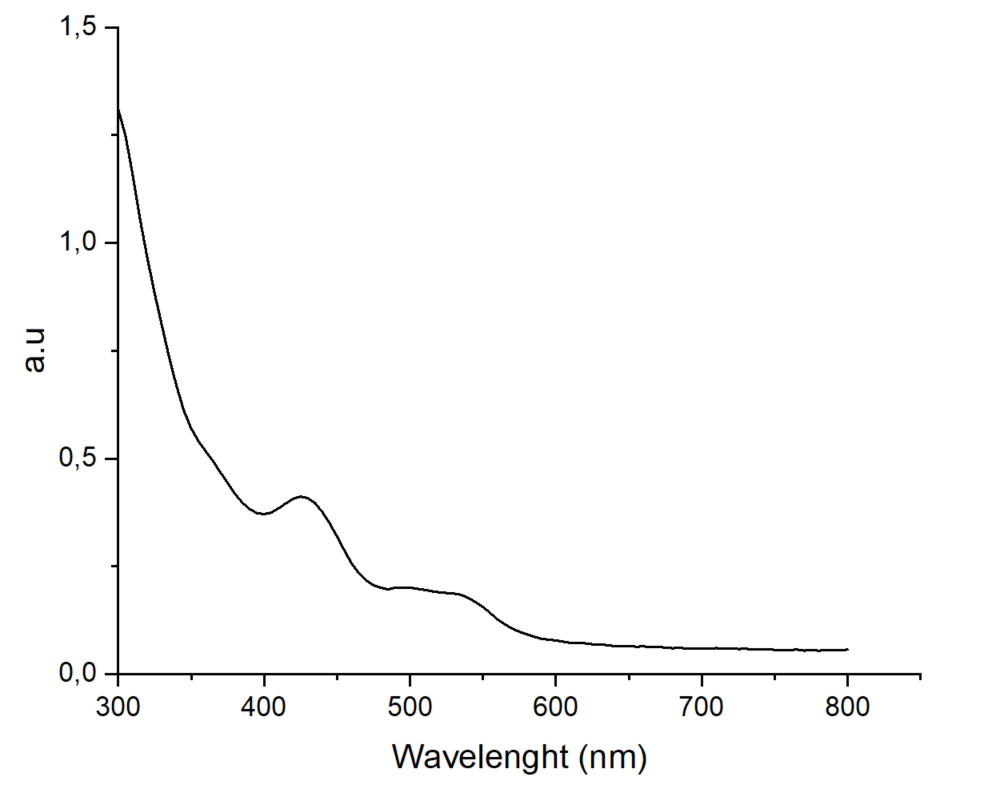


**Figure S30.** UV-Vis spectrum of the in situ reaction of (InTer)_2_ with 2 equivalents of Ad-CP in toluene at –78 ºC to yield **1**.


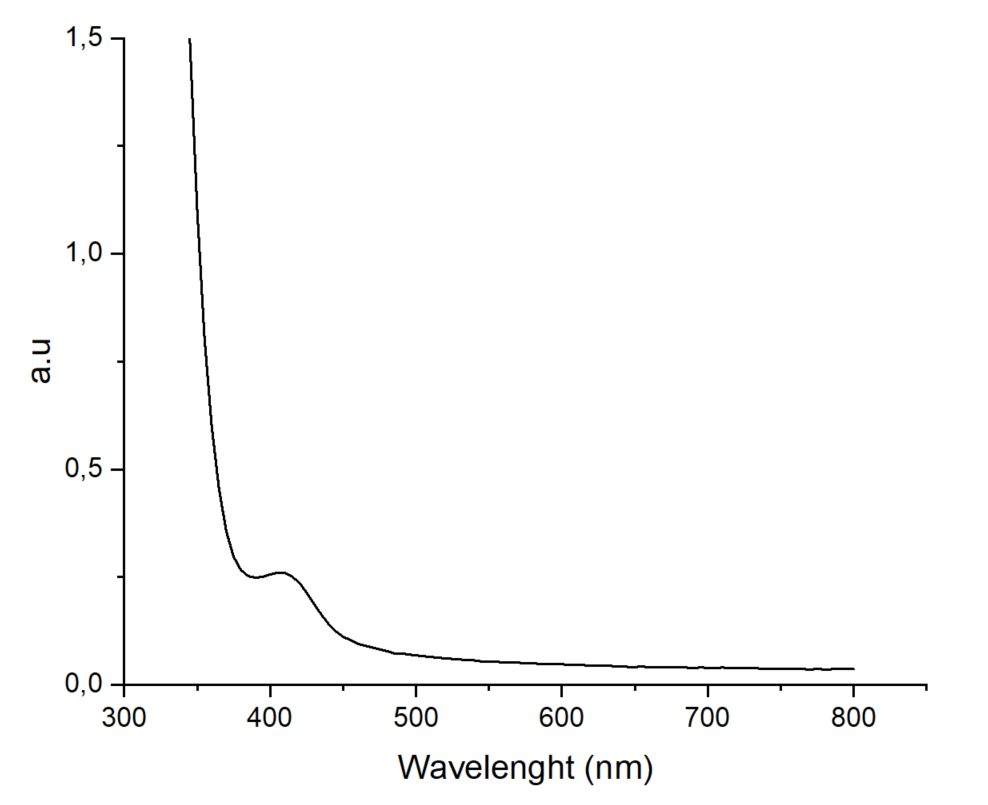


**Figure S31.** UV-Vis spectrum of the in situ reaction of **1** with 1 equivalent of B(C_6_F_5_)_3_ in toluene at –78 ºC.


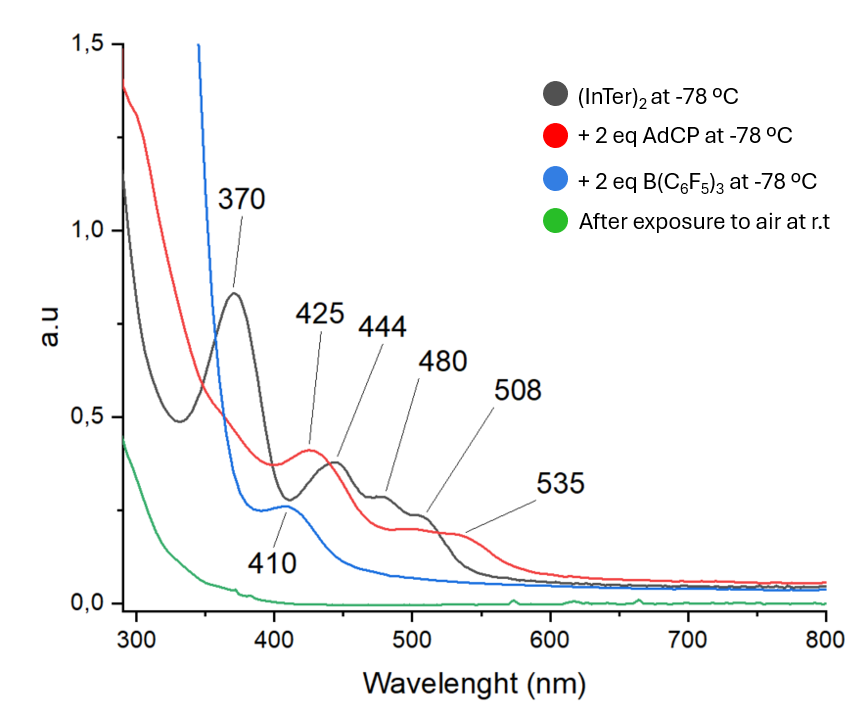


**Figure S32.** Stacked UV-Vis spectra of (InTer)_2_ in toluene at –78 ºC (gray); after the addition of 2 equivalents of AdCP at –78 ºC (red); after the addition of 2 equivalents B(C_6_F_5_)_3_ at –78 ºC (blue) and after exposing the mixture to air at room temperature (green).

# X-ray crystallographic studies

**Table S1.** Crystal and structure refinement data for **1** (CCDC 2496753)

Empirical formula C_41_H_52_InP

Formula weight 690.61

Crystal color, shape, size bright red block, 0.127 × 0.262 × 0.303 mm3

Temperature 173(2) K

Wavelength 0.71073 (Mo K_α_)

Crystal system, space group Monoclinic, *P*2_1_*/m*

Unit cell dimensions *a* = 10.0889(4) Å α = 90°

*b* = 17.1428(10) Å β = 100.178(2)°

*c* = 10.3784(6) Å γ = 90°

Volume 1766.72(16) Å3

*Z* 2

Density (calculated) 1.298 mg/m3

Absorption coefficient 0.741 mm–1

*F*(000) 724

***Data collection***

Diffractometer Venture D8, Bruker

Source Iμ3.0, Incoatec

Detector Photon III

Theta range for data collection 2.00 to 27.51°

Index ranges –13 ≤ h ≤ 12, –22 ≤ k ≤ 22, –12 ≤ l ≤ 13

Reflections collected 91884

Independent reflections 4190 [*R*int = 0.0869; *R*_sigma_ = 0.0395]

Observed Reflections 3998

Completeness to theta = 25.242° 100%

***Solution and Refinement***

Absorption correction Multi-scan

Max. and min. transmission 0.912 and 0.807

Solution Intrinsic methods

Refinement method Full-matrix least-squares on *F*2

Weighting scheme w = [σ2Fo2 + AP2 + BP]–1, with

P = (F_o_2 + 2 F_c_2)/3, A = 0.0276, B = 1.8051

Data / restraints / parameters 4190 / 298 / 234

Goodness-of-fit on *F*2 1.116

Final *R* indices [*I*>2σ(I)] *R*_1_ = 0.0358, *wR*_2_ = 0.0877

*R* indices (all data) *R*_1_ = 0.0388, *wR*_2_ = 0.0897

Largest diff. peak and hole 0.42 and –0.76 e·Å–3

Goodness-of-fit = [Σ[*w*(F_o_^2^ − F_c_^2^)^2^]/N_observns_ − N_params_)]^1/2^, all data. *R*_1_ = Σ(|F_o_| − |F_c_|) / Σ |F_o_|. *wR*_2_ = [Σ[*w*(F_o_^2^ − F_c_^2^)^2^] / Σ [*w*(F_o_^2^)^2^]]^1/2^.

**Crystal structure data for 1**

A bright red, block-shaped specimen of **1** (C_41_H_52_InP; IUMSC 24349), approximate dimensions 0.127 × 0.262 × 0.303 mm^3^, was placed on a Kapton mount with inert oil for crystal structure determination. The X-ray intensity data were measured with a microfocus sealed tube (λ = 0.71073 Å) and a multilayer mirror monochromator.

**Data collection:** The data collection was performed using 1° ω and φ scans, frame times of 1 and 20 s, and a detector distance of 40.00 mm. Overall, 1350 frames were collected with a total exposure time of 3.94 hours. The frames were integrated with the SAINT V8.41 package using a narrow-frame algorithm.⁠^[56]^ The integration of the data using a monoclinic unit cell yielded 91884 reflections to a maximum θ angle of 27.51° (0.77 Å resolution), of which 4190 were independent (average redundancy 25.00, completeness = 100.0%, *R*_int_ = 8.69%, *R*_sig_ = 3.95%) and 3998 (95.4%) were greater than 2σ(*F*^2^). The final cell constants of a = 10.0889(4) Å, b = 17.1428(10) Å, c = 10.3784(6) Å, α = 90°, β = 100.178(2)°, γ = 90°, volume = 1766.72(16) Å^3^, are based upon the refinement of the XYZ-centroids of 9831 reflections above 20 σ(*I*) with 2.32° < 2θ < 27.48°. Data were corrected for absorption effects using the Multi-Scan method in TWINABS Bruker.^[57]^ The calculated minimum and maximum transmission coefficients (based on crystal size) are 0.807 and 0.912.⁠ Additional crystal and refinement information can be found in the tables.

**Structure solution and refinement:** The space group *P*2_1_/*m* (11) was determined based on intensity statistics and systematic absences. The structure was solved by SHELXT 2018/2 and refined with full-matrix least squares / difference Fourier cycles using SHELXL-2019/2; *Z* = 2 for the formula unit C_41_H_52_InP.⁠^[58,59]^ Non-hydrogen atoms were refined with anisotropic displacement parameters. The hydrogen atoms were placed in ideal positions and refined as riding atoms with relative isotropic displacement parameters. The final anisotropic full-matrix least-squares refinement on *F*^2^ with 234 variables against 4190 data points and 298 restraints converged at *R*_1_ = 3.58%, for the observed data and w*R*_2_ = 8.97% for all data. The goodness-of-fit on *F*^2^ was 1.12. The largest peak in the final difference electron density synthesis was 0.42 e^−^/Å^3^ and the deepest hole was −0.76 e^−^/Å^3^ with an RMS deviation of 0.081 e^−^/Å^3^. On the basis of the final model, the calculated density was 1.30 g/cm^3^ and *F*(000), 724 e^−^. Refined as a 2-component twin; twin law 1, 0, 0; 0, –1, 0; –0.361, 0, –1; 180° rotation about 1.000 0.000 0.000 (direct space); Domain ratio 52:49 (BASF 0.49).

**Table S2.** Crystal and structure refinement data for **2**·4tol (CCDC 2496754)

Empirical formula C_110_H_136_Ga_2_P_2_

Formula weight 1659.56

Crystal color, shape, size yellow needle, 0.060 × 0.060 × 0.080 mm3

Temperature 153(2) K

Wavelength 1.54178 (Cu K_α_)

Crystal system, space group Orthorhombic, *Pbca*

Unit cell dimensions *a* = 28.3654(11) Å α = 90°

*b* = 20.7939(8) Å β = 90°

*c* = 29.8370(14) Å γ = 90°

Volume 17598.7(13) Å3

*Z* 8

Density (calculated) 0.975 mg/m3

Absorption coefficient 1.345 mm–1

*F*(000) 7104

***Data collection***

Diffractometer Venture D8, Bruker

Source Iμ3.0, Incoatec

Detector Photon III

Theta range for data collection 2.96 to 68.76°

Index ranges –34 ≤ h ≤ 31, –25 ≤ k ≤ 25, –36 ≤ l ≤ 36

Reflections collected 331855

Independent reflections 16219 [*R*int = 0.1167; *R*_sigma_ = 0.0656]

Observed Reflections 12833

Completeness to theta = 67.679° 100%

***Solution and Refinement***

Absorption correction Multi-scan

Max. and min. transmission 0.753 and 0.535

Solution Intrinsic methods

Refinement method Full-matrix least-squares on *F*2

Weighting scheme w = [σ2Fo2 + AP2 + BP]–1, with

P = (F_o_2 + 2 F_c_2)/3, A = 0.1086, B = 24.2151

Data / restraints / parameters 16219 / 0 / 775

Goodness-of-fit on *F*2 1.087

Final *R* indices [*I*>2σ(I)] *R*_1_ = 0.0847, *wR*_2_ = 0.2447

*R* indices (all data) *R*_1_ = 0.0962, *wR*_2_ = 0.2546

Largest diff. peak and hole 1.27 and –0.70 e·Å–3

Goodness-of-fit = [Σ[*w*(F_o_^2^ − F_c_^2^)^2^]/N_observns_ − N_params_)]^1/2^, all data. *R*_1_ = Σ(|F_o_| − |F_c_|) / Σ |F_o_|. *wR*_2_ = [Σ[*w*(F_o_^2^ − F_c_^2^)^2^] / Σ [*w*(F_o_^2^)^2^]]^1/2^.

**Crystal structure data for 2**·4tol

AA yellow, needle-shaped specimen of **2**·4tol (C_82_H_104_Ga_2_P_2_, IUMSC 25236_sq), approximate dimensions 0.06 × 0.06 × 0.08 mm^3^, was placed on a Kapton mount with inert oil for crystal structure determination. The X-ray intensity data were measured on a Bruker D8 Venture KAPPA diffractometer equipped with a microfocus sealed tube (λ = 1.54178 Å) and a multilayer mirror monochromator. The experiment was performed at 153(2) K using an Oxford Cryostream open-nitrogen-flow temperature control unit.

**Data collection:** The data collection was performed using 0.5 and 1° ω and φ scans, frame times of 1, 2, 28.70 and 60 s, and a detector distance of 40.00 mm. Overall, 5340 frames were collected with a total exposure time of 53.92 hours. The frames were integrated with the SAINT V8.41 package using a narrow-frame algorithm.⁠^[56]^ The integration of the data using an orthorhombic unit cell yielded 331855 reflections to a maximum θ angle of 68.76° (0.83 Å resolution), of which 16219 were independent (average redundancy 20.46, completeness = 100.0%, *R*_int_ = 11.67%, *R*_sig_ = 6.56%) and 12833 (79.1%) were greater than 2σ(*F*^2^). The final cell constants of a = 28.3654(11) Å, b = 20.7939(8) Å, c = 29.8370(14) Å, α = 90°, β = 90°, γ = 90°, volume = 17598.7(13) Å^3^, are based upon the refinement of the XYZ-centroids of 9525 reflections above 20 σ(*I*) with 3.02° < 2θ < 68.21°. Data were corrected for absorption effects using the Multi-Scan method in SADABS 2016/2.^[60]^ The calculated minimum and maximum transmission coefficients (based on crystal size) are 0.535 and 0.753.⁠ Additional crystal and refinement information can be found in the tables.

**Structure solution and refinement:** The space group *Pbca* (61) was determined based on intensity statistics and systematic absences. The structure was solved by SHELXT 2018/2 and refined with full-matrix least squares / difference Fourier cycles using SHELXL-2019/2; *Z* = 8 for the formula unit C_82_H_104_Ga_2_P_2_.⁠^[58,59]^ Non-hydrogen atoms were refined with anisotropic displacement parameters. The hydrogen atoms were placed in ideal positions and refined as riding atoms with relative isotropic displacement parameters. Remaining electron density indicated that additional partial solvent (toluene) was present in the structure. However, solvent models with strong sets of restraints and constraints did not converge to a chemically sensible structure. Therefore, the structure was investigated for solvent accessible areas.^[61]^ Two voids were found in the unit cell (~5280 Å^3^) to contain 1310 electrons. For comparison, hexane occupies ca. 148 Å^3^ with 50 electrons.^[62]^ Based on these values, we estimate that there are four molecules of hexane per formula unit. The contribution of the unidentified solvent to the structure factors was assessed by back-Fourier transformation^[61]^ and the data were corrected accordingly. The refinement using the modified dataset improved the overall structure and *R* values. The final anisotropic full-matrix least-squares refinement on *F*^2^ with 775 variables against 16219 data points and converged at *R*_1_ = 8.47%, for the observed data and w*R*_2_ = 25.46% for all data. The goodness-of-fit on *F*^2^ was 1.09. The largest peak in the final difference electron density synthesis was 1.27 e^−^/Å^3^ and the deepest hole was −0.70 e^−^/Å^3^ with an RMS deviation of 0.066 e^−^/Å^3^. On the basis of the final model, the calculated density was 0.97 g/cm^3^ and *F*(000), 7104 e^−^.

**Table S3.** Crystal and structure refinement data for **3**·C_6_H_12_ (CCDC 2496755)

Empirical formula C_48_ H_67_IInP

Formula weight 916.74

Crystal color, shape, size colourless block, 0.172 × 0.180 × 0.284 mm3

Temperature 173(2) K

Wavelength 0.71073 (Mo K_α_)

Crystal system, space group Triclinic, *P*–1

Unit cell dimensions *a* = 12.0934(3) Å α = 100.1850(10)°

*b* = 12.2873(4) Å β = 103.2800(10)°

*c* = 15.9004(5) Å γ = 95.3140(10)°

Volume 2241.31(12) Å3

*Z* 2

Density (calculated) 1.358 mg/m3

Absorption coefficient 1.282 mm–1

*F*(000) 944

***Data collection***

Diffractometer Venture D8, Bruker

Source Iμ3.0, Incoatec

Detector Photon III

Theta range for data collection 1.92 to 30.50°

Index ranges –17 ≤ h ≤ 16, –17 ≤ k ≤ 17, –22 ≤ l ≤ 22

Reflections collected 62545

Independent reflections 13738 [*R*int = 0.0448; *R*_sigma_ = 0.0337]

Observed Reflections 11731

Completeness to theta = 25.242° 99.8%

***Solution and Refinement***

Absorption correction Multi-scan

Max. and min. transmission 0.746 and 0.710

Solution Intrinsic methods

Refinement method Full-matrix least-squares on *F*2

Weighting scheme w = [σ2Fo2 + AP2 + BP]–1, with

P = (F_o_2 + 2 F_c_2)/3, A = 0.0328, B = 2.1714

Data / restraints / parameters 13738 / 0 / 460

Goodness-of-fit on *F*2 1.023

Final *R* indices [*I*>2σ(I)] *R*_1_ = 0.0341, *wR*_2_ = 0.0824

*R* indices (all data) *R*_1_ = 0.0424, *wR*_2_ = 0.0885

Largest diff. peak and hole 1.78 and –1.58 e·Å–3

Goodness-of-fit = [Σ[*w*(F_o_^2^ − F_c_^2^)^2^]/N_observns_ − N_params_)]^1/2^, all data. *R*_1_ = Σ(|F_o_| − |F_c_|) / Σ |F_o_|. *wR*_2_ = [Σ[*w*(F_o_^2^ − F_c_^2^)^2^] / Σ [*w*(F_o_^2^)^2^]]^1/2^.

**Crystal structure data for 3**·C_6_H_12_

A colourless, block-shaped specimen of **3**·C_6_H_12_ (C_48_H_67_IInP, IUMSC 25092), approximate dimensions 0.172 × 0.18 × 0.284 mm^3^, was placed on a Kapton mount with inert oil for crystal structure determination. The X-ray intensity data were measured on a Bruker D8 Venture KAPPA diffractometer equipped with a microfocus sealed tube (λ = 0.71073 Å) and a multilayer mirror monochromator.

**Data collection:** The data collection was performed using 0.5° ω and φ scans, frame times of 0.75 and 3 s, and a detector distance of 40.00 mm. Overall, 2706 frames were collected with a total exposure time of 1.41 hours. The frames were integrated with the SAINT V8.41 package using a narrow-frame algorithm.⁠^[56]^ The integration of the data using a triclinic unit cell yielded 62545 reflections to a maximum θ angle of 30.50° (0.70 Å resolution), of which 13738 were independent (average redundancy 4.55, completeness = 99.8%, *R*_int_ = 4.48%, *R*_sig_ = 3.37%) and 11731 (85.4%) were greater than 2σ(*F*^2^). The final cell constants of a = 12.0934(3) Å, b = 12.2873(4) Å, c = 15.9004(5) Å, α = 100.1850(10)°, β = 103.2800(10)°, γ = 95.3140(10)°, volume = 2241.31(12) Å^3^, are based upon the refinement of the XYZ-centroids of 9215 reflections above 20 σ(*I*) with 2.46° < 2θ < 30.36°. Data were corrected for absorption effects using the Multi-Scan method in SADABS 2016/2.^[60]^ The calculated minimum and maximum transmission coefficients (based on crystal size) are 0.710 and 0.746.⁠ Additional crystal and refinement information can be found in the tables.

**Structure solution and refinement:** The space group *P*–1 (2) was determined based on intensity statistics and systematic absences. The structure was solved by SHELXT 2018/2 and refined with full-matrix least squares / difference Fourier cycles using SHELXL-2019/2; *Z* = 2 for the formula unit C_48_H_67_IInP.⁠^[58,59]^ Non-hydrogen atoms were refined with anisotropic displacement parameters. The hydrogen atoms were placed in ideal positions and refined as riding atoms with relative isotropic displacement parameters. The final anisotropic full-matrix least-squares refinement on *F*^2^ with 460 variables against 13738 data points and converged at *R*_1_ = 3.41%, for the observed data and w*R*_2_ = 8.85% for all data. The goodness-of-fit on *F*^2^ was 1.02. The largest peak in the final difference electron density synthesis was 1.78 e^−^/Å^3^ and the deepest hole was −1.58 e^−^/Å^3^ with an RMS deviation of 0.078 e^−^/Å^3^. On the basis of the final model, the calculated density was 1.36 g/cm^3^ and *F*(000), 944 e^−^.

**Table S4.** Crystal and structure refinement data for **4** (CCDC 2496756)

Empirical formula C_62_H_80_I_2_In_2_

Formula weight 1308.70

Crystal color, shape, size colourless block, 0.079 × 0.259 × 0.260 mm3

Temperature 153(2) K

Wavelength 0.71073 (Mo K_α_)

Crystal system, space group Monoclinic, *P*2_1_/*n*

Unit cell dimensions *a* = 10.7389(4) Å α = 90°

*b* = 19.8380(9) Å β = 103.5720(10)°

*c* = 14.1538(7) Å γ = 90°

Volume 2931.1(2) Å3

*Z* 2

Density (calculated) 1.483 mg/m3

Absorption coefficient 1.875 mm–1

*F*(000) 1312

***Data collection***

Diffractometer Venture D8, Bruker

Source Iμ3.0, Incoatec

Detector Photon III

Theta range for data collection 1.80 to 28.30°

Index ranges –14 ≤ h ≤ 14, –26 ≤ k ≤ 26, –18 ≤ l ≤ 18

Reflections collected 119147

Independent reflections 7282 [*R*int = 0.0934; *R*_sigma_ = 0.0306]

Observed Reflections 6010

Completeness to theta = 25.242° 200%

***Solution and Refinement***

Absorption correction Multi-scan

Max. and min. transmission 0.746 and 0.679

Solution Intrinsic methods

Refinement method Full-matrix least-squares on *F*2

Weighting scheme w = [σ2Fo2 + AP2 + BP]–1, with

P = (F_o_2 + 2 F_c_2)/3, A = 0.0706, B = 4.0098

Data / restraints / parameters 7282 / 0 / 298

Goodness-of-fit on *F*2 1.246

Final *R* indices [*I*>2σ(I)] *R*_1_ = 0.0369, *wR*_2_ = 0.1105

*R* indices (all data) *R*_1_ = 0.0547, *wR*_2_ = 0.1420

Largest diff. peak and hole 1.08 and –2.22 e·Å–3

Goodness-of-fit = [Σ[*w*(F_o_^2^ − F_c_^2^)^2^]/N_observns_ − N_params_)]^1/2^, all data. *R*_1_ = Σ(|F_o_| − |F_c_|) / Σ |F_o_|. *wR*_2_ = [Σ[*w*(F_o_^2^ − F_c_^2^)^2^] / Σ [*w*(F_o_^2^)^2^]]^1/2^.

**Crystal structure data for 4**

A colourless, block-shaped specimen of **4** (C_62_H_80_I_2_In_2_, IUMSC 25190), approximate dimensions 0.079 × 0.259 × 0.26 mm^3^, was placed on a Kapton mount with inert oil for crystal structure determination. The X-ray intensity data were measured on a Bruker D8 Venture KAPPA diffractometer equipped with a microfocus sealed tube (λ = 0.71073 Å) and a multilayer mirror monochromator. The experiment was performed at 153(2) K using an Oxford Cryostream open-nitrogen-flow temperature control unit.

**Data collection:** The data collection was performed using 1° ω and φ scans, frame times of 1 and s, and a detector distance of 40.00 mm. Overall, 1395 frames were collected with a total exposure time of 0.68 hours. The frames were integrated with the SAINT V8.41 package using a narrow-frame algorithm.⁠^[56]^ The integration of the data using a monoclinic unit cell yielded 119147 reflections to a maximum θ angle of 28.30° (0.75 Å resolution), of which 7282 were independent (average redundancy 16.36, completeness = 100.0%, *R*_int_ = 9.34%, *R*_sig_ = 3.06%) and 6010 (82.5%) were greater than 2σ(*F*^2^). The final cell constants of a = 10.7389(4) Å, b = 19.8380(9) Å, c = 14.1538(7) Å, α = 90°, β = 103.5720(10)°, γ = 90°, volume = 2931.1(2) Å^3^, are based upon the refinement of the XYZ-centroids of 9921 reflections above 20 σ(*I*) with 2.39° < 2θ < 28.16°. Data were corrected for absorption effects using the Multi-Scan method in SADABS 2016/2.^[60]^ The calculated minimum and maximum transmission coefficients (based on crystal size) are 0.679 and 0.746.⁠ Additional crystal and refinement information can be found in the tables.

**Structure solution and refinement:** The space group *P*2_1_/*n* (14) was determined based on intensity statistics and systematic absences. The structure was solved by SHELXT 2018/2 and refined with full-matrix least squares / difference Fourier cycles using SHELXL-2019/2; Z = 4 for the formula unit C_31_H_40_I_2_In_0.25_.⁠^[58,59]^ Non-hydrogen atoms were refined with anisotropic displacement parameters. The hydrogen atoms were placed in ideal positions and refined as riding atoms with relative isotropic displacement parameters. The final anisotropic full-matrix least-squares refinement on *F*^2^ with 298 variables against 7282 data points and converged at *R*_1_ = 3.69%, for the observed data and w*R*_2_ = 14.20% for all data. The goodness-of-fit on *F*^2^ was 1.25. The largest peak in the final difference electron density synthesis was 1.08 e^−^/Å^3^ and the deepest hole was −2.22 e^−^/Å^3^ with an RMS deviation of 0.362 e^−^/Å^3^. On the basis of the final model, the calculated density was 1.57 g/cm^3^ and *F*(000), 1312 e^−^.

# Computational details

All the calculations reported in this paper were performed with the Gaussian 09 suite of programs.^[63]^ Electron correlation was partially taken into account using BP86^[64]^ functional in conjunction with the D3 dispersion correction suggested by Grimme et al.^[65]^ using the standard double-ζ quality def2-SVP^[66]^ basis set for all atoms. Solvents effects were taken into account during the geometry optimizations using the polarizable continuum model (PCM).^[67]^ Reactants, intermediates, and products were characterized by frequency calculations,^[68]^ and have positive definite Hessian matrices. Transition structures (TSs) show only one negative eigenvalue in their diagonalized force constant matrices, and their associated eigenvectors were confirmed to correspond to the motion along the reaction coordinate under consideration using the Intrinsic Reaction Coordinate (IRC) method.^[69]^ Single-point energy refinements were carried out at the same DFT level the much larger triple-ζ quality def2-TZVPP basis sets. This level is denoted PCM-BP86-D3/def2-TZVPP//PCM-BP86-D3/def2-SVP. The computed thermochemistry data were corrected following Grimme’s quasi-harmonic (QHA) model for entropy^[70]^ with a frequency cutoff value of 100.0 cm^−1^ using the GoodVibes^[71]^ program at the specified temperature and a standard concentration of 1 M. Calculations of the absorption spectrum on **1** were accomplished using time-dependent density functional theory (TD-DFT) at the PCM-B3LYP/def2-SVP//PCM-BP86-D3/def2-SVP level.

Wiberg Bond Indices were computed at the PCM-BP86-D3/def2-SVP level using the Natural Bond Orbital (v. 6.0) method^[72]^ while Mayer bond orders were computed using the ORCA 6.0.1^[73]^ program using the optimized PCM-BP86-D3/def2-SVP geometries at the same DFT. QTAIM (Quantum Theory of Atoms in Molecules)^[74]^ calculations on compound **1** were carried out using the optimized PCM-BP86-D3/def2-SVP geometry at the BP86-D3/6-31G*&WTBS (for In) level.

Cartesian coordinates (in Å) and free energies of all the stationary points discussed in the text. All calculations have been performed at the PCM-BP86-D3/def2-TZVPP//PCM-BP86-D3/def2-SVP.

**(InTer)_2_:** G = -2711.442296

In -1.105817000 -0.004490000 0.312555000

In 1.546119000 0.295224000 -1.054088000

C -2.943777000 -0.991066000 -0.610339000

C -4.076296000 -0.257332000 -1.038696000

C -5.193006000 -0.925067000 -1.589732000

H -6.066845000 -0.341344000 -1.923646000

C -5.197819000 -2.327821000 -1.685325000

H -6.076196000 -2.848003000 -2.100191000

C -4.092543000 -3.069736000 -1.232292000

H -4.110976000 -4.171640000 -1.269395000

C -2.963159000 -2.405420000 -0.704264000

C -4.119576000 1.235304000 -0.859840000

C -3.653373000 2.095139000 -1.892856000

C -3.799103000 3.489629000 -1.736105000

H -3.455456000 4.165774000 -2.535545000

C -4.378355000 4.028098000 -0.577499000

H -4.496441000 5.118766000 -0.474909000

C -4.775057000 3.176625000 0.465619000

H -5.189568000 3.609469000 1.390462000

C -4.643744000 1.777311000 0.349115000

C -3.001890000 1.530794000 -3.154130000

H -2.896199000 0.435300000 -3.008463000

C -1.584945000 2.101585000 -3.355093000

H -1.075814000 1.609066000 -4.209789000

H -1.599356000 3.193851000 -3.556554000

H -0.960396000 1.941879000 -2.451025000

C -3.892064000 1.747071000 -4.393721000

H -3.427680000 1.298996000 -5.297585000

H -4.889292000 1.281593000 -4.251160000

H -4.048531000 2.828885000 -4.593576000

C -5.025444000 0.875164000 1.523465000

H -4.895940000 -0.175572000 1.190405000

C -4.067515000 1.095630000 2.712498000

H -4.294846000 0.395551000 3.543338000

H -3.009103000 0.931391000 2.414658000

H -4.138509000 2.131754000 3.107332000

C -6.498629000 1.046481000 1.940105000

H -6.761810000 0.336188000 2.752106000

H -6.703415000 2.071850000 2.315357000

H -7.178289000 0.858057000 1.083587000

C -1.808613000 -3.194408000 -0.142291000

C -0.682900000 -3.542701000 -0.951513000

C 0.346287000 -4.316214000 -0.376144000

H 1.225274000 -4.587541000 -0.977344000

C 0.277187000 -4.737801000 0.960389000

H 1.088570000 -5.349554000 1.386146000

C -0.805585000 -4.354027000 1.761317000

H -0.834860000 -4.660959000 2.818978000

C -1.852226000 -3.568743000 1.232895000

C -0.600486000 -3.109412000 -2.417387000

H -1.057709000 -2.095700000 -2.479245000

C 0.840561000 -3.007764000 -2.947137000

H 0.850774000 -2.505501000 -3.936308000

H 1.500515000 -2.427508000 -2.268456000

H 1.303809000 -4.008467000 -3.081256000

C -1.427392000 -4.049355000 -3.323363000

H -1.366376000 -3.723527000 -4.383336000

H -1.037150000 -5.087658000 -3.259875000

H -2.495445000 -4.064819000 -3.033098000

C -3.009994000 -3.145165000 2.138778000

H -3.648744000 -2.447123000 1.559448000

C -2.515108000 -2.379105000 3.381018000

H -3.372993000 -2.008202000 3.979967000

H -1.893811000 -3.016895000 4.044756000

H -1.898549000 -1.500754000 3.094351000

C -3.886018000 -4.354270000 2.522328000

H -4.748959000 -4.036538000 3.145172000

H -4.281524000 -4.858657000 1.616539000

H -3.306690000 -5.104538000 3.102102000

C 3.057156000 1.016585000 0.552120000

C 2.901572000 2.240179000 1.253111000

C 3.946961000 2.733809000 2.063564000

H 3.825218000 3.692811000 2.593438000

C 5.134762000 1.993445000 2.210899000

H 5.948533000 2.382400000 2.843892000

C 5.276309000 0.747125000 1.573004000

H 6.189136000 0.146329000 1.721529000

C 4.240429000 0.261572000 0.745849000

C 1.552705000 2.896206000 1.154981000

C 1.160269000 3.536713000 -0.061169000

C -0.187640000 3.923156000 -0.221075000

H -0.523006000 4.380646000 -1.163738000

C -1.118114000 3.739138000 0.814127000

H -2.167516000 4.031901000 0.666351000

C -0.710677000 3.165873000 2.026585000

H -1.451234000 3.029346000 2.829726000

C 0.612541000 2.710118000 2.209547000

C 2.212309000 3.880353000 -1.120808000

H 2.975423000 3.071055000 -1.111358000

C 1.666367000 3.983520000 -2.553906000

H 1.116437000 3.066495000 -2.851931000

H 0.976688000 4.845333000 -2.677515000

H 2.500295000 4.127606000 -3.271309000

C 2.941941000 5.178158000 -0.707908000

H 3.741344000 5.432434000 -1.435882000

H 2.228472000 6.028726000 -0.664306000

H 3.408142000 5.070964000 0.292722000

C 1.006206000 2.021926000 3.517767000

H 1.931411000 1.441744000 3.318305000

C -0.051884000 1.025498000 4.027185000

H 0.334578000 0.464781000 4.903584000

H -0.989432000 1.528669000 4.345472000

H -0.313403000 0.287780000 3.240346000

C 1.335172000 3.072466000 4.599981000

H 2.141992000 3.756350000 4.267388000

H 0.441424000 3.692535000 4.827683000

H 1.662712000 2.584024000 5.542315000

C 4.293077000 -1.081895000 0.070558000

C 4.682396000 -1.169582000 -1.299450000

C 4.585958000 -2.414817000 -1.956045000

H 4.878551000 -2.499446000 -3.013655000

C 4.114064000 -3.548276000 -1.279113000

H 4.032533000 -4.511373000 -1.808149000

C 3.740588000 -3.456647000 0.069907000

H 3.370526000 -4.353831000 0.587114000

C 3.821106000 -2.233654000 0.768244000

C 5.244326000 0.056144000 -2.021816000

H 4.822265000 0.952905000 -1.518357000

C 4.849354000 0.135426000 -3.506589000

H 5.163858000 1.108790000 -3.937198000

H 5.333711000 -0.659721000 -4.112303000

H 3.750162000 0.039707000 -3.637188000

C 6.776250000 0.117169000 -1.841468000

H 7.198147000 1.022674000 -2.327154000

H 7.049009000 0.144000000 -0.766684000

H 7.259736000 -0.775112000 -2.294026000

C 3.365981000 -2.136044000 2.227803000

H 3.976377000 -1.338138000 2.703060000

C 1.888375000 -1.692065000 2.322499000

H 1.751562000 -0.676272000 1.901766000

H 1.227893000 -2.392713000 1.770278000

H 1.548804000 -1.664119000 3.379391000

C 3.592876000 -3.430989000 3.028634000

H 3.400543000 -3.251238000 4.106754000

H 2.900684000 -4.238551000 2.709171000

H 4.631284000 -3.807537000 2.920751000

**InTer:** G = -1355.714852

In -0.000141000 0.000199000 -1.793999000

C 0.000035000 -0.000027000 0.555982000

C 1.216632000 0.060219000 1.281575000

C 1.217834000 0.066975000 2.693587000

H 2.173689000 0.119591000 3.241416000

C 0.000079000 -0.000268000 3.395283000

H 0.000101000 -0.000360000 4.497308000

C 2.487542000 0.122695000 0.480696000

C 2.900752000 1.369408000 -0.074197000

C 4.032542000 1.398070000 -0.916898000

H 4.367683000 2.355158000 -1.346923000

C 4.739083000 0.223613000 -1.212719000

H 5.616790000 0.260562000 -1.877860000

C 4.328405000 -0.999790000 -0.660864000

H 4.889468000 -1.915372000 -0.904275000

C 3.207637000 -1.073239000 0.192476000

C 2.172096000 2.664942000 0.285071000

H 1.183363000 2.380636000 0.701372000

C 2.935100000 3.409395000 1.400719000

H 2.396087000 4.332183000 1.703680000

H 3.951886000 3.700682000 1.059809000

H 3.049346000 2.766083000 2.297545000

C 1.912044000 3.574787000 -0.928582000

H 1.276207000 4.436498000 -0.637703000

H 1.389777000 3.028468000 -1.743325000

H 2.851722000 3.986318000 -1.354223000

C 2.733916000 -2.421548000 0.740111000

H 2.148399000 -2.213364000 1.660339000

C 1.776079000 -3.102283000 -0.260637000

H 1.419280000 -4.080783000 0.124703000

H 2.276730000 -3.274243000 -1.237016000

H 0.878890000 -2.473092000 -0.437303000

C 3.885983000 -3.365672000 1.129583000

H 3.488762000 -4.262816000 1.648575000

H 4.608966000 -2.866527000 1.807333000

H 4.446864000 -3.727623000 0.241712000

C -1.216544000 -0.060414000 1.281615000

C -1.217705000 -0.067402000 2.693618000

H -2.173539000 -0.120126000 3.241471000

C -2.487452000 -0.122803000 0.480726000

C -3.207490000 1.073173000 0.192571000

C -4.328177000 0.999846000 -0.660897000

H -4.889181000 1.915478000 -0.904270000

C -4.738830000 -0.223480000 -1.212925000

H -5.616462000 -0.260338000 -1.878170000

C -4.032354000 -1.397995000 -0.917148000

H -4.367474000 -2.355024000 -1.347317000

C -2.900657000 -1.369455000 -0.074326000

C -2.733836000 2.421432000 0.740388000

H -2.148139000 2.213146000 1.660477000

C -3.885982000 3.365307000 1.130244000

H -3.488809000 4.262405000 1.649353000

H -4.447073000 3.727349000 0.242539000

H -4.608776000 2.865916000 1.808012000

C -1.776248000 3.102472000 -0.260388000

H -1.419648000 4.081026000 0.125002000

H -0.878935000 2.473501000 -0.437204000

H -2.277024000 3.274409000 -1.236706000

C -2.172084000 -2.665028000 0.284962000

H -1.183293000 -2.380768000 0.701155000

C -1.912208000 -3.575005000 -0.928620000

H -1.276443000 -4.436755000 -0.637697000

H -2.851948000 -3.986491000 -1.354165000

H -1.389937000 -3.028813000 -1.743447000

C -2.935064000 -3.409293000 1.400749000

H -2.396116000 -4.332102000 1.703762000

H -3.049170000 -2.765876000 2.297518000

H -3.951910000 -3.700506000 1.059955000

**AdCP:** G = -769.54329

P 3.720272000 0.000059000 -0.000308000

C 2.152433000 -0.000254000 0.000943000

C 0.688996000 -0.000289000 0.000967000

C 0.145750000 -0.286380000 -1.438124000

C 0.144489000 -1.103163000 0.967763000

C 0.145136000 1.389143000 0.471686000

H 0.534286000 -1.267732000 -1.785447000

H 0.535221000 0.486669000 -2.134748000

C -1.398746000 -0.284008000 -1.429802000

H 0.532641000 -0.913664000 1.991531000

H 0.533605000 -2.093120000 0.646609000

C -1.400022000 -1.096059000 0.960594000

H 0.533920000 2.180226000 -0.204823000

H 0.533959000 1.606625000 1.489569000

C -1.399368000 1.380320000 0.468491000

H -1.761170000 -0.488900000 -2.460856000

C -1.912631000 -1.380259000 -0.469576000

C -1.912236000 1.096347000 -0.961434000

H -1.763324000 -1.886263000 1.653426000

C -1.912869000 0.284371000 1.429568000

H -1.762284000 2.375599000 0.806326000

H -1.564405000 -2.380686000 -0.809209000

H -3.024554000 -1.403685000 -0.478062000

H -1.564150000 1.890799000 -1.657960000

H -3.024184000 1.114786000 -0.978054000

H -3.024768000 0.289606000 1.454121000

H -1.564466000 0.490239000 2.465752000

**INT0:** G = -2125.258849

In 0.569935000 -0.972992000 -1.028832000

P -0.957951000 -2.086821000 2.204602000

C -2.099902000 -1.873519000 1.145506000

C 1.441351000 0.671776000 0.499904000

C 2.810580000 0.458157000 0.821967000

C 3.538698000 1.365552000 1.620964000

H 4.602304000 1.172963000 1.840197000

C 2.917923000 2.537541000 2.082275000

H 3.481491000 3.264064000 2.689504000

C 3.520170000 -0.691831000 0.157092000

C 4.093384000 -0.469876000 -1.130984000

C 4.717481000 -1.548454000 -1.793389000

H 5.168270000 -1.390368000 -2.786309000

C 4.767329000 -2.820966000 -1.207038000

H 5.254951000 -3.654255000 -1.738056000

C 4.185806000 -3.037307000 0.052083000

H 4.222651000 -4.044061000 0.495367000

C 3.553819000 -1.987518000 0.751410000

C 4.053623000 0.911853000 -1.788090000

H 3.368702000 1.545058000 -1.187667000

C 5.444043000 1.576676000 -1.740222000

H 5.410748000 2.596565000 -2.178798000

H 6.192136000 0.983576000 -2.309053000

H 5.803920000 1.662764000 -0.694214000

C 3.491105000 0.871677000 -3.221334000

H 3.373307000 1.900841000 -3.620718000

H 2.494689000 0.380954000 -3.252527000

H 4.154905000 0.317842000 -3.918515000

C 2.943585000 -2.215997000 2.133730000

H 2.120040000 -1.476266000 2.245020000

C 2.339751000 -3.618865000 2.314957000

H 1.782943000 -3.677550000 3.273061000

H 3.121381000 -4.407771000 2.345253000

H 1.635404000 -3.869673000 1.494062000

C 3.981262000 -1.915514000 3.236775000

H 3.535593000 -2.048161000 4.245439000

H 4.356561000 -0.875345000 3.162318000

H 4.851683000 -2.600922000 3.150622000

C 0.837879000 1.877748000 0.954067000

C 1.585240000 2.800359000 1.727532000

H 1.116323000 3.749514000 2.033635000

C -0.555441000 2.279220000 0.547824000

C -0.818460000 2.622615000 -0.816281000

C -2.101396000 3.089693000 -1.169018000

H -2.314009000 3.358844000 -2.214658000

C -3.109928000 3.242094000 -0.205990000

H -4.103540000 3.616650000 -0.499593000

C -2.850641000 2.899826000 1.127072000

H -3.651197000 3.004107000 1.876173000

C -1.589690000 2.401080000 1.522616000

C 0.291465000 2.596069000 -1.872841000

H 1.008227000 1.800050000 -1.580470000

C 1.090533000 3.915533000 -1.840631000

H 1.929696000 3.885438000 -2.568022000

H 0.437468000 4.776851000 -2.098260000

H 1.516014000 4.094495000 -0.832047000

C -0.200992000 2.269418000 -3.292573000

H 0.664127000 2.104631000 -3.967263000

H -0.826135000 1.351057000 -3.306667000

H -0.802155000 3.094453000 -3.730361000

C -1.380734000 2.000397000 2.984974000

H -0.447746000 1.399171000 3.030652000

C -2.529146000 1.120395000 3.519759000

H -2.270435000 0.712122000 4.519285000

H -3.472005000 1.696121000 3.636388000

H -2.729081000 0.265144000 2.845478000

C -1.198719000 3.237075000 3.890948000

H -1.035005000 2.931688000 4.946242000

H -0.335472000 3.856218000 3.577192000

H -2.104007000 3.880990000 3.859866000

C -3.194272000 -1.649677000 0.201090000

C -3.243536000 -2.779634000 -0.877137000

C -4.558936000 -1.623671000 0.968949000

C -3.015435000 -0.276135000 -0.518909000

H -3.367965000 -3.762729000 -0.374626000

H -2.272895000 -2.805309000 -1.418870000

C -4.407422000 -2.515437000 -1.858106000

H -4.526359000 -0.826164000 1.741481000

H -4.699926000 -2.591206000 1.496662000

C -5.713633000 -1.368489000 -0.024551000

H -2.049868000 -0.270791000 -1.072651000

H -2.954113000 0.533361000 0.232547000

C -4.172332000 -0.028784000 -1.503387000

H -4.425416000 -3.329641000 -2.615018000

C -5.744276000 -2.495245000 -1.082245000

C -4.198315000 -1.154186000 -2.560495000

H -6.671119000 -1.358429000 0.540771000

C -5.504703000 -0.005727000 -0.723331000

H -4.003749000 0.954959000 -1.991854000

H -5.915543000 -3.477659000 -0.589279000

H -6.590278000 -2.333472000 -1.785873000

H -3.243498000 -1.161212000 -3.131555000

H -5.015491000 -0.974488000 -3.293227000

H -6.350729000 0.201896000 -1.414852000

H -5.486057000 0.814506000 0.027893000

**TS-In:** G = -2125.249812 (i = -97.7 cm-1)

In -0.003970000 0.028433000 -1.506472000

P -0.432259000 -0.908943000 1.360142000

C -1.585379000 -0.872088000 0.219517000

C 1.475191000 0.421856000 0.614912000

C 2.688519000 -0.295504000 0.832647000

C 3.722227000 0.277882000 1.602846000

H 4.654501000 -0.288336000 1.761896000

C 3.578371000 1.564512000 2.151401000

H 4.384421000 1.998118000 2.764185000

C 2.875185000 -1.630426000 0.168878000

C 3.270778000 -1.660605000 -1.204093000

C 3.367752000 -2.908043000 -1.854597000

H 3.670215000 -2.950159000 -2.912249000

C 3.098041000 -4.102490000 -1.168764000

H 3.173345000 -5.068531000 -1.693676000

C 2.748585000 -4.066191000 0.188213000

H 2.562265000 -5.010434000 0.723372000

C 2.634426000 -2.840116000 0.878149000

C 3.691803000 -0.376777000 -1.925959000

H 3.130938000 0.463212000 -1.462400000

C 5.192362000 -0.105419000 -1.679673000

H 5.507487000 0.842966000 -2.164746000

H 5.810650000 -0.929121000 -2.096568000

H 5.413694000 -0.027103000 -0.596571000

C 3.380205000 -0.365725000 -3.432779000

H 3.559600000 0.645849000 -3.852566000

H 2.322237000 -0.634507000 -3.636196000

H 4.024241000 -1.072755000 -3.997754000

C 2.336526000 -2.831084000 2.378230000

H 1.972705000 -1.812751000 2.635501000

C 1.246604000 -3.835365000 2.795624000

H 0.974938000 -3.688162000 3.861766000

H 1.588507000 -4.886911000 2.688633000

H 0.327301000 -3.711592000 2.186630000

C 3.635761000 -3.071064000 3.178047000

H 3.441980000 -3.028970000 4.271041000

H 4.406297000 -2.311601000 2.936009000

H 4.061765000 -4.070119000 2.942900000

C 1.376699000 1.761540000 1.103317000

C 2.418487000 2.310575000 1.880314000

H 2.327492000 3.343237000 2.255092000

C 0.205823000 2.602433000 0.668884000

C 0.276821000 3.249445000 -0.605876000

C -0.864397000 3.926607000 -1.088145000

H -0.829200000 4.423415000 -2.069703000

C -2.042270000 3.984016000 -0.329070000

H -2.928292000 4.505932000 -0.724801000

C -2.085301000 3.393596000 0.942327000

H -3.005781000 3.471054000 1.540924000

C -0.971161000 2.702936000 1.462838000

C 1.596189000 3.305742000 -1.384657000

H 2.179664000 2.398265000 -1.116536000

C 2.420051000 4.527433000 -0.920495000

H 3.398783000 4.565128000 -1.444162000

H 1.874003000 5.469929000 -1.139603000

H 2.615037000 4.492238000 0.169438000

C 1.434081000 3.319843000 -2.915048000

H 2.424633000 3.213583000 -3.403823000

H 0.790864000 2.487623000 -3.270669000

H 0.991492000 4.271314000 -3.279054000

C -1.002116000 2.146277000 2.885499000

H -0.257869000 1.320159000 2.928118000

C -2.365019000 1.566066000 3.299666000

H -2.283565000 1.054948000 4.281255000

H -3.138514000 2.355890000 3.407032000

H -2.728766000 0.826897000 2.557695000

C -0.543032000 3.231161000 3.884653000

H -0.520789000 2.830789000 4.920407000

H 0.470738000 3.605285000 3.638469000

H -1.237906000 4.098026000 3.864757000

C -3.010347000 -1.130099000 -0.139651000

C -3.123196000 -2.082239000 -1.369293000

C -3.789510000 -1.777274000 1.047363000

C -3.705821000 0.220677000 -0.504897000

H -2.621927000 -3.045769000 -1.131993000

H -2.578197000 -1.635276000 -2.230828000

C -4.604707000 -2.314202000 -1.740022000

H -3.711508000 -1.111995000 1.934664000

H -3.297846000 -2.737203000 1.317808000

C -5.269477000 -2.007778000 0.674312000

H -3.164792000 0.692600000 -1.355276000

H -3.616468000 0.923509000 0.350858000

C -5.185995000 -0.015708000 -0.873624000

H -4.652689000 -2.994776000 -2.618278000

C -5.347711000 -2.953174000 -0.545110000

C -5.265032000 -0.961877000 -2.093054000

H -5.796462000 -2.467079000 1.539478000

C -5.925322000 -0.652863000 0.324568000

H -5.653531000 0.961411000 -1.126127000

H -4.893525000 -3.937318000 -0.294538000

H -6.409837000 -3.144533000 -0.815220000

H -4.751280000 -0.502104000 -2.966251000

H -6.326161000 -1.120605000 -2.386834000

H -6.999621000 -0.799926000 0.075757000

H -5.885022000 0.028263000 1.203358000

**1:** G = -2125.27553

In 1.232138000 0.275834000 -2.249139000

P -0.123898000 0.122735000 1.066063000

C 1.292696000 0.328845000 0.143990000

C -1.393931000 -0.280285000 -0.261384000

C -2.251783000 0.747361000 -0.756352000

C -3.174115000 0.454164000 -1.782268000

H -3.824895000 1.257875000 -2.161573000

C -3.288301000 -0.850447000 -2.288757000

H -4.009573000 -1.071936000 -3.091071000

C -2.278486000 2.105409000 -0.106286000

C -1.623805000 3.228486000 -0.682077000

C -1.698633000 4.475013000 -0.026913000

H -1.194437000 5.350568000 -0.465381000

C -2.410102000 4.618871000 1.172228000

H -2.456895000 5.599101000 1.673241000

C -3.070124000 3.512876000 1.725597000

H -3.643678000 3.635660000 2.657946000

C -3.020451000 2.249017000 1.103313000

C -0.888353000 3.123976000 -2.012939000

H -0.749877000 2.034881000 -2.217013000

C -1.747374000 3.673900000 -3.169104000

H -1.228347000 3.547866000 -4.142797000

H -1.949346000 4.756637000 -3.023474000

H -2.724708000 3.153602000 -3.227839000

C 0.502912000 3.782769000 -1.986275000

H 1.055225000 3.570022000 -2.926832000

H 1.105414000 3.413857000 -1.131009000

H 0.433173000 4.886919000 -1.897295000

C -3.821809000 1.087923000 1.692073000

H -3.492082000 0.157969000 1.187061000

C -3.579265000 0.894957000 3.198893000

H -4.130658000 0.003874000 3.564368000

H -3.932011000 1.763308000 3.794500000

H -2.500370000 0.745420000 3.410040000

C -5.320715000 1.258095000 1.368609000

H -5.906087000 0.392434000 1.745394000

H -5.483653000 1.333774000 0.273414000

H -5.728946000 2.179383000 1.836965000

C -1.584113000 -1.625025000 -0.700787000

C -2.522608000 -1.882873000 -1.727447000

H -2.666110000 -2.921253000 -2.064774000

C -0.902536000 -2.798727000 -0.043295000

C 0.054634000 -3.578850000 -0.752394000

C 0.605593000 -4.724114000 -0.137942000

H 1.344151000 -5.330831000 -0.685490000

C 0.229621000 -5.100534000 1.155095000

H 0.668003000 -5.996457000 1.623213000

C -0.710090000 -4.329001000 1.854004000

H -1.000818000 -4.636509000 2.868727000

C -1.293016000 -3.180990000 1.281682000

C 0.517525000 -3.229471000 -2.165205000

H 0.112059000 -2.219397000 -2.408714000

C -0.058529000 -4.200705000 -3.214963000

H 0.257059000 -3.908598000 -4.238764000

H 0.299471000 -5.235934000 -3.029512000

H -1.166546000 -4.220212000 -3.188045000

C 2.055029000 -3.158944000 -2.269620000

H 2.364331000 -2.759975000 -3.259203000

H 2.486193000 -2.515057000 -1.474199000

H 2.519147000 -4.161629000 -2.163816000

C -2.360493000 -2.407092000 2.062862000

H -2.201678000 -1.328085000 1.855737000

C -2.248786000 -2.565547000 3.587814000

H -2.958139000 -1.879497000 4.092236000

H -2.499096000 -3.594122000 3.924329000

H -1.227125000 -2.320540000 3.944729000

C -3.779690000 -2.762726000 1.572389000

H -4.540777000 -2.158281000 2.110293000

H -3.896386000 -2.566470000 0.487778000

H -4.000232000 -3.836326000 1.754074000

C 2.620594000 0.603914000 0.842120000

C 3.184076000 1.982791000 0.377761000

C 2.564240000 0.619669000 2.397599000

C 3.642873000 -0.497373000 0.417354000

H 2.469008000 2.780950000 0.674296000

H 3.249188000 2.008046000 -0.735298000

C 4.579511000 2.249375000 0.984540000

H 2.166152000 -0.354846000 2.758394000

H 1.844741000 1.400447000 2.730783000

C 3.956445000 0.885701000 3.010863000

H 3.723201000 -0.521264000 -0.694816000

H 3.254650000 -1.491083000 0.730476000

C 5.036951000 -0.232823000 1.027010000

H 4.947186000 3.236858000 0.627928000

C 4.477515000 2.256547000 2.525840000

C 5.555919000 1.138023000 0.537377000

H 3.872763000 0.889226000 4.120036000

C 4.933149000 -0.226027000 2.568113000

H 5.733973000 -1.036063000 0.700819000

H 3.790463000 3.065582000 2.859063000

H 5.473624000 2.470493000 2.973265000

H 5.651120000 1.136428000 -0.571598000

H 6.570786000 1.332772000 0.949291000

H 5.937075000 -0.058317000 3.017805000

H 4.574948000 -1.214913000 2.930445000

**(GaTer)_2_:** G = -6181.165844

Ga 1.061954000 0.245745000 0.408266000

Ga -1.188124000 -0.531590000 -0.598160000

C 2.519630000 1.248776000 -0.618315000

C 3.756833000 0.696100000 -1.036517000

C 4.734254000 1.516926000 -1.643076000

H 5.686096000 1.067096000 -1.969957000

C 4.504923000 2.892161000 -1.812074000

H 5.276258000 3.528018000 -2.275390000

C 3.295821000 3.457336000 -1.373075000

H 3.117310000 4.540638000 -1.472690000

C 2.305889000 2.642482000 -0.782860000

C 4.082371000 -0.756450000 -0.830468000

C 3.810764000 -1.691939000 -1.865654000

C 4.247850000 -3.023938000 -1.709728000

H 4.053335000 -3.757722000 -2.508696000

C 4.930712000 -3.426556000 -0.552244000

H 5.274958000 -4.468220000 -0.448983000

C 5.152189000 -2.507848000 0.486281000

H 5.662640000 -2.840342000 1.404283000

C 4.734553000 -1.166332000 0.367688000

C 3.059002000 -1.272508000 -3.127282000

H 2.818077000 -0.194026000 -3.025911000

C 1.719859000 -2.023054000 -3.240478000

H 1.126024000 -1.655561000 -4.103606000

H 1.870016000 -3.115585000 -3.374832000

H 1.111529000 -1.879885000 -2.323984000

C 3.924413000 -1.433554000 -4.392029000

H 3.378047000 -1.076269000 -5.290440000

H 4.865640000 -0.851687000 -4.307222000

H 4.198420000 -2.496096000 -4.566523000

C 4.958427000 -0.183083000 1.517962000

H 4.746075000 0.834224000 1.126975000

C 3.954004000 -0.447714000 2.657357000

H 4.056877000 0.303469000 3.468492000

H 2.905886000 -0.399975000 2.287338000

H 4.100636000 -1.457040000 3.098523000

C 6.410134000 -0.182247000 2.032296000

H 6.550511000 0.608867000 2.798552000

H 6.683243000 -1.149253000 2.505720000

H 7.127897000 0.005102000 1.207181000

C 1.046638000 3.286120000 -0.268568000

C -0.093644000 3.424164000 -1.116748000

C -1.195058000 4.166927000 -0.646647000

H -2.083928000 4.283573000 -1.282341000

C -1.179047000 4.756733000 0.626346000

H -2.044075000 5.347154000 0.967653000

C -0.082065000 4.564950000 1.477052000

H -0.099518000 4.994376000 2.491475000

C 1.036560000 3.816893000 1.054253000

C -0.102787000 2.824013000 -2.523218000

H 0.559262000 1.930563000 -2.497686000

C -1.497448000 2.353474000 -2.967113000

H -1.427069000 1.761003000 -3.902620000

H -1.978876000 1.713428000 -2.198217000

H -2.185953000 3.202095000 -3.163399000

C 0.485919000 3.816682000 -3.549919000

H 0.494541000 3.369472000 -4.566546000

H -0.124743000 4.744224000 -3.587890000

H 1.525324000 4.101263000 -3.293279000

C 2.206544000 3.588298000 2.012184000

H 2.931023000 2.920418000 1.500783000

C 1.747934000 2.858802000 3.290701000

H 2.615724000 2.618631000 3.940314000

H 1.039063000 3.472137000 3.886336000

H 1.235163000 1.905416000 3.041307000

C 2.942197000 4.904001000 2.331140000

H 3.816697000 4.717861000 2.990023000

H 3.306726000 5.389255000 1.402238000

H 2.275271000 5.624866000 2.850805000

C -2.661361000 -1.298365000 0.618586000

C -2.415886000 -2.530529000 1.277050000

C -3.436711000 -3.169014000 2.008171000

H -3.235982000 -4.132348000 2.504008000

C -4.707922000 -2.572506000 2.098707000

H -5.509595000 -3.070900000 2.667018000

C -4.959527000 -1.341819000 1.465121000

H -5.953882000 -0.871202000 1.537687000

C -3.942742000 -0.705989000 0.719666000

C -1.018026000 -3.057106000 1.151743000

C -0.613987000 -3.661185000 -0.076457000

C 0.754763000 -3.951198000 -0.265499000

H 1.095452000 -4.399148000 -1.211624000

C 1.691337000 -3.678766000 0.741794000

H 2.756174000 -3.888012000 0.570308000

C 1.276401000 -3.125562000 1.962241000

H 2.027079000 -2.919315000 2.739845000

C -0.073146000 -2.788180000 2.184528000

C -1.658602000 -4.100566000 -1.107801000

H -2.603774000 -3.567032000 -0.872581000

C -1.295605000 -3.759008000 -2.562372000

H -1.127247000 -2.668853000 -2.691865000

H -0.377698000 -4.282894000 -2.901642000

H -2.118909000 -4.056153000 -3.244835000

C -1.938666000 -5.609263000 -0.934222000

H -2.735192000 -5.947253000 -1.630757000

H -1.025119000 -6.207535000 -1.139669000

H -2.265296000 -5.835979000 0.101730000

C -0.517135000 -2.166922000 3.509047000

H -1.489582000 -1.663813000 3.323113000

C 0.450355000 -1.098479000 4.046731000

H 0.023249000 -0.608815000 4.946536000

H 1.432730000 -1.526177000 4.338682000

H 0.633199000 -0.316135000 3.281584000

C -0.757848000 -3.274292000 4.557990000

H -1.505882000 -4.012572000 4.204216000

H 0.185067000 -3.823780000 4.767269000

H -1.123664000 -2.842986000 5.514000000

C -4.197008000 0.591371000 0.008967000

C -4.562129000 0.564602000 -1.367006000

C -4.817473000 1.786024000 -2.024996000

H -5.106236000 1.782810000 -3.088044000

C -4.699711000 3.003910000 -1.343952000

H -4.899988000 3.951494000 -1.869749000

C -4.312289000 3.022256000 0.005330000

H -4.210828000 3.989609000 0.517426000

C -4.050438000 1.827890000 0.706538000

C -4.709939000 -0.756257000 -2.124939000

H -4.317709000 -1.561277000 -1.468956000

C -3.874977000 -0.785724000 -3.419386000

H -3.930772000 -1.786936000 -3.895900000

H -4.229453000 -0.040692000 -4.162923000

H -2.806372000 -0.567657000 -3.207610000

C -6.195857000 -1.069823000 -2.392649000

H -6.308061000 -2.047485000 -2.907803000

H -6.769137000 -1.111028000 -1.443373000

H -6.660195000 -0.291207000 -3.035297000

C -3.596910000 1.848915000 2.170872000

H -4.100700000 0.993740000 2.673543000

C -2.071880000 1.612125000 2.295154000

H -1.795381000 0.581282000 2.001671000

H -1.505791000 2.318601000 1.655438000

H -1.732222000 1.756781000 3.342406000

C -4.000012000 3.128931000 2.923685000

H -3.783031000 3.017154000 4.006165000

H -3.424100000 4.009353000 2.568493000

H -5.080615000 3.355555000 2.811072000

**GaTer:** G = -3090.573072

Ga -0.005225000 -0.033762000 -1.691165000

C -0.002939000 0.026170000 0.408119000

C 1.214107000 0.103686000 1.129358000

C 1.211994000 0.154288000 2.539683000

H 2.166881000 0.218771000 3.087179000

C -0.008006000 0.110549000 3.238954000

H -0.010133000 0.142587000 4.340481000

C 2.487059000 0.118614000 0.329560000

C 2.965884000 1.346230000 -0.210432000

C 4.118595000 1.326075000 -1.024296000

H 4.505221000 2.268910000 -1.443475000

C 4.777580000 0.121969000 -1.310197000

H 5.672855000 0.122380000 -1.952502000

C 4.292820000 -1.085356000 -0.783432000

H 4.810634000 -2.027109000 -1.024925000

C 3.149736000 -1.109574000 0.041633000

C 2.267845000 2.671056000 0.097382000

H 1.321417000 2.430950000 0.625522000

C 3.118989000 3.529361000 1.055277000

H 2.593080000 4.472463000 1.315233000

H 4.093369000 3.798984000 0.594204000

H 3.330852000 2.981240000 1.996556000

C 1.893359000 3.444915000 -1.180750000

H 1.293528000 4.344984000 -0.932091000

H 1.292052000 2.816676000 -1.873012000

H 2.791510000 3.783985000 -1.739222000

C 2.593255000 -2.438506000 0.555382000

H 1.883598000 -2.203455000 1.376165000

C 1.787585000 -3.147019000 -0.552432000

H 1.342655000 -4.095179000 -0.183939000

H 2.428137000 -3.378900000 -1.429439000

H 0.950944000 -2.509065000 -0.911192000

C 3.677858000 -3.365779000 1.134985000

H 3.214167000 -4.264322000 1.593251000

H 4.274745000 -2.849487000 1.914832000

H 4.379981000 -3.724428000 0.352654000

C -1.223775000 -0.012003000 1.129044000

C -1.226219000 0.022211000 2.539024000

H -2.182241000 -0.015962000 3.087259000

C -2.489353000 -0.098199000 0.322696000

C -3.230404000 1.085199000 0.033463000

C -4.357895000 0.987076000 -0.807278000

H -4.937289000 1.890713000 -1.051037000

C -4.752266000 -0.246780000 -1.347871000

H -5.636082000 -0.302679000 -2.003480000

C -4.021586000 -1.406566000 -1.055884000

H -4.343293000 -2.370488000 -1.480106000

C -2.882300000 -1.355071000 -0.224408000

C -2.767507000 2.444368000 0.563764000

H -2.216966000 2.257214000 1.509989000

C -3.923027000 3.408462000 0.887131000

H -3.535377000 4.312460000 1.401189000

H -4.443804000 3.757022000 -0.029973000

H -4.678001000 2.933190000 1.546991000

C -1.769065000 3.091220000 -0.420348000

H -1.420796000 4.078112000 -0.049002000

H -0.869975000 2.454074000 -0.550495000

H -2.234147000 3.238249000 -1.417929000

C -2.130879000 -2.638498000 0.134414000

H -1.129519000 -2.342979000 0.511762000

C -1.905129000 -3.567803000 -1.071288000

H -1.249837000 -4.417023000 -0.787531000

H -2.854818000 -3.998551000 -1.453242000

H -1.419550000 -3.028059000 -1.912265000

C -2.851140000 -3.365856000 1.289171000

H -2.296881000 -4.280161000 1.590252000

H -2.939075000 -2.706868000 2.177600000

H -3.876908000 -3.668256000 0.987302000

**INT0-Ga:** G = -3860.119918

Ga 0.585535000 -0.870209000 -0.865470000

P -0.681670000 -1.737455000 2.258815000

C -1.826309000 -1.749701000 1.179925000

C 1.388586000 0.656063000 0.403789000

C 2.773195000 0.475870000 0.675662000

C 3.519115000 1.438052000 1.387198000

H 4.593543000 1.272992000 1.571806000

C 2.897811000 2.626070000 1.804954000

H 3.474417000 3.394762000 2.344103000

C 3.461975000 -0.707592000 0.047422000

C 3.943553000 -0.571109000 -1.289428000

C 4.553331000 -1.682651000 -1.908225000

H 4.932415000 -1.591665000 -2.938642000

C 4.680308000 -2.903436000 -1.231147000

H 5.154944000 -3.763679000 -1.729958000

C 4.196229000 -3.033407000 0.079700000

H 4.297219000 -3.999329000 0.597851000

C 3.581915000 -1.948210000 0.738334000

C 3.833547000 0.756659000 -2.042904000

H 3.169830000 1.421850000 -1.453043000

C 5.208712000 1.449628000 -2.122532000

H 5.127114000 2.432776000 -2.632796000

H 5.937622000 0.829798000 -2.687597000

H 5.623883000 1.620947000 -1.107892000

C 3.192913000 0.596067000 -3.434286000

H 3.023472000 1.589274000 -3.900335000

H 2.212453000 0.077461000 -3.368724000

H 3.834008000 0.009547000 -4.125935000

C 3.093086000 -2.080469000 2.179411000

H 2.292856000 -1.320619000 2.317423000

C 2.489548000 -3.459332000 2.497672000

H 2.014128000 -3.449963000 3.500480000

H 3.261894000 -4.257738000 2.511817000

H 1.716768000 -3.746858000 1.754441000

C 4.228932000 -1.732604000 3.165628000

H 3.873371000 -1.794339000 4.216057000

H 4.611112000 -0.706581000 2.992648000

H 5.079313000 -2.438018000 3.047015000

C 0.781337000 1.878897000 0.806517000

C 1.547571000 2.852298000 1.491927000

H 1.079365000 3.813071000 1.759284000

C -0.631877000 2.239994000 0.431026000

C -0.953247000 2.496263000 -0.939616000

C -2.249453000 2.945091000 -1.265330000

H -2.505951000 3.150777000 -2.315226000

C -3.219403000 3.148909000 -0.273032000

H -4.225036000 3.505425000 -0.547377000

C -2.907796000 2.876827000 1.064976000

H -3.679821000 3.016131000 1.837735000

C -1.628480000 2.408889000 1.437701000

C 0.100319000 2.377549000 -2.046162000

H 0.814486000 1.582039000 -1.743086000

C 0.930238000 3.674195000 -2.141030000

H 1.730316000 3.575581000 -2.905158000

H 0.283653000 4.532598000 -2.423113000

H 1.409486000 3.908766000 -1.168642000

C -0.471916000 1.965282000 -3.412175000

H 0.354015000 1.738666000 -4.116934000

H -1.107258000 1.058235000 -3.327094000

H -1.082707000 2.769957000 -3.873515000

C -1.361381000 2.088530000 2.910235000

H -0.429034000 1.485194000 2.950963000

C -2.491160000 1.246287000 3.538511000

H -2.187750000 0.879855000 4.541590000

H -3.420659000 1.838892000 3.674984000

H -2.733699000 0.365213000 2.912484000

C -1.138599000 3.370874000 3.740630000

H -0.937426000 3.121062000 4.804000000

H -0.284548000 3.966490000 3.363062000

H -2.041231000 4.018329000 3.708337000

C -2.937752000 -1.714267000 0.229473000

C -2.868876000 -2.911830000 -0.771275000

C -4.297577000 -1.778146000 1.002962000

C -2.901638000 -0.379528000 -0.579227000

H -2.893231000 -3.867123000 -0.204280000

H -1.899695000 -2.872034000 -1.313980000

C -4.052746000 -2.832732000 -1.760277000

H -4.348160000 -0.931912000 1.721045000

H -4.339373000 -2.718126000 1.593968000

C -5.471713000 -1.708341000 0.001438000

H -1.936928000 -0.312821000 -1.132039000

H -2.928540000 0.476026000 0.121636000

C -4.075577000 -0.317046000 -1.571586000

H -3.986364000 -3.692167000 -2.462576000

C -5.385682000 -2.899038000 -0.980287000

C -3.983631000 -1.506432000 -2.552148000

H -6.425657000 -1.759391000 0.570617000

C -5.404502000 -0.380438000 -0.787318000

H -4.008345000 0.644715000 -2.124916000

H -5.455813000 -3.859567000 -0.423523000

H -6.243311000 -2.870825000 -1.687956000

H -3.031834000 -1.452790000 -3.125523000

H -4.813694000 -1.460785000 -3.291098000

H -6.266939000 -0.308607000 -1.486052000

H -5.472639000 0.484903000 -0.091628000

**TS1-Ga:** G = -3860.119407 (i = -115.1 cm-1)

Ga -0.227495000 0.310759000 -0.618146000

P -0.344701000 -2.684668000 0.578088000

C -1.657468000 -1.866361000 0.211573000

C 1.768790000 0.660243000 0.058572000

C 2.943598000 -0.133212000 -0.059271000

C 4.218583000 0.460300000 0.096817000

H 5.120584000 -0.161860000 -0.028303000

C 4.345474000 1.820479000 0.425516000

H 5.345233000 2.263918000 0.559383000

C 2.853195000 -1.612165000 -0.297469000

C 2.480291000 -2.112839000 -1.576291000

C 2.353455000 -3.507657000 -1.754437000

H 2.065194000 -3.905946000 -2.740054000

C 2.603197000 -4.393697000 -0.698918000

H 2.496708000 -5.479686000 -0.851637000

C 2.995277000 -3.895944000 0.554007000

H 3.187806000 -4.603928000 1.373755000

C 3.127268000 -2.511552000 0.780505000

C 2.298419000 -1.182350000 -2.775887000

H 2.228558000 -0.145175000 -2.386990000

C 3.548576000 -1.230844000 -3.679265000

H 3.450488000 -0.524696000 -4.531115000

H 3.698802000 -2.250724000 -4.094139000

H 4.460451000 -0.960004000 -3.107899000

C 1.011563000 -1.466178000 -3.573335000

H 0.852495000 -0.684714000 -4.345402000

H 0.118089000 -1.479660000 -2.911593000

H 1.052643000 -2.445353000 -4.095972000

C 3.478024000 -1.983559000 2.175926000

H 4.097252000 -1.073171000 2.035918000

C 2.201250000 -1.540547000 2.921629000

H 2.452353000 -1.067215000 3.894678000

H 1.539975000 -2.410550000 3.120758000

H 1.625186000 -0.810014000 2.320400000

C 4.300700000 -2.962154000 3.030630000

H 4.636487000 -2.462935000 3.963280000

H 5.200577000 -3.324606000 2.491373000

H 3.706186000 -3.849441000 3.336593000

C 1.921130000 2.039372000 0.388112000

C 3.195236000 2.609311000 0.592104000

H 3.280914000 3.671512000 0.875753000

C 0.658635000 2.852054000 0.502018000

C 0.260780000 3.718963000 -0.562173000

C -1.025823000 4.293397000 -0.516525000

H -1.361339000 4.942328000 -1.339777000

C -1.897676000 4.042326000 0.555045000

H -2.904278000 4.490264000 0.561495000

C -1.480002000 3.237714000 1.622918000

H -2.156548000 3.071600000 2.476505000

C -0.200241000 2.641043000 1.622649000

C 1.176598000 3.968007000 -1.764871000

H 2.221243000 3.839090000 -1.413214000

C 1.059104000 5.394482000 -2.332909000

H 1.851245000 5.570691000 -3.089991000

H 0.085124000 5.562852000 -2.839719000

H 1.165245000 6.160823000 -1.537376000

C 0.942280000 2.916590000 -2.870129000

H 1.599522000 3.107239000 -3.744518000

H 1.163510000 1.891935000 -2.504432000

H -0.113186000 2.932120000 -3.214748000

C 0.264831000 1.846334000 2.843800000

H 1.255611000 1.412456000 2.598785000

C -0.672499000 0.676964000 3.191246000

H -0.278822000 0.105786000 4.057491000

H -1.692838000 1.028070000 3.454592000

H -0.770828000 -0.031597000 2.343191000

C 0.464708000 2.789676000 4.048929000

H 0.870989000 2.231978000 4.919218000

H 1.171798000 3.608573000 3.802541000

H -0.494638000 3.254503000 4.362506000

C -3.070946000 -1.477609000 0.017299000

C -3.401702000 -1.228502000 -1.487552000

C -3.969158000 -2.657539000 0.525795000

C -3.440792000 -0.201802000 0.838009000

H -3.139624000 -2.136868000 -2.072133000

H -2.761782000 -0.400463000 -1.861125000

C -4.896057000 -0.881683000 -1.654830000

H -3.737617000 -2.853930000 1.594744000

H -3.712549000 -3.578035000 -0.041211000

C -5.463024000 -2.305700000 0.346927000

H -2.802056000 0.646766000 0.505679000

H -3.211068000 -0.379761000 1.909811000

C -4.933385000 0.140227000 0.652480000

H -5.101102000 -0.702094000 -2.732923000

C -5.762486000 -2.056091000 -1.147877000

C -5.221769000 0.393396000 -0.844202000

H -6.073256000 -3.159397000 0.715353000

C -5.799298000 -1.034966000 1.158787000

H -5.162944000 1.056880000 1.238824000

H -5.547200000 -2.972874000 -1.740006000

H -6.841749000 -1.825091000 -1.286902000

H -4.607489000 1.245294000 -1.210172000

H -6.288853000 0.673867000 -0.985568000

H -6.879067000 -0.789546000 1.052439000

H -5.610374000 -1.210295000 2.240968000

**INT1-Ga:** G = -3860.13295

Ga 0.271381000 -0.113749000 0.647489000

P -0.558797000 -0.639679000 2.742379000

C -1.440391000 -0.690919000 1.241937000

C 1.705896000 0.550166000 -0.481174000

C 2.814698000 -0.257747000 -0.822971000

C 3.853874000 0.303304000 -1.594752000

H 4.717616000 -0.321672000 -1.872263000

C 3.782184000 1.644142000 -2.015454000

H 4.599782000 2.072613000 -2.616442000

C 2.804492000 -1.682260000 -0.355390000

C 2.290613000 -2.696758000 -1.213810000

C 2.180433000 -4.010002000 -0.710640000

H 1.775845000 -4.807453000 -1.352900000

C 2.572225000 -4.314823000 0.601046000

H 2.475473000 -5.345729000 0.977865000

C 3.084643000 -3.310349000 1.435901000

H 3.390449000 -3.564949000 2.461798000

C 3.207131000 -1.982131000 0.979924000

C 1.808580000 -2.362997000 -2.627848000

H 2.317915000 -1.426048000 -2.937316000

C 2.169216000 -3.443461000 -3.664267000

H 1.921125000 -3.092121000 -4.687521000

H 1.602091000 -4.383959000 -3.498776000

H 3.250762000 -3.689809000 -3.635965000

C 0.291616000 -2.076007000 -2.631450000

H -0.064431000 -1.813575000 -3.649918000

H 0.040613000 -1.228705000 -1.960734000

H -0.280552000 -2.960441000 -2.279726000

C 3.814104000 -0.896794000 1.870769000

H 3.375909000 0.073771000 1.552574000

C 3.493415000 -1.067819000 3.364878000

H 3.839881000 -0.179881000 3.933363000

H 4.002249000 -1.952619000 3.803290000

H 2.401817000 -1.176202000 3.529059000

C 5.337318000 -0.810965000 1.630162000

H 5.789425000 0.000114000 2.239569000

H 5.565479000 -0.607850000 0.563923000

H 5.830599000 -1.767021000 1.907730000

C 1.621037000 1.895234000 -0.913677000

C 2.669376000 2.438892000 -1.681692000

H 2.605333000 3.483521000 -2.025199000

C 0.376513000 2.647922000 -0.548361000

C -0.721439000 2.647327000 -1.456047000

C -1.914242000 3.291899000 -1.070473000

H -2.771890000 3.308731000 -1.761305000

C -2.033394000 3.890846000 0.192040000

H -2.978758000 4.375836000 0.483207000

C -0.956563000 3.862181000 1.090642000

H -1.067266000 4.328693000 2.081153000

C 0.263818000 3.247230000 0.741179000

C -0.612035000 1.976327000 -2.825518000

H 0.298812000 1.341461000 -2.811379000

C -0.417283000 3.035190000 -3.930363000

H -0.296344000 2.554576000 -4.924258000

H -1.292429000 3.717730000 -3.984084000

H 0.482179000 3.654642000 -3.734279000

C -1.800057000 1.045922000 -3.131033000

H -1.638789000 0.513296000 -4.091215000

H -1.921231000 0.282992000 -2.336006000

H -2.758415000 1.600612000 -3.216168000

C 1.460996000 3.277580000 1.693325000

H 2.104567000 2.406072000 1.444752000

C 1.074395000 3.147701000 3.176292000

H 1.984773000 3.032232000 3.800158000

H 0.536609000 4.045564000 3.548252000

H 0.427871000 2.261853000 3.346632000

C 2.301267000 4.547570000 1.437170000

H 3.197520000 4.568402000 2.092709000

H 2.643970000 4.594723000 0.383163000

H 1.702146000 5.460561000 1.641842000

C -2.861149000 -0.967065000 0.866312000

C -2.915532000 -1.937346000 -0.349906000

C -3.698013000 -1.569102000 2.025143000

C -3.508681000 0.389562000 0.439045000

H -2.440876000 -2.902914000 -0.068434000

H -2.309624000 -1.513764000 -1.179540000

C -4.376036000 -2.157929000 -0.797938000

H -3.659616000 -0.885635000 2.902395000

H -3.241198000 -2.530740000 2.348515000

C -5.159847000 -1.788651000 1.574739000

H -2.905999000 0.843694000 -0.374637000

H -3.463255000 1.098613000 1.294059000

C -4.967718000 0.166919000 -0.011237000

H -4.389644000 -2.856834000 -1.663229000

C -5.187970000 -2.759467000 0.371783000

C -4.993504000 -0.803279000 -1.214186000

H -5.742340000 -2.221665000 2.417554000

C -5.780432000 -0.435527000 1.157005000

H -5.404975000 1.145546000 -0.309544000

H -4.760540000 -3.743666000 0.665905000

H -6.238180000 -2.943425000 0.053473000

H -4.423563000 -0.369627000 -2.065259000

H -6.039318000 -0.951200000 -1.563793000

H -6.840696000 -0.579699000 0.851748000

H -5.783074000 0.265673000 2.020754000

**TS2-Ga:** G = -3860.119407 (i = -118.7 cm-1)

Ga 0.008509000 0.017475000 -1.157651000

P -0.260925000 -0.887067000 1.379864000

C -1.356073000 -0.908061000 0.160323000

C 1.402427000 0.529803000 0.485189000

C 2.700042000 -0.057478000 0.623816000

C 3.737289000 0.660343000 1.248933000

H 4.730108000 0.191556000 1.343698000

C 3.524118000 1.963146000 1.735822000

H 4.339524000 2.508371000 2.236229000

C 2.938822000 -1.416878000 0.034746000

C 3.189446000 -1.516470000 -1.367745000

C 3.338152000 -2.796519000 -1.939928000

H 3.535262000 -2.891959000 -3.018836000

C 3.251414000 -3.953713000 -1.150732000

H 3.365148000 -4.946774000 -1.614744000

C 3.033704000 -3.845501000 0.229982000

H 2.987999000 -4.759314000 0.842819000

C 2.875290000 -2.585071000 0.844840000

C 3.404553000 -0.258850000 -2.214371000

H 2.843788000 0.568038000 -1.727922000

C 4.897094000 0.136795000 -2.193985000

H 5.066478000 1.071559000 -2.769787000

H 5.520471000 -0.664627000 -2.645331000

H 5.253049000 0.302361000 -1.157046000

C 2.884258000 -0.376548000 -3.657159000

H 2.933604000 0.610208000 -4.162650000

H 1.827395000 -0.715668000 -3.678216000

H 3.487107000 -1.085621000 -4.263675000

C 2.716721000 -2.485735000 2.362691000

H 2.291426000 -1.482636000 2.584334000

C 1.760247000 -3.539174000 2.951011000

H 1.576059000 -3.335149000 4.026424000

H 2.179964000 -4.565251000 2.881095000

H 0.781587000 -3.535464000 2.427974000

C 4.098933000 -2.558666000 3.047599000

H 4.002310000 -2.449732000 4.148820000

H 4.774800000 -1.760059000 2.680869000

H 4.587395000 -3.535002000 2.840389000

C 1.223098000 1.888727000 0.909103000

C 2.275194000 2.578636000 1.541711000

H 2.117659000 3.622099000 1.859594000

C -0.054054000 2.585203000 0.526428000

C -0.143830000 3.141877000 -0.789333000

C -1.372711000 3.696673000 -1.207313000

H -1.459951000 4.127407000 -2.216458000

C -2.481966000 3.718727000 -0.350272000

H -3.436032000 4.148685000 -0.695033000

C -2.373728000 3.206606000 0.951033000

H -3.245688000 3.249995000 1.621404000

C -1.169561000 2.633997000 1.408895000

C 1.093971000 3.244831000 -1.686878000

H 1.791962000 2.432886000 -1.388379000

C 1.817664000 4.582776000 -1.418472000

H 2.741913000 4.660439000 -2.029374000

H 1.159957000 5.440362000 -1.676129000

H 2.101375000 4.679979000 -0.351555000

C 0.801550000 3.073251000 -3.187728000

H 1.752710000 3.019533000 -3.756935000

H 0.234440000 2.139534000 -3.384635000

H 0.221976000 3.925687000 -3.601637000

C -1.044808000 2.137262000 2.847690000

H -0.222470000 1.386915000 2.861806000

C -2.312546000 1.445438000 3.377907000

H -2.115164000 0.986754000 4.368842000

H -3.151781000 2.160533000 3.510899000

H -2.651276000 0.643490000 2.690398000

C -0.619546000 3.297026000 3.774411000

H -0.485434000 2.941192000 4.817892000

H 0.334499000 3.751687000 3.440005000

H -1.392315000 4.095408000 3.778620000

C -2.759163000 -1.293180000 -0.182974000

C -2.801411000 -2.180213000 -1.463488000

C -3.457065000 -2.065983000 0.973608000

C -3.571909000 0.011055000 -0.466620000

H -2.214453000 -3.107515000 -1.284638000

H -2.307121000 -1.638782000 -2.300619000

C -4.261827000 -2.522138000 -1.833324000

H -3.426553000 -1.442838000 1.894533000

H -2.880018000 -2.991963000 1.189613000

C -4.915977000 -2.406632000 0.601433000

H -3.082580000 0.579410000 -1.288635000

H -3.535068000 0.667332000 0.428626000

C -5.029996000 -0.334330000 -0.836413000

H -4.262563000 -3.155865000 -2.747183000

C -4.927923000 -3.287957000 -0.667854000

C -5.043121000 -1.216255000 -2.105231000

H -5.389459000 -2.956754000 1.444166000

C -5.693468000 -1.098257000 0.331830000

H -5.583973000 0.610551000 -1.029937000

H -4.385163000 -4.239995000 -0.476325000

H -5.973010000 -3.558241000 -0.937063000

H -4.583877000 -0.666564000 -2.956397000

H -6.090394000 -1.451923000 -2.397114000

H -6.753896000 -1.326993000 0.085159000

H -5.701448000 -0.464379000 1.246169000

**1-Ga:** G = -3860.139352

Ga 1.117316000 -0.237203000 2.161366000

P -0.044355000 -0.149013000 -0.922321000

C 1.340548000 -0.333789000 0.053672000

C -1.361613000 0.275472000 0.353154000

C -2.251016000 -0.740907000 0.811527000

C -3.246278000 -0.422775000 1.757471000

H -3.925256000 -1.216327000 2.107052000

C -3.397637000 0.895148000 2.217851000

H -4.177238000 1.136568000 2.957375000

C -2.216771000 -2.111468000 0.192091000

C -1.532084000 -3.194591000 0.808572000

C -1.534124000 -4.452713000 0.172536000

H -1.006946000 -5.299195000 0.639453000

C -2.202046000 -4.644010000 -1.045892000

H -2.192371000 -5.633247000 -1.531097000

C -2.886737000 -3.575530000 -1.641365000

H -3.419746000 -3.735912000 -2.592044000

C -2.908094000 -2.300440000 -1.039918000

C -0.847482000 -3.023954000 2.159122000

H -0.682646000 -1.926299000 2.302372000

C -1.768607000 -3.475230000 3.310277000

H -1.285261000 -3.300442000 4.294585000

H -1.994631000 -4.559577000 3.222650000

H -2.731845000 -2.927009000 3.296196000

C 0.526420000 -3.710825000 2.242454000

H 1.049402000 -3.416795000 3.177132000

H 1.167844000 -3.432015000 1.382101000

H 0.435999000 -4.817163000 2.253936000

C -3.714965000 -1.172854000 -1.684749000

H -3.458251000 -0.229261000 -1.161132000

C -3.373297000 -0.971091000 -3.171632000

H -3.929987000 -0.101118000 -3.578075000

H -3.648762000 -1.853544000 -3.787009000

H -2.288523000 -0.781081000 -3.305943000

C -5.224967000 -1.402747000 -1.468298000

H -5.816119000 -0.561689000 -1.889008000

H -5.462788000 -1.484975000 -0.387397000

H -5.562278000 -2.339549000 -1.961612000

C -1.580531000 1.630046000 0.743640000

C -2.591631000 1.914397000 1.689085000

H -2.757715000 2.960878000 1.988569000

C -0.836896000 2.774967000 0.106990000

C 0.125105000 3.518327000 0.846489000

C 0.751959000 4.627563000 0.239414000

H 1.495089000 5.208888000 0.807663000

C 0.445232000 4.999113000 -1.074266000

H 0.943289000 5.866671000 -1.536158000

C -0.498515000 4.260024000 -1.802692000

H -0.731994000 4.562446000 -2.833895000

C -1.154830000 3.148120000 -1.238096000

C 0.499211000 3.160297000 2.282805000

H 0.084194000 2.145343000 2.493153000

C -0.145882000 4.126143000 3.297022000

H 0.107260000 3.833020000 4.337683000

H 0.218864000 5.163053000 3.134335000

H -1.250063000 4.140694000 3.202316000

C 2.024691000 3.085997000 2.492505000

H 2.259925000 2.654191000 3.487910000

H 2.510514000 2.460276000 1.714984000

H 2.496547000 4.089931000 2.448356000

C -2.221517000 2.402162000 -2.046472000

H -2.150045000 1.328387000 -1.776488000

C -2.012168000 2.480020000 -3.567378000

H -2.729555000 1.811406000 -4.083890000

H -2.182424000 3.504044000 -3.962552000

H -0.987017000 2.164221000 -3.850362000

C -3.640470000 2.868760000 -1.658221000

H -4.407342000 2.281268000 -2.206897000

H -3.826411000 2.739943000 -0.573041000

H -3.783064000 3.942056000 -1.907243000

C 2.698203000 -0.621019000 -0.576423000

C 3.234907000 -1.991219000 -0.056727000

C 2.710225000 -0.664878000 -2.130212000

C 3.700129000 0.487290000 -0.120885000

H 2.532700000 -2.793865000 -0.371328000

H 3.247496000 -1.993344000 1.058136000

C 4.655970000 -2.270668000 -0.594111000

H 2.331362000 0.303437000 -2.527219000

H 2.005418000 -1.451297000 -2.481725000

C 4.128943000 -0.944248000 -2.672856000

H 3.728990000 0.528070000 0.992935000

H 3.328760000 1.475961000 -0.468451000

C 5.119950000 0.209627000 -0.661971000

H 5.004469000 -3.251736000 -0.202605000

C 4.623739000 -2.306522000 -2.138052000

C 5.613409000 -1.152388000 -0.123815000

H 4.097003000 -0.968647000 -3.784343000

C 5.086175000 0.174707000 -2.205741000

H 5.802499000 1.018130000 -0.318966000

H 3.950844000 -3.120739000 -2.487115000

H 5.638560000 -2.530143000 -2.535750000

H 5.658567000 -1.130145000 0.987894000

H 6.645246000 -1.355943000 -0.486129000

H 6.109143000 -0.002377000 -2.606031000

H 4.746887000 1.157133000 -2.602227000

**INT2-Ga:** G = -4629.719431

Ga 0.249359000 0.410199000 -0.367856000

C -0.096932000 -1.508167000 -0.784711000

P -1.757287000 -1.841827000 -0.704033000

C -2.587064000 -0.123509000 -0.732941000

P -1.812557000 1.408846000 -0.887200000

C 0.965803000 -2.541304000 -1.073402000

C 2.147051000 -2.419748000 -0.072326000

H 2.533175000 -1.376290000 -0.066071000

H 1.753006000 -2.612551000 0.943518000

C 1.521360000 -2.234886000 -2.499744000

H 1.878495000 -1.184665000 -2.523107000

H 0.694615000 -2.308718000 -3.239045000

C 0.466765000 -4.011805000 -1.055639000

H 0.071592000 -4.251521000 -0.043412000

H -0.382511000 -4.124248000 -1.766010000

C 3.284732000 -3.397496000 -0.428079000

H 4.101992000 -3.285418000 0.318297000

C 2.668341000 -3.200593000 -2.864702000

H 3.040512000 -2.945608000 -3.881443000

C 1.606341000 -4.984903000 -1.432714000

H 1.215114000 -6.025854000 -1.418693000

C 3.815429000 -3.059693000 -1.838836000

H 4.655633000 -3.736701000 -2.109137000

H 4.215839000 -2.021836000 -1.853164000

C 2.133372000 -4.649580000 -2.846778000

H 2.940321000 -5.360356000 -3.132188000

H 1.318307000 -4.763969000 -3.595165000

C 2.755363000 -4.848293000 -0.410774000

H 3.576186000 -5.558767000 -0.654496000

H 2.390815000 -5.113754000 0.606124000

C -4.117564000 -0.240091000 -0.675301000

C -4.641103000 -1.145011000 -1.838526000

H -4.361923000 -0.677180000 -2.808125000

H -4.141417000 -2.137486000 -1.803371000

C -4.527735000 -0.896458000 0.684166000

H -4.015795000 -1.876962000 0.802895000

H -4.173912000 -0.247166000 1.513110000

C -4.848513000 1.125409000 -0.778547000

H -4.496469000 1.797122000 0.034532000

H -4.573244000 1.620830000 -1.735675000

C -6.172012000 -1.328980000 -1.744008000

H -6.504891000 -1.983574000 -2.579388000

C -6.057958000 -1.082309000 0.765181000

H -6.309794000 -1.556788000 1.739274000

C -6.380196000 0.946562000 -0.691758000

H -6.863000000 1.945563000 -0.767199000

C -6.529621000 -1.989181000 -0.393252000

H -7.628014000 -2.152330000 -0.327136000

H -6.047008000 -2.988511000 -0.316587000

C -6.748019000 0.294456000 0.659431000

H -6.425694000 0.948691000 1.499688000

H -7.851517000 0.178425000 0.742054000

C -6.865281000 0.046020000 -1.848503000

H -7.970339000 -0.076327000 -1.803218000

H -6.629567000 0.519828000 -2.826924000

C 1.733193000 1.152474000 0.737764000

C 2.570069000 2.137437000 0.154828000

C 3.664088000 2.650322000 0.882643000

H 4.305329000 3.424491000 0.432561000

C 3.902636000 2.207461000 2.193375000

H 4.752089000 2.612689000 2.765656000

C 3.029002000 1.289671000 2.800473000

H 3.186841000 1.013761000 3.852462000

C 1.930130000 0.755676000 2.090797000

C 2.135050000 2.726915000 -1.159384000

C 2.562980000 2.195866000 -2.412668000

C 2.016834000 2.743902000 -3.593516000

H 2.324402000 2.350418000 -4.573109000

C 1.079311000 3.785863000 -3.544356000

H 0.657861000 4.189080000 -4.479083000

C 0.678478000 4.314025000 -2.309719000

H -0.053416000 5.135768000 -2.283362000

C 1.195803000 3.802966000 -1.103078000

C 3.625144000 1.094123000 -2.479285000

H 3.403856000 0.375392000 -1.656123000

C 3.634047000 0.314360000 -3.806549000

H 4.295747000 -0.571762000 -3.723179000

H 2.625179000 -0.041733000 -4.096325000

H 4.026333000 0.936080000 -4.638763000

C 5.035838000 1.668725000 -2.217239000

H 5.107902000 2.151329000 -1.224676000

H 5.798830000 0.862814000 -2.259462000

H 5.291577000 2.426461000 -2.988041000

C 0.806874000 4.441710000 0.232902000

H 0.998269000 3.693048000 1.029651000

C -0.675374000 4.841590000 0.333257000

H -1.344273000 3.994115000 0.078636000

H -0.909705000 5.166473000 1.368610000

H -0.927034000 5.689234000 -0.338938000

C 1.724020000 5.651055000 0.518682000

H 1.488502000 6.100345000 1.506759000

H 2.792800000 5.356424000 0.520690000

H 1.589993000 6.434716000 -0.257610000

C 0.893808000 -0.150110000 2.699619000

C 1.207400000 -1.475869000 3.136369000

C 0.147780000 -2.367338000 3.404746000

H 0.372841000 -3.404489000 3.694629000

C -1.188363000 -1.958358000 3.305381000

H -2.002964000 -2.678162000 3.482678000

C -1.485333000 -0.616184000 3.033208000

H -2.534231000 -0.291687000 3.045138000

C -0.465769000 0.316469000 2.752352000

C 2.648030000 -1.905671000 3.426709000

H 3.314144000 -1.354810000 2.727525000

C 2.933010000 -3.411507000 3.266437000

H 2.593953000 -3.807138000 2.290673000

H 4.022968000 -3.601151000 3.348365000

H 2.441023000 -4.009607000 4.062129000

C 3.008146000 -1.493199000 4.876196000

H 2.797302000 -0.424707000 5.077706000

H 2.403118000 -2.082421000 5.597723000

H 4.081168000 -1.684620000 5.088927000

C -0.797133000 1.819118000 2.673745000

H -0.335503000 2.232882000 1.752449000

C -2.298189000 2.139331000 2.611237000

H -2.795703000 1.630221000 1.762194000

H -2.818732000 1.856952000 3.551282000

H -2.439844000 3.229733000 2.468031000

C -0.159587000 2.567591000 3.866403000

H 0.940419000 2.444875000 3.893976000

H -0.377471000 3.654434000 3.798414000

H -0.573454000 2.192117000 4.826530000

**2:** G = -7720.356745

Ga -1.620146000 0.392417000 0.142152000

Ga 1.200030000 -0.384471000 0.256007000

C -0.330565000 -0.460683000 -1.131550000

P 0.561549000 0.969529000 -1.904448000

C 0.800740000 1.988364000 -0.475111000

P 0.143825000 1.572342000 1.219633000

C -0.720882000 -1.651189000 -2.009687000

C -2.084338000 -1.365078000 -2.685980000

H -2.849587000 -1.141805000 -1.910736000

H -1.975635000 -0.447070000 -3.295815000

C -0.888602000 -2.914901000 -1.129347000

H -1.624899000 -2.702197000 -0.321885000

H 0.077381000 -3.133864000 -0.621899000

C 0.312020000 -1.974219000 -3.121005000

H 0.456399000 -1.082223000 -3.768540000

H 1.293008000 -2.187522000 -2.653550000

C -2.549107000 -2.556173000 -3.545562000

H -3.526973000 -2.309067000 -4.014390000

C -1.347594000 -4.121731000 -1.972232000

H -1.452613000 -5.011114000 -1.314458000

C -0.145818000 -3.173737000 -3.975201000

H 0.622516000 -3.373834000 -4.753296000

C -2.706335000 -3.794578000 -2.633546000

H -3.064607000 -4.667210000 -3.223467000

H -3.472666000 -3.592154000 -1.853309000

C -0.300230000 -4.413003000 -3.069376000

H -0.612732000 -5.296190000 -3.670213000

H 0.678547000 -4.660342000 -2.608563000

C -1.499898000 -2.846103000 -4.640885000

H -1.833416000 -3.695546000 -5.277854000

H -1.393246000 -1.962031000 -5.308111000

C 1.739020000 3.202314000 -0.606002000

C 3.129269000 2.858903000 0.012099000

H 2.990559000 2.566679000 1.072412000

H 3.550758000 1.970004000 -0.503668000

C 1.952113000 3.607390000 -2.088841000

H 2.345793000 2.743682000 -2.659519000

H 0.968290000 3.864638000 -2.539011000

C 1.181484000 4.448208000 0.141337000

H 0.189976000 4.705624000 -0.282552000

H 1.015416000 4.196034000 1.209662000

C 4.096425000 4.055497000 -0.105071000

H 5.073706000 3.773244000 0.345307000

C 2.926636000 4.796953000 -2.211529000

H 3.060669000 5.042407000 -3.288347000

C 2.146166000 5.648817000 0.021077000

H 1.712071000 6.514687000 0.568198000

C 4.290217000 4.411576000 -1.594959000

H 5.010595000 5.253064000 -1.699244000

H 4.722209000 3.543566000 -2.139837000

C 2.340008000 6.014774000 -1.466089000

H 1.365644000 6.303315000 -1.919686000

H 3.017680000 6.892097000 -1.562860000

C 3.512128000 5.276674000 0.638150000

H 4.212723000 6.138343000 0.568142000

H 3.389742000 5.043476000 1.716293000

C -3.616176000 0.316566000 0.229806000

C -4.321676000 -0.442458000 1.196870000

C -5.737301000 -0.423270000 1.195656000

H -6.278330000 -0.981840000 1.974865000

C -6.446732000 0.335191000 0.255671000

H -7.547948000 0.348322000 0.272836000

C -5.748063000 1.123865000 -0.672139000

H -6.305290000 1.778663000 -1.357328000

C -4.338788000 1.131698000 -0.694509000

C -3.646931000 -1.173460000 2.326358000

C -3.665547000 -2.599089000 2.374655000

C -3.225765000 -3.245932000 3.548391000

H -3.243563000 -4.345061000 3.602637000

C -2.761153000 -2.511918000 4.645982000

H -2.431142000 -3.032727000 5.559403000

C -2.691824000 -1.114003000 4.571729000

H -2.300313000 -0.548492000 5.428827000

C -3.123238000 -0.419041000 3.424602000

C -4.165353000 -3.442313000 1.198768000

H -4.124777000 -2.796758000 0.293024000

C -3.278228000 -4.677662000 0.944815000

H -3.550129000 -5.155417000 -0.018439000

H -2.204277000 -4.411996000 0.910074000

H -3.409802000 -5.445235000 1.736069000

C -5.629645000 -3.893810000 1.391437000

H -6.322156000 -3.035350000 1.479307000

H -5.963147000 -4.513498000 0.532138000

H -5.728595000 -4.506301000 2.313126000

C -3.100382000 1.113440000 3.414229000

H -2.920171000 1.434195000 2.367997000

C -1.966601000 1.722191000 4.254546000

H -0.982861000 1.280570000 3.998890000

H -1.905341000 2.813600000 4.068564000

H -2.131443000 1.583290000 5.344629000

C -4.469477000 1.695668000 3.824239000

H -4.450226000 2.805010000 3.768689000

H -5.281369000 1.336554000 3.160651000

H -4.721801000 1.406373000 4.866896000

C -3.572028000 2.107447000 -1.550496000

C -3.430598000 1.967829000 -2.967382000

C -2.563760000 2.851004000 -3.645073000

H -2.411058000 2.735801000 -4.728225000

C -1.902743000 3.887390000 -2.971477000

H -1.225488000 4.559223000 -3.522345000

C -2.142786000 4.094921000 -1.608031000

H -1.688473000 4.960644000 -1.104677000

C -2.980126000 3.226994000 -0.878196000

C -4.290311000 0.993389000 -3.778080000

H -4.499951000 0.110574000 -3.136163000

C -3.652091000 0.506094000 -5.095680000

H -2.612113000 0.150825000 -4.964434000

H -4.245899000 -0.329984000 -5.518211000

H -3.638089000 1.310480000 -5.860873000

C -5.640818000 1.666972000 -4.128030000

H -6.182223000 2.026420000 -3.233474000

H -5.467546000 2.546762000 -4.783467000

H -6.303691000 0.959565000 -4.669832000

C -3.419879000 3.605933000 0.540690000

H -3.708156000 2.677607000 1.072045000

C -2.346411000 4.299201000 1.390493000

H -1.426870000 3.682155000 1.456852000

H -2.066967000 5.296666000 0.989954000

H -2.726972000 4.457905000 2.420711000

C -4.696857000 4.471438000 0.444006000

H -5.501963000 3.941636000 -0.104986000

H -5.077104000 4.721804000 1.457116000

H -4.485760000 5.421180000 -0.092418000

C 3.097121000 -0.842649000 0.665031000

C 3.462688000 -0.702269000 2.040611000

C 4.806722000 -0.895331000 2.431807000

H 5.066160000 -0.815724000 3.498313000

C 5.792692000 -1.219833000 1.490935000

H 6.840145000 -1.352000000 1.804996000

C 5.418515000 -1.450721000 0.161193000

H 6.164580000 -1.807260000 -0.566435000

C 4.074624000 -1.306752000 -0.260046000

C 2.455117000 -0.516280000 3.143804000

C 1.527843000 -1.570817000 3.421290000

C 0.677374000 -1.455304000 4.537873000

H -0.037024000 -2.259931000 4.758274000

C 0.731198000 -0.338530000 5.380373000

H 0.061014000 -0.268635000 6.251684000

C 1.630433000 0.695728000 5.100039000

H 1.654994000 1.579655000 5.756159000

C 2.495621000 0.632064000 3.988579000

C 1.506486000 -2.880248000 2.626250000

H 1.879675000 -2.675233000 1.602232000

C 0.100827000 -3.481532000 2.466099000

H 0.126371000 -4.328856000 1.751076000

H -0.308605000 -3.871683000 3.420352000

H -0.624174000 -2.731835000 2.085616000

C 2.497999000 -3.877877000 3.260394000

H 2.527654000 -4.829821000 2.690800000

H 3.524005000 -3.455092000 3.278623000

H 2.203457000 -4.108420000 4.306394000

C 3.441635000 1.807374000 3.741004000

H 3.878893000 1.679372000 2.727687000

C 2.694507000 3.155758000 3.761644000

H 3.375995000 3.977639000 3.460161000

H 1.826929000 3.141964000 3.071109000

H 2.318057000 3.401397000 4.776748000

C 4.602604000 1.829981000 4.758337000

H 5.196635000 0.894660000 4.734262000

H 5.289860000 2.677108000 4.549777000

H 4.216420000 1.953290000 5.792766000

C 3.760087000 -1.901920000 -1.609591000

C 3.516421000 -3.310054000 -1.633301000

C 3.365430000 -3.957443000 -2.877467000

H 3.192735000 -5.044484000 -2.906912000

C 3.445710000 -3.239723000 -4.076286000

H 3.324480000 -3.757373000 -5.041063000

C 3.677889000 -1.857498000 -4.045559000

H 3.730772000 -1.306804000 -4.995282000

C 3.847654000 -1.166064000 -2.827431000

C 3.511226000 -4.155500000 -0.355748000

H 3.435010000 -3.466199000 0.508429000

C 2.316824000 -5.122713000 -0.274641000

H 2.331645000 -5.676754000 0.686269000

H 1.353676000 -4.579444000 -0.339027000

H 2.333438000 -5.879188000 -1.086820000

C 4.845278000 -4.917708000 -0.208045000

H 4.865997000 -5.490301000 0.743438000

H 4.987167000 -5.635145000 -1.044554000

H 5.708106000 -4.221685000 -0.208190000

C 4.167940000 0.329779000 -2.833305000

H 3.632944000 0.769842000 -1.962340000

C 3.675249000 1.055454000 -4.096140000

H 3.840533000 2.147644000 -3.997357000

H 4.232483000 0.732354000 -5.000710000

H 2.592910000 0.885166000 -4.267736000

C 5.678533000 0.587480000 -2.642315000

H 5.890160000 1.677833000 -2.653126000

H 6.050009000 0.184374000 -1.681636000

H 6.260701000 0.118119000 -3.463870000

# References

[56] SAINT V8.41, Bruker AXS, Madison, WI, USA, 2024.

[57] Sheldrick, G. M. (2012). *TWINABS* 2012/1. Bruker, Madison, Wisconsin, USA.

[58] G. M. Sheldrick, “SHELXT—Integrated space-group and crystal-structure determination” *Acta Cryst.* **2015**, *A71*, 3–8.

[59] G. M. Sheldrick, “Crystal structure refinement with SHELXL” *Acta Cryst.* **2015**, *C71*, 3–8.

[60] L. Krause, R. Herbst-Irmer, G. M. Sheldrick, D. Stalke, “Comparison of silver and molybdenum microfocus X-ray sources for single-crystal structure determination” *J. Appl. Crystallogr.* **2015**, *48*, 3–10.

[61] Spek, A. L. “PLATON SQUEEZE: A tool for the calculation of the disordered solvent contribution to the calculated structure factors” *Acta Cryst.* **2015**, *C71*, 9–18.

[62] A. Immirzi, B. Perini, “Prediction of density in organic crystals” *Acta Cryst.* **1977**, *A33*, 216–218.

[63] Gaussian 09, Revision A.02, M. J. Frisch, G. W. Trucks, H. B. Schlegel, G. E. Scuseria, M. A. Robb, J. R. Cheeseman, G. Scalmani, V. Barone, G. A. Petersson, H. Nakatsuji, X. Li, M. Caricato, A. Marenich, J. Bloino, B. G. Janesko, R. Gomperts, B. Mennucci, H. P. Hratchian, J. V. Ortiz, A. F. Izmaylov, J. L. Sonnenberg, D. Williams-Young, F. Ding, F. Lipparini, F. Egidi, J. Goings, B. Peng, A. Petrone, T. Henderson, D. Ranasinghe, V. G. Zakrzewski, J. Gao, N. Rega, G. Zheng, W. Liang, M. Hada, M. Ehara, K. Toyota, R. Fukuda, J. Hasegawa, M. Ishida, T. Nakajima, Y. Honda, O. Kitao, H. Nakai, T. Vreven, K. Throssell, J. A. Montgomery, Jr., J. E. Peralta, F. Ogliaro, M. Bearpark, J. J. Heyd, E. Brothers, K. N. Kudin, V. N. Staroverov, T. Keith, R. Kobayashi, J. Normand, K. Raghavachari, A. Rendell, J. C. Burant, S. S. Iyengar, J. Tomasi, M. Cossi, J. M. Millam, M. Klene, C. Adamo, R. Cammi, J. W. Ochterski, R. L. Martin, K. Morokuma, O. Farkas, J. B. Foresman, and D. J. Fox, Gaussian, Inc., Wallingford CT, 2016.

[64] (a) A. D. Becke, “Density‐functional thermochemistry. III. The role of exact exchange” *J. Chem. Phys.* **1993**, *98*, 5648; (b) J. P. Perdew, “Density-functional approximation for the correlation energy of the inhomogeneous electron gas” *Phys. Rev. B* **1986**, *33*, 8822.

[65] S. Grimme, J. Antony, S. Ehrlich, H. Krieg, “A consistent and accurate ab initio parametrization of density functional dispersion correction (DFT-D) for the 94 elements H-Pu” *J. Chem. Phys.* **2010**, *132*, 154104.

[66] F. Weigend, R. Ahlrichs, “Balanced basis sets of split valence, triple zeta valence and quadruple zeta valence quality for H to Rn: Design and assessment of accuracy” *Phys. Chem. Chem. Phys.* **2005**, *7*, 3297

[67] (a) S. Miertuš, E. Scrocco, J. Tomasi, “Electrostatic interaction of a solute with a continuum. A direct utilizaion of AB initio molecular potentials for the prevision of solvent effects” *Chem. Phys.* **1981**, *55*, 117; (b) J. L. Pascual-Ahuir, E. Silla, I. Tuñón, “GEPOL: An improved description of molecular surfaces. III. A new algorithm for the computation of a solvent-excluding surface” *J. Comp. Chem.* **1994**, *15*, 1127; (c) V. Barone; M. Cossi, “Quantum Calculation of Molecular Energies and Energy Gradients in Solution by a Conductor Solvent Model” *J. Phys. Chem. A* **1998**, *102*, 1995.

[68] J. W. McIver, A. K. Komornicki, “Structure of transition states in organic reactions. General theory and an application to the cyclobutene-butadiene isomerization using a semiempirical molecular orbital method” *J. Am. Chem. Soc.* **1972**, *94*, 2625.

[69] C. González, H. B. Schlegel, “Reaction path following in mass-weighted internal coordinates” *J. Phys. Chem.* **1990**, *94*, 5523.

[70] S. Grimme, “Supramolecular Binding Thermodynamics by Dispersion-Corrected Density Functional Theory” *Chem. Eur. J.* **2012**, *18*, 9955.

[71] G. Luchini, J. V. Alegre-Requena, I. Funes-Ardoiz, R. S. Paton, “GoodVibes: automated thermochemistry for heterogeneous computational chemistry data” *F1000 Research* **2020**, *9*, 291.

[72] E. D. Glendening, C. R. Landis, F. Weinhold, *J. Comput. Chem*. **2013**, *34*, 1429.

[73] F. Neese, “The ORCA program system” *Rev. Comput. Mol. Sci.* **2012**, *2*, 73.

[74] R. F. W. Bader, *Atoms in molecules. A quantum theory;* Clarendon Press: Oxford, U.K., 1990.
